# Description and Cross-Sectional Analyses of 25,880 Adults and Children in the UK National Registry of Rare Kidney Diseases Cohort

**DOI:** 10.1016/j.ekir.2024.04.062

**Published:** 2024-05-09

**Authors:** Katie Wong, David Pitcher, Fiona Braddon, Lewis Downward, Retha Steenkamp, Sherry Masoud, Nicholas Annear, Jonathan Barratt, Coralie Bingham, Richard J. Coward, Tina Chrysochou, David Game, Sian Griffin, Matt Hall, Sally Johnson, Durga Kanigicherla, Fiona Karet Frankl, David Kavanagh, Larissa Kerecuk, Eamonn R. Maher, Shabbir Moochhala, Jenny Pinney, John A. Sayer, Roslyn Simms, Smeeta Sinha, Shalabh Srivastava, Frederick W.K. Tam, Kay Thomas, A. Neil Turner, Stephen B. Walsh, Aoife Waters, Patricia Wilson, Edwin Wong, Karla Therese L. Sy, Kui Huang, Jamie Ye, Dorothea Nitsch, Moin Saleem, Detlef Bockenhauer, Kate Bramham, Daniel P. Gale, RaDaR consortium, Sharirose Abat, Shazia Adalat, Joy Agbonmwandolor, Zubaidah Ahmad, Abdulfattah Alejmi, Rashid Almasarwah, Nicholas Annear, Ellie Asgari, Amanda Ayers, Jyoti Baharani, Gowrie Balasubramaniam, Felix Jo-Bamba Kpodo, Tarun Bansal, Alison Barratt, Jonathan Barratt, Megan Bates, Natalie Bayne, Janet Bendle, Sarah Benyon, Carsten Bergmann, Sunil Bhandari, Coralie Bingham, Preetham Boddana, Sally Bond, Fiona Braddon, Kate Bramham, Angela Branson, Stephen Brearey, Vicky Brocklebank, Sharanjit Budwal, Conor Byrne, Hugh Cairns, Brian Camilleri, Gary Campbell, Alys Capell, Margaret Carmody, Marion Carson, Tracy Cathcart, Christine Catley, Karine Cesar, Melanie Chan, Houda Chea, James Chess, Chee Kay Cheung, Katy-Jane Chick, Nihil Chitalia, Martin Christian, Tina Chrysochou, Katherine Clark, Christopher Clayton, Rhian Clissold, Helen Cockerill, Joshua Coelho, Elizabeth Colby, Viv Colclough, Eileen Conway, H. Terence Cook, Wendy Cook, Theresa Cooper, Richard J. Coward, Sarah Crosbie, Gabor Cserep, Anjali Date, Katherine Davidson, Amanda Davies, Neeraj Dhaun, Ajay Dhaygude, Lynn Diskin, Abhijit Dixit, Eunice Ann Doctolero, Suzannah Dorey, Lewis Downard, Mark Drayson, Gavin Dreyer, Tina Dutt, Kufreabasi Etuk, Dawn Evans, Jenny Finch, Frances Flinter, James Fotheringham, Lucy Francis, Daniel P. Gale, Hugh Gallagher, David Game, Eva Lozano Garcia, Madita Gavrila, Susie Gear, Colin Geddes, Mark Gilchrist, Matt Gittus, Paraskevi Goggolidou, Christopher Goldsmith, Patricia Gooden, Andrea Goodlife, Priyanka Goodwin, Tassos Grammatikopoulos, Barry Gray, Megan Griffith, Steph Gumus, Sanjana Gupta, Patrick Hamilton, Lorraine Harper, Tess Harris, Louise Haskell, Samantha Hayward, Shivaram Hegde, Bruce Hendry, Sue Hewins, Nicola Hewitson, Kate Hillman, Mrityunjay Hiremath, Alexandra Howson, Zay Htet, Sharon Huish, Richard Hull, Alister Humphries, David P.J. Hunt, Karl Hunter, Samantha Hunter, Marilyn Ijeomah-Orji, Nick Inston, David Jayne, Gbemisola Jenfa, Alison Jenkins, Sally Johnson, Caroline A. Jones, Colin Jones, Amanda Jones, Rachel Jones, Lavanya Kamesh, Durga Kanigicherla, Fiona Karet Frankl, Mahzuz Karim, Amrit Kaur, David Kavanagh, Kelly Kearley, Larissa Kerecuk, Arif Khwaja, Garry King, Grant King, Ewa Kislowska, Edyta Klata, Maria Kokocinska, Mark Lambie, Laura Lawless, Thomas Ledson, Rachel Lennon, Adam P. Levine, Ling Wai Maggie Lai, Graham Lipkin, Graham Lovitt, Paul Lyons, Holly Mabillard, Katherine Mackintosh, Khalid Mahdi, Eamonn Maher, Kevin J. Marchbank, Patrick B. Mark, Sherry Masoud, Bridgett Masunda, Zainab Mavani, Jake Mayfair, Stephen McAdoo, Joanna Mckinnell, Nabil Melhem, Simon Meyrick, Shabbir Moochhala, Putnam Morgan, Ann Morgan, Fawad Muhammad, Shona Murray, Kristina Novobritskaya, Albert CM. Ong, Louise Oni, Kate Osmaston, Neal Padmanabhan, Sharon Parkes, Jean Patrick, James Pattison, Riny Paul, Rachel Percival, Stephen J. Perkins, Alexandre Persu, William G. Petchey, Matthew C. Pickering, Jennifer Pinney, David Pitcher, Lucy Plumb, Zoe Plummer, Joyce Popoola, Frank Post, Albert Power, Guy Pratt, Charles Pusey, Ria Rabara, May Rabuya, Tina Raju, Chadd Javier, Ian SD. Roberts, Candice Roufosse, Adam Rumjon, Alan Salama, Moin Saleem, R.N. Sandford, Kanwaljit S. Sandu, Nadia Sarween, John A. Sayer, Neil Sebire, Haresh Selvaskandan, Sapna Shah, Asheesh Sharma, Edward J. Sharples, Neil Sheerin, Harish Shetty, Rukshana Shroff, Roslyn Simms, Manish Sinha, Smeeta Sinha, Kerry Smith, Lara Smith, Shalabh Srivastava, Retha Steenkamp, Ian Stott, Katerina Stroud, Pauline Swift, Justyna Szklarzewicz, Fred Tam, Kay Tan, Robert Taylor, Marc Tischkowitz, Kay Thomas, Yincent Tse, Alison Turnbull, A. Neil Turner, Kay Tyerman, Miranda Usher, Gopalakrishnan Venkat-Raman, Alycon Walker, Stephen B. Walsh, Aoife Waters, Angela Watt, Phil Webster, Ashutosh Wechalekar, Gavin Iain Welsh, Nicol West, David Wheeler, Kate Wiles, Lisa Willcocks, Angharad Williams, Emma Williams, Karen Williams, Deborah H. Wilson, Patricia D. Wilson, Paul Winyard, Edwin Wong, Katie Wong, Grahame Wood, Emma Woodward, Len Woodward, Adrian Woolf, David Wright

**Affiliations:** 1 St George’s University Hospitals NHS Foundation Trust, UK; 2 Evelina London Children's Hospital, UK; 3 David Evans Medical Research Centre, Nottingham University Hospital NHS Trust, UK; 4 Guy’s and St Thomas NHS foundation Trust, UK; 5 Ysbyty Gwynedd, Betsi Cadwaladr University Health Board, UK; 6 Imperial College Healthcare NHS Trust, UK; 7 James Paget University Hospital NHS Foundation Trust, UK; 8 Heart of England NHS Foundation Trust, Birmingham, UK; 9 Mid and South Essex NHS Foundation Trust, UK; 10 Royal Berkshire NHS Foundation Trust, UK; 11 Bradford Teaching Hospitals NHS Foundation Trust, UK; 12 Royal United Hospital Bath NHS Trust, UK; 13 Freeman Hospital, Newcastle Upon Tyne, UK; 14 Birmingham Women's and Children's NHS Foundation Trust, UK; 15 Manchester University NHS Foundation Trust, UK; 16 Royal Devon University Healthcare NHS Foundation Trust, UK; 17 Medizinische Genetik Mainz, Mainz, Germany; 18 Department of Medicine, Faculty of Medicine, Medical Center-University of Freiburg, Freiburg, Germany; 19 Hull University Teaching Hospitals NHS Trust, UK; 20 Exeter Kidney Unit, Royal Devon University Healthcare NHS Foundation Trust, UK; 21 Gloucestershire Hospitals NHS Foundation Trust, UK; 22 Oxford University Hospitals NHS Foundation Trust, UK; 23 UK Kidney Association, UK; 24 Countess of Chester NHS Foundation Trust, UK; 25 National Renal Complement Therapeutics Centre, Newcastle upon Tyne Hospitals NHS Foundation Trust, Newcastle upon Tyne, UK; 26 University Hospitals of Leicester NHS Trust, UK; 27 Barts Health NHS Trust, London, UK; 28 King's College Hospital NHS Foundation Trust, UK; 29 East Suffolk and North Essex NHS Foundation Trust, UK; 30 Ninewells Hospital and Medical School, Dundee, UK; 31 North West Anglia NHS Foundation Trust, UK; 32 Northern Health and Social Care Trust and Northern Ireland Clinical Research Network; 33 West Suffolk NHS Foundation Trust, UK; 34 Morriston Hospital, Swansea Bay Health Board, UK; 35 Lister Hospital, East and North Hertfordshire NHS Trust, UK; 36 Dartford and Gravesham NHS Trust, UK; 37 Nottingham Children’s Hospital, UK; 38 Salford Royal Hospital, Northern Care Alliance NHS Foundation Trust, Salford, UK; 39 University of Manchester, UK; 40 King’s College London, UK; 41 Nottingham University Hospitals NHS trust, UK; 42 Epsom and St Helier University Hospitals NHS Trust, UK; 43 University of Bristol Medical School, Bristol, UK; 44 Royal Stoke University Hospital, UK; 45 Manchester Royal Infirmary, UK; 46 Centre for Inflammatory Disease, Imperial College London, UK; 47 Nephrotic Syndrome Trust' (NeST), UK; 48 North Cumbria Integrated Care NHS Foundation Trust, UK; 49 Colchester General Hospital, UK; 50 Tameside and Glossop Integrated Care NHS Foundation Trust, UK; 51 Wye Valley NHS Trust, UK; 52 BHF Centre for Cardiovascular Science, The Queen's Medical Research Institute, University of Edinburgh, UK; 53 Lancashire Teaching Hospital, UK; 54 School of Medicine, University of Nottingham, UK; 55 Leeds Teaching Hospitals NHS Trust, UK; 56 University of Birmingham, UK; 57 Liverpool University Hospitals Foundation NHS Trust, UK; 58 Salford Royal NHS Foundation Trust, UK; 59 Department of Clinical Genetics, Guy’s and St Thomas’ NHS Foundation Trust, UK; 60 Centre for Health and Related Research, School of Population Health, University of Sheffield, UK; 61 University College London Department of Renal Medicine, Royal Free Hospital, UK; 62 SW Thames Renal Unit, Epsom and St Helier University Hospitals NHS Trust, UK; 63 Alport UK, UK; 64 Queen Elizabeth University Hospital, Glasgow, UK; 65 College of Medicine and Health, University of Exeter, UK; 66 Divison of Population Health, University of Sheffield, UK; 67 University of Wolverhampton, UK; 68 Patient Representative, UK; 69 Institute of Liver Studies, King’s College London, UK; 70 Sheffield Kidney Institute, Sheffield Teaching Hospitals NHS Foundation Trust, UK; 71 Royal Free Hospital, UK; 72 Manchester Institute of Nephrology and Transplantation, Manchester Royal Infirmary, UK; 73 PKD Charity, UK; 74 University Hospital Southampton NHS Foundation Trust, UK; 75 Children's Kidney centre, University Hospital of Wales, UK; 76 Travere Therapeutics, UK; 77 University Hospitals Coventry and Warwickshire NHS Trust, UK; 78 County Durham & Darlington NHS Foundation Trust, UK; 79 University of Leicester, UK; 80 Wirral University Teaching Hospital NHS Foundation Trust, UK; 81 University Hospitals Birmingham NHS Foundation Trust, UK; 82 Department of Medicine, University of Cambridge, UK; 83 North Bristol NHS Trust, UK; 84 Alder Hey Childrens NHS Foundation Trust, UK; 85 York & Scarborough Teaching Hospitals NHS Foundation Trust, UK; 86 Norfolk and Norwich University Hospitals NHS Trust, UK; 87 Royal Manchester Children’s Hospital, Manchester, UK; 88 PTEN UK and Ireland Patient Group; 89 HNF1B Support Group, UK; 90 School of Medicine, Keele University, UK; 91 Wellcome Centre for Cell-Matrix Research, University of Manchester, UK; 92 Research Department of Pathology, University College London, UK; 93 HLRCC Foundation, UK; 94 Cambridge Institute of Therapeutic Immunology and Infectious Disease, Cambridge, UK; 95 Newcastle University, UK; 96 United Lincolnshire Hospitals NHS Trust, UK; 97 Department of Medical Genetics, University of Cambridge, UK; 98 University Hospitals of Derby and Burton NHS Foundation Trust, UK; 99 Retroperitoneal Fibrosis (RF) Group, UK; 100 National Institute of Health and Care Research Leeds Biomedical Research Centre, Leeds Teaching Hospitals NHS Trust, UK; 101 School of Medicine, University of Leeds, UK; 102 University of Liverpool, UK; 103 Newcastle Upon Tyne Hospitals NHS Foundation Trust, UK; 104 Research Department of Structural and Molecular Biology, University College London, UK; 105 Division of Cardiology, Cliniques Universitaires Saint-Luc, Belgium; 106 Pole of Cardiovascular Research, Institut de Recherche Expérimentale et Clinique, Université Catholique de Louvain, Brussels, Belgium; 107 Cambridge University Hospitals NHS Foundation Trust, UK; 108 East and North Hertfordshire NHS Trust, UK; 109 Department of Immunology and Inflammation, Faculty of Medicine, Imperial College London, UK; 110 Shrewsbury and Telford Hospital NHS Trust, UK; 111 National Institute of Health and Care Research Great Ormond Street Hospital Biomedical Research Centre, UK; 112 UCL Great Ormond Street Institute of Child Health, UK; 113 Northern Care Alliance NHS Foundation Trust, UK; 114 South Tyneside and Sunderland NHS Foundation Trust, UK; 115 Doncaster and Bassetlaw Teaching Hospitals, UK; 116 New Cross Hospital, Wolverhampton, UK; 117 Wellcome Centre for Mitochondrial Research, Translational & Clinical Research Institute, Faculty of Medical Sciences, Newcastle University, UK; 118 Great North Children's Hospital, Newcastle Upon Tyne, UK; 119 University of Edinburgh, UK; 120 Calderdale & Huddersfield Foundation Trust, UK; 121 Royal Surrey County Hospital, Guildford, UK; 122 South Tees Hospitals NHS Foundation Trust, UK; 123 University College Cork, Ireland; 124 National Amyloidosis Centre, University College London, UK; 125 Great Western Hospital, Swindon, UK; 126 North Tees and Hartlepool NHS Foundation Trust, UK; 127 University College London, UK; 128 aHUS Alliance, UK; 129 School of Biological Sciences, University of Manchester, UK; 1 National Registry of Rare Kidney Diseases, Bristol, UK; 2 Department of Renal Medicine, University College London, UK; 3 UK Renal Registry, Bristol, UK; 4 St George’s, University of London, UK; 5 University of Leicester, UK; 6 University of Exeter, UK; 7 University of Bristol, UK; 8 University of Manchester, UK; 9 Guy’s and St Thomas’ NHS Foundation Trust, UK; 10 Cardiff University, UK; 11 University of Nottingham, UK; 12 Great North Children’s Hospital, Newcastle Upon Tyne, UK; 13 University of Cambridge, UK; 14 National Renal Complement Therapeutics Centre (NRCTC), Newcastle upon Tyne Hospitals NHS Foundation Trust, UK; 15 Complement Therapeutics Research Group, Translational and Clinical Research Institute, Newcastle University, UK; 16 Birmingham Women’s and Children’s NHS Foundation Trust, UK; 17 Royal Free London NHS Foundation Trust, UK; 18 University Hospitals Birmingham NHS Foundation Trust, UK; 19 Translational and Clinical Research Institute, Newcastle University, UK; 20 Department of Infection, Academic Unit of Nephrology, Immunity and Cardiovascular Disease, University of Sheffield, UK; 21 Northern Care Alliance NHS Foundation Trust, UK; 22 South Tyneside and Sunderland NHS Foundation Trust, UK; 23 Department of Immunology and Inflammation Centre for Inflammatory Disease, Imperial College London, UK; 24 Edinburgh University, UK; 25 Great Ormond Street Hospital for Children NHS Foundation Trust, London, UK; 26 Pfizer, Inc, New York, USA; 27 London School of Hygiene and Tropical Medicine, London, UK; 28 King’s College London and King’s Health Partners, London, UK

**Keywords:** ethnicity, RaDaR, rare kidney disease registry, rare kidney diseases, social deprivation

## Abstract

**Introduction:**

The National Registry of Rare Kidney Diseases (RaDaR) collects data from people living with rare kidney diseases across the UK, and is the world’s largest, rare kidney disease registry. We present the clinical demographics and renal function of 25,880 prevalent patients and sought evidence of bias in recruitment to RaDaR.

**Methods:**

RaDaR is linked with the UK Renal Registry (UKRR, with which all UK patients receiving kidney replacement therapy [KRT] are registered). We assessed ethnicity and socioeconomic status in the following: (i) prevalent RaDaR patients receiving KRT compared with patients with eligible rare disease diagnoses receiving KRT in the UKRR, (ii) patients recruited to RaDaR compared with all eligible unrecruited patients at 2 renal centers, and (iii) the age-stratified ethnicity distribution of RaDaR patients with autosomal dominant polycystic kidney disease (ADPKD) was compared to that of the English census.

**Results:**

We found evidence of disparities in ethnicity and social deprivation in recruitment to RaDaR; however, these were not consistent across comparisons. Compared with either adults recruited to RaDaR or the English population, children recruited to RaDaR were more likely to be of Asian ethnicity (17.3% vs. 7.5%, *P*-value < 0.0001) and live in more socially deprived areas (30.3% vs. 17.3% in the most deprived Index of Multiple Deprivation (IMD) quintile, *P*-value < 0.0001).

**Conclusion:**

We observed no evidence of systematic biases in recruitment of patients into RaDaR; however, the data provide empirical evidence of negative economic and social consequences (across all ethnicities) experienced by families with children affected by rare kidney diseases.

## Graphical abstract

**Figure:**
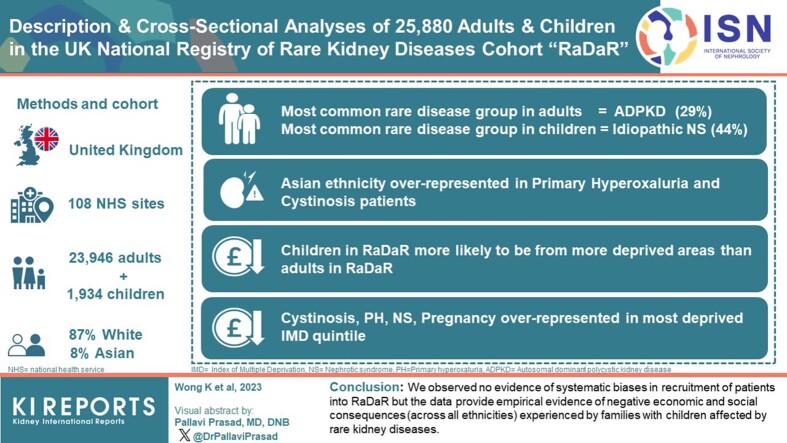

A rare disease is defined in Europe as a condition affecting less than 1 in 2000 people,^1^ and in the USA as affecting fewer than 200,000 individuals in the country.^2^ Rare kidney diseases make a significant contribution to the burden of kidney disease in the UK and globally. At least 25% of adults and over 50% of children receiving KRT have a rare disease^3^ with “glomerulonephritis” being the single commonest category of primary renal disease among UK patients receiving KRT.^4^

Small patient numbers can result in challenges in clinical management and research in rare diseases. Lack of clinical experience, even in large academic centers can lead to delays or errors in diagnosis and treatment of rare diseases; and low disease incidence alongside underdiagnosis can make identification of patients eligible for clinical trials and observational studies challenging. Adequate patient numbers for meaningful analysis may only be achieved through collaboration between multiple large renal centers, associated with considerable administrative burden.^5^

Kidney disorders can cause multisystem dysfunction and may require complex multidisciplinary care at different specialist centers. Advances in KRT have led to people with rare kidney disorders surviving for decades with kidney failure (KF),^1^ so the requirement for long-term follow-up data is paramount. For children with rare kidney diseases, life-time follow-up across different specialist pediatric and adult health care centers across different regions may be needed, leading to fragmentation of records across multiple databases, systems, and health care providers, which is challenging to access for research. Rare kidney disorders are therefore frequently poorly characterized, lacking published data on the prevalence rates, determinants, distribution, and long-term outcomes of these diseases.

RaDaR, set-up in 2010 by the UK Kidney Association with funding from the Medical Research Council, Kidney Care UK, and Kidney Research UK, was designed to address these challenges by collecting longitudinal data (without biological specimens) for UK adults and children with rare kidney diseases. Uniquely embedded with the publicly funded National Health Service (NHS) to which all UK residents have free access, RaDaR is hosted by the UKRR and has UK-wide ethical approval as a research registry, enabling automated collection of retrospective and prospective data for patients across multiple regions. The aims of RaDaR include the following: (i) to better understand the natural history of rare kidney diseases (as we recently reported^6^), (ii) to assess long-term effects of therapies, (iii) to identify cohorts eligible for clinical research, and (iv) provide infrastructure for individual rare disease studies and subregistries.

To our knowledge, RaDaR is the largest rare kidney disease registry worldwide. Here we describe the set-up and data flow into RaDaR, and present cross-sectional analyses of 25,880 prevalent patients and minimum point prevalence estimates for 21 rare kidney diseases in the UK.

## Methods

### Structure of RaDaR

**Figure 1:**
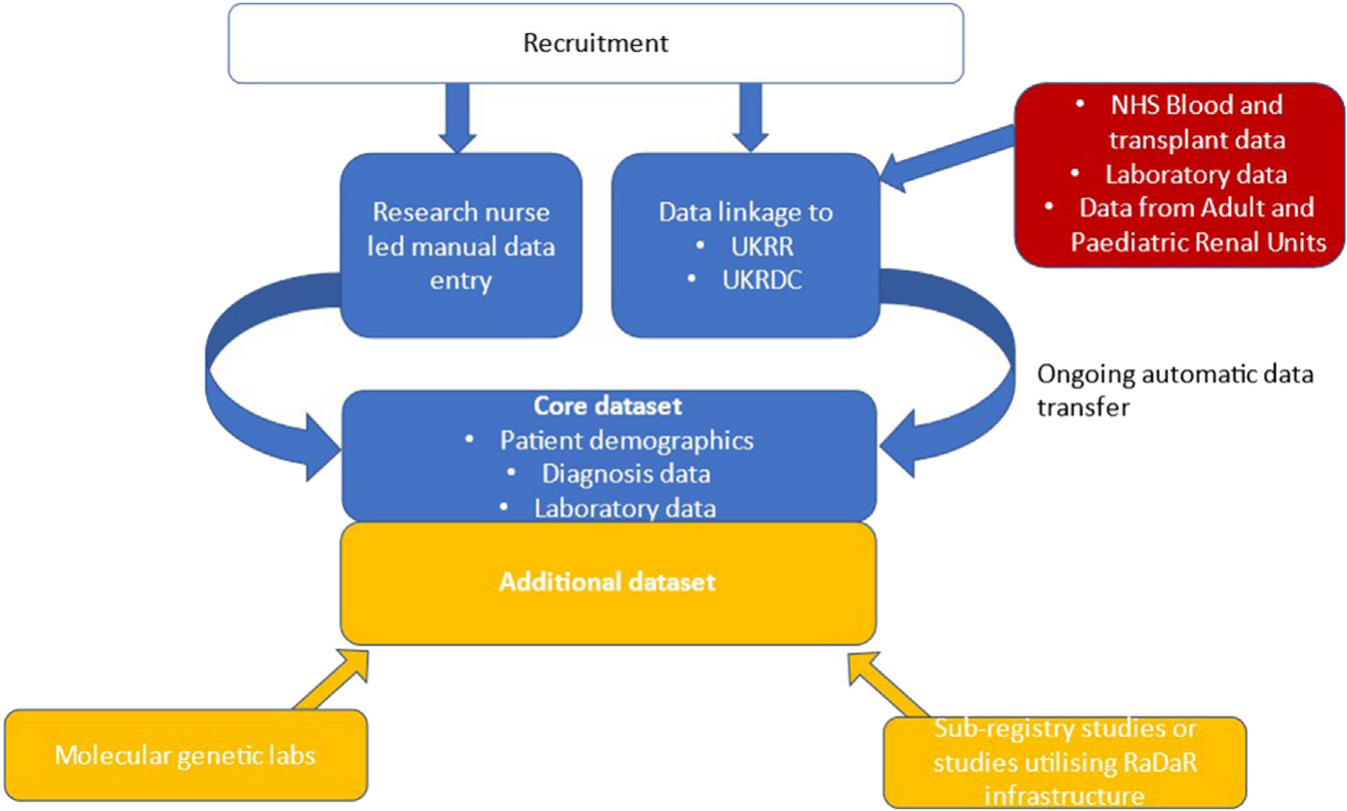
Recruitment and data flow to RaDaR. RaDaR, National Registry of Rare Kidney Diseases; UKRDC, UK Renal Data Collaboration; UKRR, UK Renal Registry.

Recruitment and data transfer are summarized in Figure 1. All participants sign a consent form agreeing to storage and analysis of their clinical data; in most cases, to linking their data to that held in other databases, studies, and registries; and to be contacted for future research studies they may be eligible for. All data are held centrally in a Structured Query Language database at the UKRR. National Institute for Health and Care Research infrastructure and research nurse supports NHS sites to manually enter a minimal set of mandatory fields at the time of recruitment to RaDaR; this infrastructure has also supported other national research programs such as the RECOVERY^7^ trial. Manual data entry is automatically checked using defined ranges to identify implausible data.

Data on all patients recruited to RaDaR were extracted on July 25, 2022.

### Rare Disease Groups (RDGs)

Participants are recruited into 29 RDGs, which may comprise a single disease or groups of renal diagnoses. Full eligibility criteria for each RDG are shown in Supplementary Table S15 and available online at https://ukkidney.org/sites/renal.org/files/radar/Inclusion-Exclusion_april_2021_v22.pdf.

Data for the following RDGs with >85 patients are presented: ADPKD, autosomal dominant tubulointerstitial kidney disease, atypical hemolytic uremic syndrome, Alport syndrome, autosomal recessive polycystic kidney disease and nephronophthisis, HNF1B mutations, IgA nephropathy, idiopathic nephrotic syndrome, membranous nephropathy, monoclonal gammopathy of renal significance, membranoproliferative glomerulonephritis and C3 glomerulopathy; pregnancy, inherited renal cancers; retroperitoneal fibrosis, Shiga toxin/verotoxin-producing Escherichia coli-associated hemolytic uremic syndrome, tuberous sclerosis complex, vasculitis, cystinosis, cystinuria, primary hyperoxaluria, and tubulopathies. Results for males and females with X-linked Alport syndrome, and individuals with thin basement membrane nephropathy are presented separately because these conditions are usually X-linked or autosomal respectively. Patients with autosomal recessive Alport syndrome have been excluded due to small sample size. Results for the vasculitis RDG are presented stratified into antineutrophil cytoplasmic antibody-associated vasculitis, antiglomerular basement membrane disease, and other vasculitis (including large vessel and IgA vasculitis). The idiopathic nephrotic syndrome cohort is presented stratified into patients with a diagnosis of either steroid sensitive nephrotic syndrome or minimal change disease (SSNS/MCD); steroid resistant nephrotic syndrome, congenital nephrotic syndrome, or focal segmental glomerulosclerosis (SRNS/FSGS); or idiopathic nephrotic syndrome-unspecified (patients without a confirmed diagnosis of SSNS/MCD or SRNS/FSGS).

### Data Linkage

RaDaR is linked with the UKRR for data on KRT initiation and death, which receives data from NHS Blood and Transplant for transplantation events. Rates of KRT data received from the UKRR are correct as of 1st January 2022. Routine laboratory data are extracted via automated feed either directly from renal unit information technology systems or via the UK Renal Data Collaboration.

### Demographic Data

Self-reported ethnicity (Office of National Statistics census categories^8^) is entered manually by a research nurse at the time of recruitment or populated from existing clinical data provided by the UK Renal Data Collaboration. Sex is reported according to UK Renal Data Collaboration record. Postcodes (zip codes) were used to derive IMD scores as an area level measure of socioeconomic status. IMD is a measure of relative deprivation for small areas within a country from most deprived to least deprived (1 = most deprived). Each country in the UK (England,^9^ Wales,^10^ Scotland^11^ and Northern Ireland^12^) has an IMD. These can then be categorized into country-specific quintiles within each country (quintile 1 = most deprived, quintile 5 = least deprived).

### Renal Function

For patients not receiving KRT, estimated glomerular filtration rate was calculated using chronic kidney disease (CKD)-epidemiology collaboration creatinine equation without race adjustment (2009)^13^ or Schwartz equation for patients aged ≤16 years.

### Missing Data

Available data were presented for each variable and patterns for missing data explored and proportions presented in Supplementary Tables S1 to S3.

### Small Number Suppression

Where a risk of reidentification of participants were identified, groups with small numbers were aggregated into larger groups and tabulated data were structured not to report fewer than 6 participants per cell where possible. Where cells contained ≤6 counts, this cell was suppressed. To avoid possibility of calculation of suppressed counts, corresponding cells were rounded to the nearest 5, in accordance with NHS Digital guidance.^14^

### Minimum Point Prevalence Estimates

UK wide RaDaR point prevalence estimates were calculated using patient numbers for each RDG, and stratified by sex, using Office of National Statistics UK population data^15^ and were presented per 100,000 population. Prevalence estimates were also calculated for each RDG for each UK Health Board, and maximum estimated rate for each RDG were presented. Due to the nature of recruitment to RaDaR, which requires informed consent from participants, these estimated UK-wide rates could underestimate the true rate of rare kidney diseases but could be interpreted as minimum possible rates.

### Statistical Analyses

Baseline characteristics were presented as frequencies (%) for categorical data and medians (interquartile range) for continuous data. Chi-square or Fisher exact tests were used to compare categorical variables. Statistical analyses were performed using STATA Release 17 (StataCorp LLC, College Station, TX) and SAS version 9.4 (SAS Institute Inc., Cary, NC).

### Ethnicity and Social Deprivation Comparisons

For each RDG, the proportion of patients in each ethnic group and each IMD quintile was compared to the overall RaDaR proportion, excluding that RDG. Because ethnic and IMD quintile distributions differed between adult and pediatric populations, these analyses were performed stratified by age category.

### Recruitment Comparisons

Primary renal diagnosis is recorded in the UKRR using European Renal Association-European Dialysis and Transplant Association codes^16^ and in UK renal center information systems either using primary renal diagnosis codes or free-text. Primary renal diagnosis codes and a list of search terms specific to each RDG were decided with agreement from RDG leads (clinicians with expertise in that rare kidney disease), to generate an overall list of primary renal diagnosis codes and keywords for RaDaR diagnoses (Supplementary Table S4).

To assess whether some RDGs had recruited a greater proportion of the total eligible patients in the UK than others, living patients in the UKRR receiving KRT who were eligible for RaDaR based on EDTA codes were stratified into their potential RDGs, and the percentage in each RDG compared to the percentage of living patients in RaDaR receiving KRT.

To assess whether there has been ethnic or socioeconomic status recruitment bias, 3 methods were used as follows: (i) ethnicity and socioeconomic status of all prevalent RaDaR patients who had reached KF were compared with patients with a rare kidney diagnosis in the UKRR, (ii) patients recruited to RaDaR from 2 large UK renal centers were compared with all unrecruited patients with a RaDaR eligible diagnosis at those centers, (iii) the age-stratified ethnicity distribution of England according to the 2011 UK census was compared with the ethnicity of prevalent English RaDaR patients with ADPKD. Patients from Scotland, Wales, and Northern Ireland were excluded from comparisons with the 2011 UK census due to lack of available data regarding age-stratified ethnicity from the Office of National Statistics for those nations. English nationality was determined by a home address with an English postcode. More detailed information about these comparisons is presented in the Supplementary Methods.

The RaDaR database has approval for research studies from the NHS South-West-Central Bristol Research Ethics Committee (19/SW/0173). This report was written with reference to the Strengthening the Reporting of Observation Studies in Epidemiology (STROBE) statement.^17^ Additional methods can be found in the Supplementary Methods.^S1, S2^

## Results

As of July 2022, RaDaR recruited patients from 108 NHS sites (96 adult and 12 pediatric) across England (*n* = 91), Scotland (*n* = 9), Wales (*n* = 3), and Northern Ireland (*n* = 5). Most patients have been recruited from English renal units (*n* = 23,776, 92%). Recruitment at each center is shown in Supplementary Table S14. Data from RDGs with ≥85 patients recruited are presented.

### Clinical demographics of the RaDaR Patient Population

Clinical characteristics of 25,880 prevalent patients in RaDaR on July 25, 2022, are presented in Table 1; 2957 patients (11%) are now deceased. One hundred twenty-five patients (0.5%) have more than 1 diagnosis recorded; the majority of these are in the pregnancy RDG (115/125, 92%). A total of 5260 renal pathology reports are available for 4184 participants, mostly in the larger glomerular disease RDGs.

**Table 1:** Patient demographics of individuals recruited to RaDaR, stratified by rare disease group and current age^a^

Rare disease group	Pediatric	Adults	Pediatric		Adults		Median current age				Deceased
			males	females	males	females	pediatric		adult		
	*n* (%)	*n* (%)	*n* (%)	*n* (%)	*n* (%)	*n* (%)	median	(IQR)	median	(IQR)	All
All RaDaR	1934 (7.5)	23946 (92.5)	1072 (55.4)	862 (44.6)	12811 (53.5)	11135 (46.5)	12	(9.3–15.0)	56	(41.5–67.9)	2956
Monogenic or congenital conditions											
ADPKD	119 (1.7)	6993 (98.3)	59 (49.6)	60 (50.4)	3302 (47.2)	3691 (52.8)	13	(11.2–15.4)	55	(44.1– 65.0)	685
ADTKD	≤6 (NR^a^)	190 (>97.0)	0 (0.0)	3 (100.0)	80 (42.6)	108 (57.4)	17	(15.7–17.2)	54	(43.3– 63.9)	24
X-linked Alport- female	46 (15.5)	250 (84.5)		46 (100.0)		250 (100.0)	11	(8.7–14.6)	46	(33.3– 59.3)	13
X-linked Alport- male	53 (13.8)	332 (86.2)	53 (100.0)		332 (100.0)		12	(10.0–14.9)	43	(29.4– 56.4)	27
TBMN	16 (9.9)	146 (90.1)	≤6 (NR)	10 (>63.0)	48 (32.9)	98 (67.1)	12	(9.5–16.1)	47	(32.0– 58.6)	3
ARPKD/NPHP	71 (33.0)	144 (67.0)	40 (56.3)	31 (43.7)	64 (44.4)	80 (55.6)	12	(9.2–14.5)	39	(26.5– 54.8)	18
Cystinosis	54 (37.5)	90 (62.5)	26 (48.1)	28 (51.9)	43 (47.8)	47 (52.2)	13	(8.9–15.7)	29	(23.6– 36.4)	9
Cystinuria	28 (6.1)	432 (93.9)	19 (67.9)	9 (32.1)	222 (51.4)	210 (48.6)	11	(7.2–14.5)	49	(34.4– 62.1)	13
Hyperoxaluria	25 (21.7)	90 (78.3)	12 (48.0)	13 (52.0)	59 (65.6)	31 (34.4)	11	(9.0–13.3)	36	(26.4– 52.1)	9
HNF1B mutations	31 (36.5)	54 (63.5)	19 (61.3)	12 (38.7)	28 (51.9)	26 (48.1)	9	(6.8–14.2)	39	(23.7–51.1)	1
Renal cancer inherited	10 (8.8)	103 (91.2)	≤6 (NR)	≤6 (NR)	39 (37.9)	64 (62.1)	10	(8.4–12.5)	54	(35.4–60.8)	1
Tubulopathies	76 (18.7)	331 (81.3)	54 (71.1)	22 (28.9)	155 (46.8)	176 (53.2)	12	(8.1–15.9)	40	(29.5–56.0)	12
Tuberous sclerosis complex	43 (17.8)	199 (82.2)	16 (37.2)	27 (62.8)	81 (40.7)	118 (59.3)	11	(8.9–15.0)	39	(29.1–51.8)	7
Mostly nonmonogenic or acquired conditions											
aHUS	89 (32.2)	187 (67.8)	43 (48.3)	46 (51.7)	85 (45.5)	102 (54.5)	10	(7.0–13.9)	42	(32.7–56.4)	17
SSNS/MCD	525 (31.8)	1127 (68.2)	329 (62.7)	196 (37.3)	638 (56.6)	489 (43.4)	12	(9.3–14.2)	47	(31.5–63.6)	53
SRNS/FSGS	256 (18.2)	1154 (81.8)	137 (53.5)	119 (46.5)	650 (56.3)	504 (43.7)	13	(9.5–15.5)	49	(30.3–63.8)	126
INS-unspecified	63 (7.4)	792 (92.6)	38 (60.3)	25 (39.7)	449 (56.7)	343 (43.3)	12	(7.7–14.1)	55	(40.9–67.6)	120
IgA nephropathy	40 (1.1)	3756 (98.9)	25 (62.5)	15 (37.5)	2610 (69.5)	1146 (30.5)	14	(12.1–16.4)	53	(42.2–63.5)	351
Membranous nephropathy	≤6 (NR)	2050 (>99.0)	≤6 (NR)	≤6 (NR)	1358 (66.2)	692 (33.8)	14	(13.3–15.1)	66	(55.5–74.6)	384
MGRS	0 (0.0)	144 (100.0)	0 (0.0)	0 (0.0)	74 (51.4)	70 (48.6)			67	(56.1–76.3)	37
MPGN/C3GN	63 (6.8)	869 (93.2)	32 (50.8)	31 (49.2)	454 (52.2)	415 (47.8)	15	(12.5–16.6)	54	(35.3–66.1)	157
Pregnancy	≤6 (NR)	680 (>99.0)		1 (100.0)		681 (100.0)	16	(16.1–16.1)	37	(33.1–42.0)	10
Retroperitoneal fibrosis	0 (0.0)	111 (100.0)	0 (0.0)		72 (64.9)	39 (35.1)			67	(58.5–74.2)	31
STEC HUS	110 (65.9)	57 (34.1)	57 (51.8)	53 (48.2)	24 (42.1)	33 (57.9)	12	(8.7–14.6)	23	(20.1–33.2)	3
ANCA-associated vasculitis	7 (0.4)	1917 (99.6)	0 (0.0)	7 (100.0)	1005 (52.4)	912 (47.6)	14	(13.0–16.4)	70	(58.6–77.0)	451
Anti-GBM disease	≤6 (NR)	115 (>99.0)	0 (0.0)	1 (100.0)	57 (49.6)	58 (50.4)	15	(14.8–14.8)	62	(47.6–71.4)	21
Other vasculitides	200 (10.2)	1757 (89.8)	100 (50.0)	100 (50.0)	886 (50.4)	871 (49.6)	12	(9.7–15.0)	66	(51.0–75.4)	374

ADPKD, autosomal dominant polycystic kidney disease; ADTKD, autosomal dominant tubulointerstitial kidney disease; aHUS, atypical hemolytic uremic syndrome; ANCA, antineutrophil cytoplasmic antibody; ARPKD/NPHP, autosomal recessive polycystic kidney disease and nephronophthisis; C3GN, C3 glomerulopathy; FSGS, focal segmental glomerulosclerosis; GBM, glomerular basement membrane; INS, idiopathic nephrotic syndrome; IQR, interquartile range; MCD, minimal change disease; MGRS, monoclonal gammopathy of renal significance; MPGN, membranoproliferative glomerulonephritis; NR, not reported; RaDaR, National Registry of Rare Kidney Diseases; SRNS, steroid resistant nephrotic syndrome; SSNS, steroid sensitive nephrotic syndrome; STEC HUS, Shiga toxin/verotoxin-producing Escherichia coli-associated hemolytic uremic syndrome; TBMN, thin basement membrane nephropathy.

a Prevalent patients on July 25, 2022; cells with fewer than 6 patients not reported due to risk of reidentification. Where a cell is not reported due to small numbers, corresponding cell values are rounded to the nearest 5. Individuals with 2 diagnoses are presented once for all RaDaR results, but subsequently included for each diagnosis. Row percentages are presented.

The largest RDGs by patient number are ADPKD (*n* = 7112), vasculitis (*n* = 3997), idiopathic nephrotic syndrome (*n* = 3917), and IgA nephropathy (*n* = 3796). Conditions not presented due to low numbers are as follows: adenine phosphoribosyltransferase deficiency (*n* = 9), BK nephropathy (*n* = 62), CKD due to genetic factors in people of African Ancestry (CKD-AFRICA) (*n* = 65), calciphylaxis (*n* = 59), Fabry disease (*n* = 47), fibromuscular dysplasia (*n* = 42), mitochondrial renal disease (*n* = 4), and pure red cell aplasia (*n* = 7).

Distribution of rare kidney diseases differed between patients currently aged ≤18 years (pediatric) and those aged >18 years old. Predominant rare kidney diseases in adults were ADPKD (*n* = 6993, 29%), vasculitis (16%; antineutrophil cytoplasmic antibody-associated vasculitis, *n* = 1917; antiglomerular basement membrane disease, *n* = 115; and other vasculitides, *n* = 1757), and IgA nephropathy (*n* = 3756, 16%). In children the largest RDGs were idiopathic nephrotic syndrome (44%; SSNS/MCD, *n* = 525; SRNS/FSGS, *n* = 256; and idiopathic nephrotic syndrome-unspecified, *n* = 63), vasculitis (11%; antineutrophil cytoplasmic antibody-associated vasculitis, *n* = 7; antiglomerular basement membrane disease, ≤6; and other vasculitides, *n* = 200), and Alport syndrome (6%; X-linked males, *n* = 53; X-linked females, *n* = 46; and autosomal dominant tubulointerstitial kidney disease, *n* = 16). The most frequent rare kidney diseases at the time of diagnosis in adults and children were the same.

Males were overrepresented among pediatric patients with cystinuria (68%), HNF1B mutations (61%), idiopathic nephrotic syndrome (60%), and tubulopathies (71%) (due to male-predominant Lowe syndrome); among adult patients with membranous nephropathy (66%), retroperitoneal fibrosis (65%), and primary hyperoxaluria (66%); and among both children and adults with IgA nephropathy (children 63%, adults 70%). Minimum UK point prevalence and maximum area density estimates per 100,000 population for each RDG are presented in Table 2.

**Table 2:** RaDaR point prevalence rates and maximum area density estimates per 100,000 population,^a^ stratified by rare disease group and sex

Rare disease group	RaDaR point prevalence estimates	RaDaR maximum area density estimates	RaDaR point prevalence estimates	
			males	females
All RDGs	35.90 (35.46–36.34)	65.24 (58.65–72.56)	38.56 (37.92–39.21)	33.24 (32.65–33.84)
ADPKD	9.87 (9.64–10.10)	22.74 (19.22–26.91)	9.34 (9.03–9.66)	10.40 (10.07–10.73)
ADTKD	0.26 (0.23–0.30)	1.05 (0.74–1.47)	0.22 (0.18–0.28)	0.31 (0.26–0.37)
X-linked Alport- female	0.41 (0.37–0.46)	3.75 (0.53–26.65)		0.82 (0.73–0.92)
X-linked Alport- male	0.53 (0.48–0.59)	2.59 (0.83–8.02)	1.07 (0.97–1.18)	
TBMN	0.22 (0.19–0.26)	1.61 (1.04–2.50)	0.15 (0.11–0.20)	0.30 (0.25–0.36)
ARPKD/NPHP	0.30 (0.26–0.34)	0.93 (0.30–2.87)	0.29 (0.24–0.35)	0.31 (0.26–0.37)
Cystinosis	0.20 (0.17–0.23)	3.75 (0.53–26.65)	0.19 (0.15–0.24)	0.21 (0.17–0.26)
Cystinuria	0.64 (0.58–0.70)	2.17 (1.26–3.74)	0.67 (0.59–0.76)	0.61 (0.53–0.69)
Hyperoxaluria	0.16 (0.13–0.19)	0.96 (0.58–1.59)	0.20 (0.16–0.25)	0.12 (0.09–0.16)
HNF1B mutations	0.12 (0.10–0.15)	1.02 (0.59–1.76)	0.13 (0.10–0.17)	0.11 (0.08–0.14)
Renal cancer inherited	0.16 (0.13–0.19)	2.27 (1.51–3.41)	0.12 (0.09–0.17)	0.19 (0.15–0.24)
Tubulopathies	0.56 (0.51–0.62)	4.36 (0.61–30.95)	0.58 (0.51–0.66)	0.55 (0.48–0.63)
Tuberous sclerosis complex	0.34 (0.30–0.38)	0.87 (0.52–1.44)	0.27 (0.22–0.33)	0.40 (0.34–0.47)
aHUS	0.38 (0.34–0.43)	1.34 (0.34–5.37)	0.35 (0.30–0.42)	0.41 (0.35–0.48)
SSNS/MCD	2.29 (2.18–2.40)	5.44 (4.69–6.32)	2.68 (2.52–2.86)	1.90 (1.76–2.04)
SRNS/FSGS	1.96 (1.86–2.06)	4.46 (3.35–5.94)	2.18 (2.04–2.34)	1.73 (1.60–1.87)
INS-unspecified	1.18 (1.11–1.27)	3.85 (3.00–4.93)	1.35 (1.24–1.48)	1.02 (0.92–1.13)
IgA nephropathy	5.26 (5.10–5.43)	15.02 (5.64–40.01)	7.32 (7.04–7.60)	3.21 (3.03–3.40)
Membranous Nephropathy	2.85 (2.73–2.98)	5.98 (5.19–6.90)	3.78 (3.59–3.99)	1.92 (1.79–2.07)
MGRS	0.20 (0.17–0.23)	0.81 (0.47–1.39)	0.21 (0.16–0.26)	0.19 (0.15–0.24)
MPGN/C3GN	1.29 (1.21–1.38)	4.44 (0.62–31.50)	1.35 (1.23–1.47)	1.24 (1.13–1.36)
Pregnancy	0.94 (0.88–1.02)	7.45 (6.22–8.94)		1.89 (1.75–2.03)
Retroperitoneal fibrosis	0.15 (0.13–0.19)	0.96 (0.40–2.31)	0.20 (0.16–0.25)	0.11 (0.08–0.15)
STEC HUS	0.23 (0.20–0.27)	1.10 (0.57–2.11)	0.22 (0.18–0.28)	0.24 (0.19–0.29)
ANCA-associated vasculitis	2.67 (2.56–2.79)	7.51 (5.48–10.27)	2.80 (2.63–2.98)	2.55 (2.39–2.72)
Anti-GBM disease	0.16 (0.13–0.19)	0.96 (0.36–2.55)	0.16 (0.12–0.20)	0.16 (0.13–0.21)
Other Vasculitides	2.72 (2.60–2.84)	13.08 (11.23–15.25)	2.74 (2.58–2.92)	2.69 (2.53–2.87)

ADPKD, autosomal dominant polycystic kidney disease; ADTKD, autosomal dominant tubulointerstitial kidney disease; aHUS, atypical hemolytic uremic syndrome; ANCA, antineutrophil cytoplasmic antibody; ARPKD/NPHP, autosomal recessive polycystic kidney disease and nephronophthisis; C3GN, C3 glomerulopathy; FSGS, focal segmental glomerulosclerosis; GBM, glomerular basement membrane; INS, idiopathic nephrotic syndrome; MCD, minimal change disease; MGRS, monoclonal gammopathy of renal significance; MPGN, membranoproliferative glomerulonephritis; RaDaR, National Registry of Rare Kidney Diseases; RDG, rare disease group; SRNS, steroid resistant nephrotic syndrome; SSNS, steroid sensitive nephrotic syndrome; STEC HUS, Shiga toxin/verotoxin-producing Escherichia coli-associated hemolytic uremic syndrome; TBMN, thin basement membrane nephropathy.

a Estimates on July 25, 2022.

**Figure 2:**
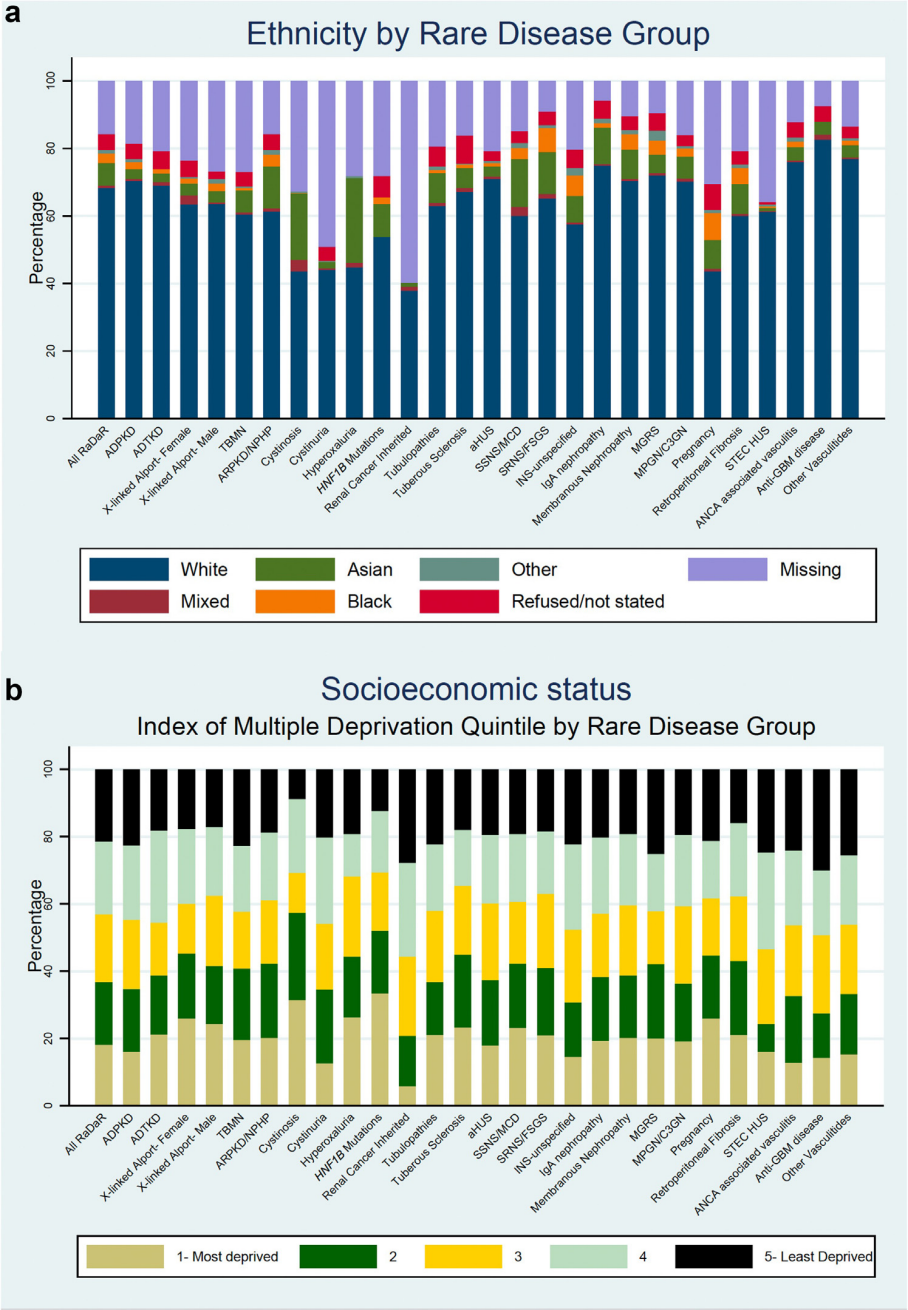
RaDaR patient (a) ethnicity and (b) socioeconomic status, stratified by rare disease group. ADPKD, autosomal dominant polycystic kidney disease; ADTKD, autosomal dominant tubulointerstitial kidney disease; aHUS, atypical hemolytic uremic syndrome; ANCA, antineutrophil cytoplasmic antibody; ARPKD/NPHP, autosomal recessive polycystic kidney disease and nephronophthisis; C3GN, C3 glomerulopathy; FSGS, focal segmental glomerulosclerosis; GBM, glomerular basement membrane; INS, idiopathic nephrotic syndrome; IQR, interquartile range; MCD, minimal change disease; MGRS, monoclonal gammopathy of renal significance; MPGN, membranoproliferative glomerulonephritis; NR, not reported; RaDaR, National Registry of Rare Kidney Diseases; SRNS, steroid resistant nephrotic syndrome; SSNS, steroid sensitive nephrotic syndrome; STEC HUS, Shiga toxin/verotoxin-producing Escherichia coli-associated hemolytic uremic syndrome; TBMN, thin basement membrane nephropathy.

Distribution of self-reported ethnicity and socioeconomic status for all RaDaR patients, stratified by RDG are presented in Figure 2. Excluding patients with missing data, 87% of RaDaR participants were White, 1% Mixed, 8% Asian, 3% Black and 1% Other ethnicities. The SSNS/MCD, SRNS/FSGS, IgA nephropathy, tubulopathies, cystinosis and primary hyperoxaluria RDGs, all had a significantly larger proportion of patients from Asian ethnic backgrounds than the total RaDaR population. This was particularly marked in the cystinosis and primary hyperoxaluria RDGs, where the proportions of patients from Asian backgrounds was 24% and 34%, respectively. Similar differences were observed when stratified by pediatric and adult populations (Supplementary Figures S1 and S2). Individuals recruited to the pregnancy RDG were also more likely to be from Asian (16%) or Black (11%) backgrounds. Children recruited to RaDaR from all 4 UK nations were more likely to be from Asian ethnic backgrounds when compared to adults recruited to RaDaR (17% vs. 8%, *P* < 0.0001, Supplementary Table 5). Similarly, Asian ethnic background was overrepresented among English children recruited to RaDaR when compared with children in the general English population (18% vs. 10%, *P*-value < 0.0001, Supplementary Table S6).

Of the patients recruited to RaDaR, 38% had monogenic disorders (disorders usually caused by the presence of 1 or 2 pathogenic variants in a single gene). Stratified by ethnicity, most monogenic disorders were diagnosed in White patients (90.3%) (Supplementary Table S7). Adults recruited to RaDaR were more likely to be diagnosed with monogenic disorders than children (39% vs. 30%, *P*-value < 0.0001, Supplementary Table S8). Only adults from White, Mixed, and Black backgrounds were more likely to be diagnosed with monogenic disorders compared to children; children from Asian or Other ethnic backgrounds were as likely to have a diagnosis of a monogenic disorder as adults (24% vs. 23%, *P*-value: 0.7 and 33% vs. 32%, *P*-value: 0.9, respectively).

Socioeconomic deprivation varied by RDG; patients diagnosed with cystinosis, primary hyperoxaluria, SSNS/MCD, SRNS/FSGS and pregnancy were more likely to be in the most deprived IMD quintile compared to the overall RaDaR population (11% pts, 14% pts, 8% pts, 5% pts, and 9% pts higher, respectively). There were similar differences in both adult and pediatric patients (Supplementary Figures S3 and S4). Individuals with nonmonogenic disorders were more likely to be in the most deprived IMD quintile compared to those with monogenic disorders (19.2% vs. 16.9%, *P*-value < 0.0001); however, this association was attenuated when stratified by ethnicity (Supplementary Table S9). Individuals with autosomal dominant conditions were less likely to be in the most deprived quintile compared to those with autosomal recessive, X-linked or nonmonogenic disorders (16% vs. 20% vs. 19%, respectively; *P*-value < 0.0001; Supplementary Table S10).

More pediatric patients were in the most deprived IMD quintile compared to adults recruited to RaDaR (30% vs. 17%, Supplementary Table S5), and compared to children in the general English population (Supplementary Table S6). Pediatric patients of White, Asian, and other ethnicities were all more likely to be in the most deprived IMD quintile compared to adults (25% vs. 16%, *P* < 0.0001; 54% vs. 31%, *P* < 0.0001; 50% vs. 25%, *P* = 0.02, respectively; Supplementary Table S11). Children (with nonmonogenic [30.2% vs. 18.2%, *P*-value < 0.0001] and monogenic [30.5% vs. 16.1%, *P*-value < 0.0001] disorders across all modes of inheritance) were more likely to be in the most deprived quintile compared to adults (Supplementary Tables S10 and S12).

### Renal Function of the RaDaR patient population

Many patients in RaDaR had reached KF (CKD stage G5 or KRT) (39%) (Table 3). This proportion varied by RDG; only 2% of patients with cystinuria had reached KF compared to 73% of male patients with Alport syndrome. Most pediatric patients had estimated glomerular filtration rate results >60 ml/min per 1.73 m^2^ (71% CKD stages G1–G2 vs. 32% of adult patients).

**Table 3:** Chronic kidney disease stage and median eGFR of RaDaR patients on January 1, 2022

Rare disease group	CKD Stage														Median eGFR^a^	
	G1		G2		G3a		G3b		G4		G5		RRT			
	*n*	(%)	*n*	(%)	*n*	(%)	*n*	(%)	*n*	(%)	*n*	(%)	*n*	(%)	(IQR)	
ADPKD	831	(13.4)	882	(14.3)	451	(7.3)	554	(9.0)	502	(8.1)	214	(3.5)	2753	(44.5)	60	(33.7–88.9)
ADTKD	16	(10.2)	11	(7.0)	13	(8.3)	14	(8.9)	15	(9.6)	3	(1.9)	85	(54.1)	47	(30.3–82.2)
X-linked Alport- female	76	(35.3)	27	(12.6)	13	(6.0)	11	(5.1)	6	(2.8)	6	(2.8)	76	(35.3)	93	(59.8–117.0)
X-linked Alport- male	43	(13.2)	17	(5.2)	6	(1.8)	12	(3.7)	8	(2.5)	3	(0.9)	237	(72.7)	88	(43.9–121.4)
TBMN	54	(41.2)	28	(21.4)	10	(7.6)	7	(5.3)	7	(5.3)	3	(2.3)	22	(16.8)	90	(61.4–108.7)
ARPKD/NPHP	22	(12.4)	18	(10.2)	9	(5.1)	12	(6.8)	18	(10.2)	6	(3.4)	92	(52.0)	53	(29.2–92.3)
Cystinosis	6	(4.9)	10	(8.2)	7	(5.7)	6	(4.9)	5	(4.1)	3	(2.5)	85	(69.7)	55	(35.3–78.3)
Cystinuria	119	(37.9)	133	(42.4)	34	(10.8)	13	(4.1)	5	(1.6)	3	(1.0)	7	(2.2)	81	(65.0–98.6)
Hyperoxaluria	19	(22.6)	21	(25.0)	4	(4.8)	5	(6.0)	1	(1.2)			34	(40.5)	83	(64.5–96.8)
HNF1B mutations	7	(14.3)	8	(16.3)	9	(18.4)	4	(8.2)	8	(16.3)	1	(2.0)	12	(24.5)	55	(31.3–79.3)
Tubulopathies	118	(53.4)	45	(20.4)	15	(6.8)	12	(5.4)	2	(0.9)	2	(0.9)	27	(12.2)	100	(72.8–116.9)
Tuberous sclerosis complex	70	(45.8)	31	(20.3)	14	(9.2)	11	(7.2)	4	(2.6)	3	(2.0)	20	(13.1)	91	(61.5–111.0)
aHUS	32	(16.0)	23	(11.5)	9	(4.5)	4	(2.0)	9	(4.5)	5	(2.5)	118	(59.0)	81	(47.5–108.3)
SSNS/MCD	672	(54.9)	304	(24.8)	91	(7.4)	51	(4.2)	18	(1.5)	4	(0.3)	84	(6.9)	97	(74.4–118.5)
SRNS/FSGS	315	(24.3)	180	(13.9)	87	(6.7)	66	(5.1)	67	(5.2)	33	(2.5)	547	(42.2)	81	(47.7–108.1)
INS-unspecified	163	(22.6)	143	(19.8)	44	(6.1)	43	(6.0)	37	(5.1)	10	(1.4)	282	(39.1)	78	(52.9–101.4)
IgA nephropathy	264	(7.6)	303	(8.7)	231	(6.7)	288	(8.3)	294	(8.5)	89	(2.6)	2000	(57.7)	49	(29.5–80.1)
Membranous Nephropathy	247	(14.3)	453	(26.2)	229	(13.2)	211	(12.2)	160	(9.2)	55	(3.2)	376	(21.7)	61	(39.4–83.4)
MGRS	1	(0.8)	14	(11.9)	12	(10.2)	17	(14.4)	8	(6.8)	8	(6.8)	58	(49.2)	42	(29.5–58.5)
MPGN/C3GN	135	(16.9)	81	(10.1)	43	(5.4)	49	(6.1)	35	(4.4)	16	(2.0)	441	(55.1)	72	(41.0–108.0)
Pregnancy	231	(37.1)	89	(14.3)	33	(5.3)	33	(5.3)	15	(2.4)	7	(1.1)	214	(34.4)	96	(67.2–115.8)
Retroperitoneal Fibrosis	9	(10.6)	25	(29.4)	20	(23.5)	12	(14.1)	4	(4.7)	4	(4.7)	11	(12.9)	57	(43.2–75.6)
STEC HUS	6	(13.6)	2	(4.5)	3	(6.8)	1	(2.3)	4	(9.1)			28	(63.6)	66	(34.7–123.9)
ANCA-associated vasculitis	111	(7.3)	308	(20.1)	278	(18.2)	252	(16.5)	200	(13.1)	35	(2.3)	346	(22.6)	50	(33.6–69.0)
Anti-GBM disease	5	(4.5)	1	(0.9)	3	(2.7)	4	(3.6)	3	(2.7)	1	(0.9)	95	(84.8)	47	(31.1–96.6)
Other vasculitides	156	(11.8)	260	(19.6)	184	(13.9)	183	(13.8)	131	(9.9)	30	(2.3)	380	(28.7)	56	(36.1–78.9]
Total	3673	(17.2)	3402	(16.0)	1849	(8.7)	1869	(8.8)	1565	(7.3)	544	(2.6)	8401	(39.4)		
Pediatric (Total)	471	(62.1)	68	(9.0)	15	(2.0)	11	(1.4)	12	(1.6)	5	(0.7)	177	(23.3)		
Adults (Total)	3202	(15.6)	3334	(16.2)	1834	(8.9)	1858	(9.0)	1553	(7.6)	539	(2.6)	8224	(40.0)		

ADPKD, autosomal dominant polycystic kidney disease; ADTKD, autosomal dominant tubulointerstitial kidney disease; aHUS, atypical hemolytic uremic syndrome; ANCA, antineutrophil cytoplasmic antibody; ARPKD/NPHP, autosomal recessive polycystic kidney disease and nephronophthisis; C3GN, C3 glomerulopathy; CKD, chronic kidney disease; eGFR, estimated glomerular filtration rate; FSGS, focal segmental glomerulosclerosis; GBM, glomerular basement membrane; INS, idiopathic nephrotic syndrome; IQR, interquartile range; KRT, kidney replacement therapy; MCD, minimal change disease; MGRS, monoclonal gammopathy of renal significance; MPGN, membranoproliferative glomerulonephritis; RaDaR, National Registry of Rare Kidney Diseases; RDG, rare disease group; SRNS, steroid resistant nephrotic syndrome; SSNS, steroid sensitive nephrotic syndrome; STEC HUS, Shiga toxin/verotoxin-producing *Escherichia coli*–associated hemolytic uremic syndrome; TBMN, thin basement membrane nephropathy.

a Median eGFR of patients not receiving KRT. Inherited renal cancers have been excluded from this table due to poor data completeness. For overall, pediatric, and adult totals individuals with 2 diagnoses included once. For RDG totals individuals with 2 diagnoses included for each diagnosis.

### Recruitment to RaDaR

**Figure 3:**
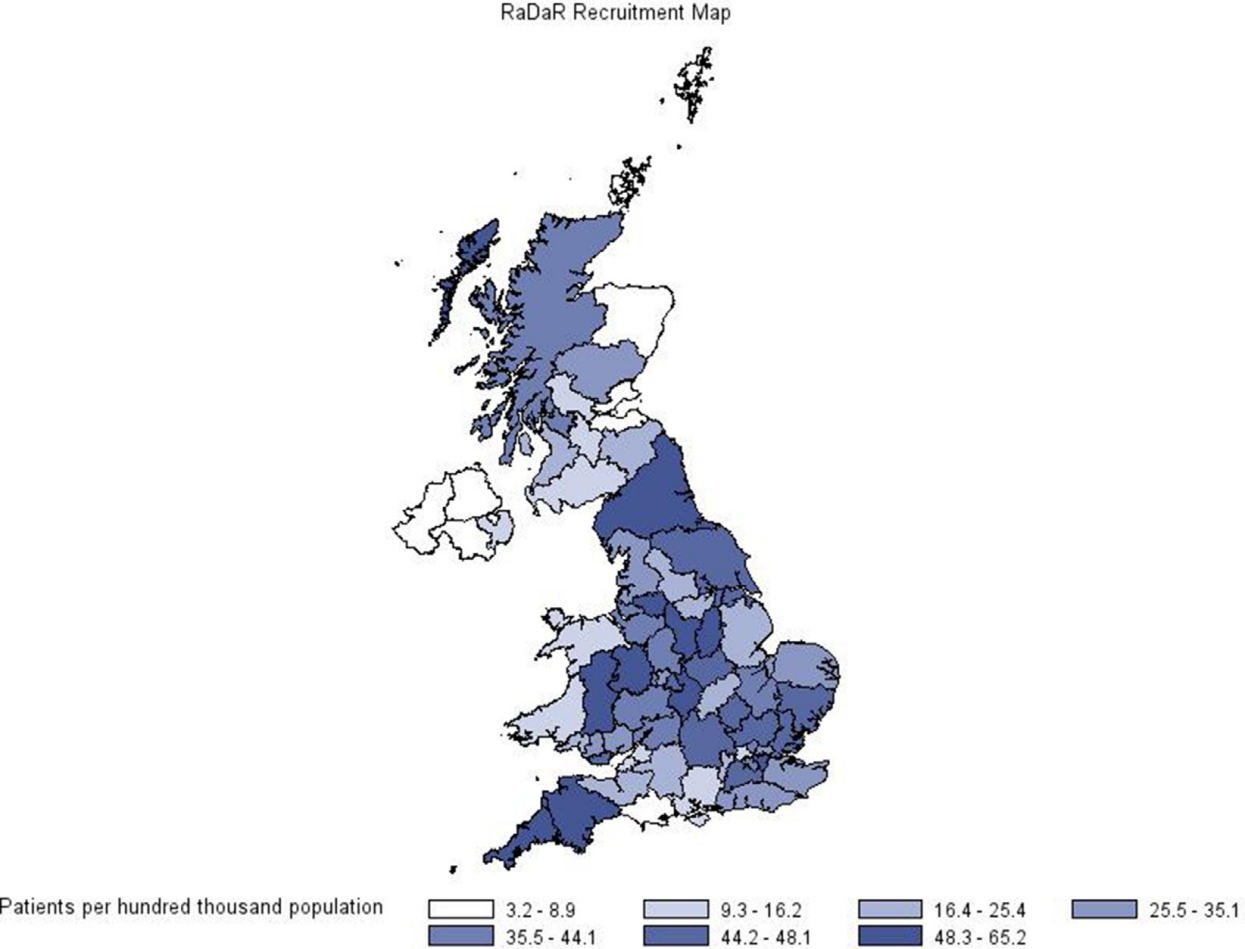
Distribution of recruitment to RaDaR across the United Kingdom. RaDaR, National Registry of Rare Kidney Diseases.

**Figure 4:**
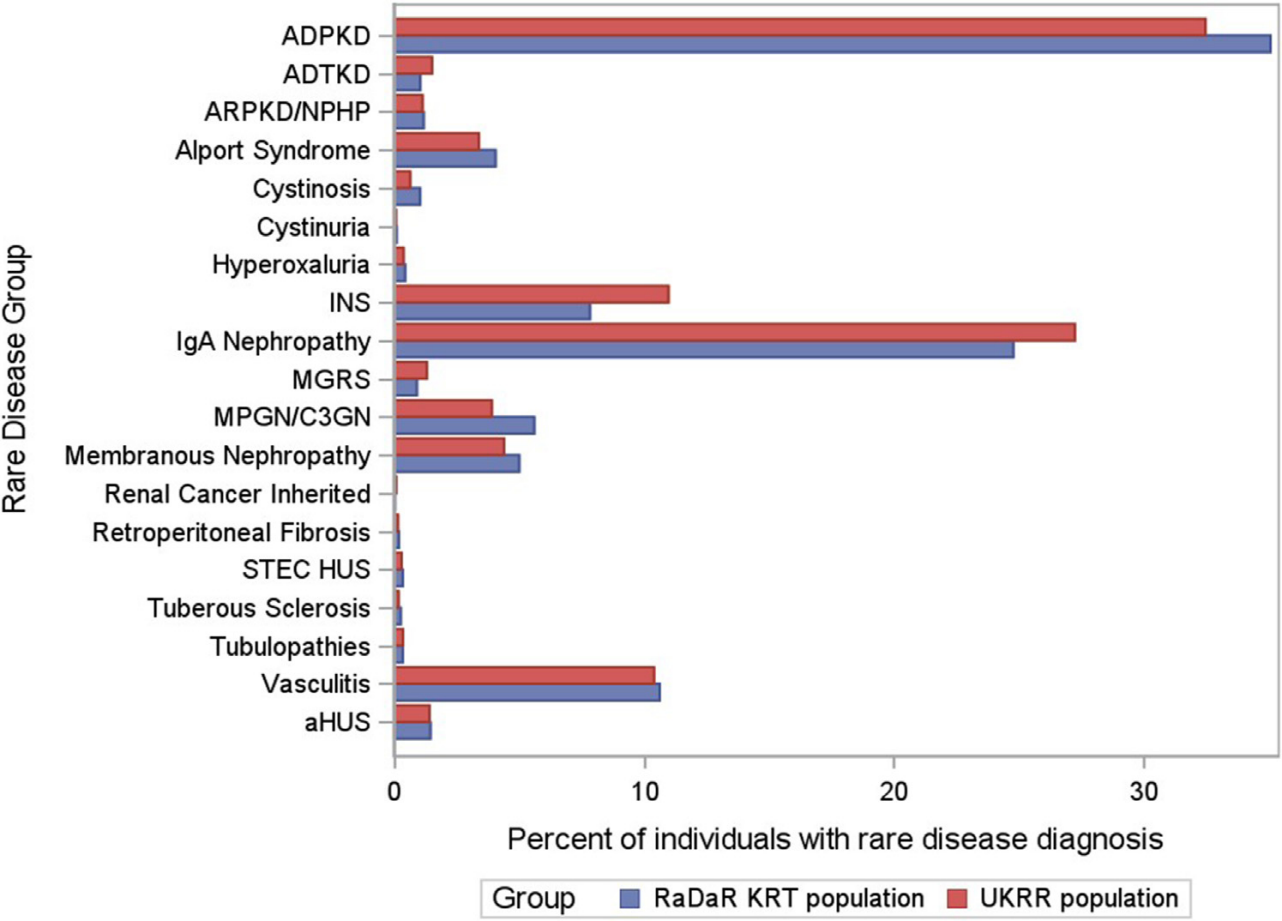
Proportion of rare disease diagnoses for each disorder within UKRR and RaDaR KRT recipients. ADPKD, autosomal dominant polycystic kidney disease; ADTKD, autosomal dominant tubulointerstitial kidney disease; aHUS, atypical hemolytic uremic syndrome; ANCA, antineutrophil cytoplasmic antibody; ARPKD/NPHP, autosomal recessive polycystic kidney disease and nephronophthisis; C3GN, C3 glomerulopathy; FSGS, focal segmental glomerulosclerosis; GBM, glomerular basement membrane; INS, idiopathic nephrotic syndrome; IQR, interquartile range; KRT, kidney replacement therapy; MCD, minimal change disease; MGRS, monoclonal gammopathy of renal significance; MPGN, membranoproliferative glomerulonephritis; NR, not reported; RaDaR, National Registry of Rare Kidney Diseases; SRNS, steroid resistant nephrotic syndrome; SSNS, steroid sensitive nephrotic syndrome; STEC HUS, Shiga toxin/verotoxin-producing Escherichia coli-associated hemolytic uremic syndrome; TBMN, thin basement membrane nephropathy; UKRR, UK Renal Registry.

Geographic distribution of recruitment to RaDaR across the UK is shown in Figure 3. Comparison of RaDaR with UKRR rare disease KRT populations (Figure 4 and Supplementary Table S13) demonstrated similar distributions in both populations, and statistical testing did not show significant evidence of differences (Cramer’s V = 0.07).

Overall, patients recruited to RaDaR with KF were less likely to be Asian (7% vs. 9%, *P* < 0.0001) compared with unrecruited patients in the UKRR dataset (Table 4). However, there was no significant difference between the ethnic distribution of recruited versus unrecruited patients in the following RDGs: autosomal recessive polycystic kidney disease and nephronophthisis (Chi^2^ *P* = 0.41), Alport syndrome (*P* = 0.76), atypical hemolytic uremic syndrome (*P* = 0.55), cystinosis (*P* = 0.71), primary hyperoxaluria (*P* = 0.49), membranoproliferative glomerulonephritis and C3 glomerulopathy (*P* = 0.15), *Shiga* toxin/verotoxin-producing Escherichia coli-associated hemolytic uremic syndrome (*P* = 0.62), and membranous nephropathy (*P* = 0.44). UKRR patients in the least deprived quintile were more likely to be recruited to RaDaR than those in the most deprived quintile (21% vs. 17%, *P* < 0.0001).

**Table 4:** Ethnicity and socioeconomic status comparisons between RaDaR recruited versus unrecruited patients with rare kidney diagnoses at 2 UK renal units and between recruited patients with kidney failure and UKRR patients with rare kidney diseases

Ethnicity								Socioeconomic status (Index of Multiple Deprivation Quintiles)							
Unit 1															
	All eligible patients		Recruited to RaDaR		Not recruited to RaDaR		*P*-value		All eligible patients		Recruited to RaDaR		Not recruited to RaDaR		*P*-value
	*n* (%)		*n* (%)		*n* (%)				*n* (%)		*n* (%)		*n* (%)		
White	2234	(58)	473	(67)	1761	(57)	*P* < 0.0001	1- most deprived	719	(16)	158	(12)	561	(18)	*P* < 0.0001
Mixed	114	(3)	19	(3)	95	(3)		2	1,167	(26)	312	(24)	855	(27)	
Asian	625	(16)	122	(17)	503	(16)		3	1,044	(23)	302	(23)	742	(24)	
Black	483	(13)	56	(8)	427	(14)		4	845	(19)	282	(22)	563	(18)	
Other	366	(10)	41	(6)	325	(10)		5- least deprived	675	(15)	255	(19)	420	(13)	
Total	3822	(100)	711	(100)	3111	(100)		Total	4450	(100)	1309	(100)	3141	(100)	

Unit 2															
	All eligible patients		Recruited to RaDaR		Not recruited to RaDaR		*P*-value		All eligible patients		Recruited to RaDaR		Not recruited to RaDaR		*P*-value
	*n* (%)		*n* (%)		*n* (%)						*n* (%)		*n* (%)		
White	774	(63)	199	(62)	575	(64)	*P* = 0.23	1- most deprived	292	(18)	92	(19)	200	(18)	*P* = 0.67
Mixed	9	(1)	5	(2)	4	(0)		2	442	(28)	138	(28)	304	(28)	
Asian	138	(11)	41	(13)	97	(11)		3	366	(23)	122	(25)	244	(22)	
Black	260	(21)	64	(20)	196	(22)		4	268	(17)	82	(17)	186	(17)	
Other	47	(4)	14	(4)	33	(4)		5- least deprived	223	(14)	61	(12)	162	(15)	
Total	1228	(100)	323	(100)	905	(100)		Total	1591	(100)	495	(100)	1096	(100)	

UK Renal Registry comparison															
	All eligible patients		Recruited to RaDaR		Not recruited to RaDaR		*P*-value		All eligible patients		Recruited to RaDaR		Not recruited to RaDaR		*P*-value
	*n* (%)		*n* (%)		*n* (%)				*n* (%)		*n* (%)		*n* (%)		
White	21986	(85)	5655	(87)	16331	(86)	*P* < 0.0001	1- most deprived	5507	(22)	1152	(18)	4355	(23)	*P* < 0.0001
Mixed	244	(1)	73	(1)	171	(1)		2	5519	(22)	1260	(20)	4259	(23)	
Asian	2235	(9)	468	(7)	1767	(9)		3	5462	(22)	1357	(21)	4105	(22)	
Black	1080	(4)	250	(4)	830	(4)		4	4153	(16)	1355	(21)	2798	(15)	
Other	338	(1)	74	(1)	264	(1)		5- least deprived	4589	(18)	1339	(21)	3250	(17)	
Total	25883	(100)	6520	(100)	19363	(100)		Total	25230	(100)	6463	(100)	18767	(100)	

UKRR, UK Renal Registry; RaDaR, National Registry of Rare Kidney Diseases.

Results comparing recruitment to RaDaR at 2 large renal centers were conflicting; comparing recruited and nonrecruited but eligible patients at each center, and excluding patients with ethnicity not recorded, there were no differences in ethnicity observed in 1 center (recruited vs. nonrecruited; White, 62% vs. 64%; Mixed, 2% vs. 0%; Asian, 13% vs. 11%; Black, 20% vs. 22%; Other ethnicity, 4% vs. 4%; *P* < 0.23), evidence of overrecruitment of White (67% vs. 57%, *P* < 0.0001), and underrecruitment of Black patients (8% vs. 14%, *P* < 0.0001) in the other center. Similarly, there was no evidence of difference in social deprivation in 1 center (recruited vs. non recruited; IMD quintile 1-most deprived: 19% vs. 18%, quintile 2: 28% vs. 28%, quintile 3: 25% vs. 22%, quintile 4: 17% vs. 17%, quintile 5-least deprived: 12% vs. 15%, *P* < 0.67), whereas there was evidence of overrecruitment of patients in the least deprived quintile (19% vs. 13%, *P* < 0.0001) in the other.

Prevalent English patients with a diagnosis of ADPKD were compared with the 2011 English Census, adjusted for age (Supplementary Figure S5). Deviation from the ethnic distribution of the English population varied by age group. More patients aged <40 years were White than the general population (0–17 years: 83% vs. 79%, *P* = 0.006; 18–29 years: 92% vs. 81%, *P* = 0.001; 30–39 years, 92% vs. 81%, *P* < 0.0001). However, patients aged 60 to 69 years were more likely to be Black than the general population (3% vs. 1%, *P* < 0.0001). There was no difference in ethnic distribution for individuals aged >80 years (*P* = 0.39).

## Discussion

We have presented cross-sectional analyses for 1934 (7%) pediatric and 23,946 (93%) adult patients with rare kidney diseases enrolled into RaDaR. To our knowledge, this is the largest epidemiological description of rare kidney diseases worldwide.

RaDaR is not a population-based registry; and therefore, cannot offer precise incidence or prevalence data on individual rare kidney disorders. However, RaDaR patient numbers have allowed us to provide minimum point prevalence UK estimates for 27 RDGs (Table 2), in some cases, for the first time. In addition, patient numbers and demographics may be useful in assessing feasibility of studies or clinical trials in individual rare kidney diseases. Comparison of RaDaR KRT recipients with UK recipients of KRT with a rare disease recorded in UKRR indicated that a similar proportion (approximately 40%) of all patients with eligible rare diseases with KF were enrolled in RaDaR, indicating that in this group, RaDaR allows comparison of prevalence of the relevant diseases without detectable bias according to disease (Figure 4). Similar analyses could not be done in the non-KRT population because there is no comprehensive registry in this group to compare with.

After comparison with multiple data sources, we found that though there was likely to be imperfect representation of all socioeconomic and ethnic groups in RaDaR, these biases do not appear to be of a magnitude likely to distort inferences about epidemiology or natural history of RaDaR diseases. Although patients with KF in RaDaR are more likely to be White than eligible patients in the UKRR (87% vs. 85%, *P*-value < 0.0001), comparison of the English RaDaR ADPKD cohort with the English census and comparison with patients at 2 large renal units found no consistent ethnic recruitment bias to RaDaR. For 7 RDGs (autosomal recessive polycystic kidney disease and nephronophthisis, Alport syndrome, atypical hemolytic uremic syndrome, Shiga toxin/verotoxin-producing Escherichia coli-associated hemolytic uremic syndrome, cystinosis, primary hyperoxaluria, membranoproliferative glomerulonephritis and C3 glomerulopathy, and membranous nephropathy) patients with KF recruited to RaDaR closely matched the ethnic distribution of patients in the UKRR. Patients recruited to RaDaR were more likely to be from the least deprived quintile compared to the UKRR (21% vs. 18%, *P*-value < 0.0001), whereas there was no evidence of overrecruitment of patients in the least deprived quintile within a large renal unit (12% vs. 14%, *P*-value = 0.67). Future work will include investigating these differences to identify potential inequity and to target future recruitment strategies.

Despite evidence that patients of White ethnicities may be overrepresented in the RaDaR KF population, we found patients with cystinosis and primary hyperoxaluria were less likely to be White (48% and 45%, respectively vs. 70%), and more likely to be from Asian backgrounds (16% and 25% respectively vs. 6%) compared to the overall ethnic distribution of RaDaR. These differences were also present when stratified by pediatric and adult RaDaR patients, although pediatric data should be interpreted with caution due to the small patient numbers. This ethnic predisposition has been previously reported in cystinosis, with a high birth frequency rate (1:3600) reported in Pakistani ethnic groups in the West Midlands.^18^ However, to our knowledge it has not previously been reported in primary hyperoxaluria. Previous population analyses have suggested primary hyperoxaluria is 3 times more prevalent among European Americans than African Americans,^19^ and that certain PH1 gene (AGXT) variants have a strong association with people from Spanish or North African backgrounds.^20^ Although a possible mutational hotspot in PH3 gene HOGA1 has been identified in the Chinese population,^21^ none of the patients in the RaDaR primary hyperoxaluria cohort were from a Chinese background. As for any autosomal recessive diseases, the frequency of consanguinity in the community may impact on the incidence of cystinosis and primary hyperoxaluria. Differences in disease frequency in different self-reported ethnic groups suggest that genetic ancestry could influence the likelihood of certain diseases explaining a patient’s symptoms and, whereas the differences described here do not seem large enough to justify targeted population screening, awareness of these differences may help clinicians better investigate patients. It should also be noted that in the absence of genomic data, inferences linking self-reported ethnicity (as ascertained in this study) with genetic ancestry should be made with caution.

Pediatric patients were more likely to be from Asian backgrounds compared to adults. This is likely due to the higher proportion of idiopathic nephrotic syndrome in the pediatric group (44% children vs. 13% adults), conditions that have been reported to affect South Asians up to 5 times more frequently than Europeans.^22^^,^^23^

More pediatric patients were in the lowest IMD quintile compared to adults. In the UK, children are more likely to live in more deprived areas compared to adults, and people of Asian and Black ethnicity are more likely to live in areas with the worst levels of social deprivation than those of White ethnicity.^24^ However, the proportion of English children recruited to RaDaR living in the most deprived IMD quintile exceeded that of children in the general English population. Differences in ethnicity and the proportion of monogenic and nonmonogenic conditions between the pediatric and adult RaDaR populations do not completely explain this disparity; children from White (24.8% vs. 16.2%, *P*-value < 0.001), Asian (53.5% vs. 31.1%, *P*-value < 0.0001), and Other (50.0% vs. 24.7%, *P*-value = 0.017) ethnicities were all more likely to live in more socially deprived areas than adults, as were children diagnosed with both monogenic (30.5% vs. 16.1%, *P*-value < 0.0001) and nonmonogenic (30.2% vs. 18.2%, *P*-value < 0.0001) disorders. Pediatric patients may be more intensively recruited to RaDaR from centers in areas of worst deprivation, either due to clinician interest or a higher population prevalence of certain rare kidney diseases in those areas. However, rare diseases are associated with a high economic burden for patients, especially for families with children,^25^ perhaps explained by the additional caring responsibilities imposed on adults responsible for a child (or children) affected by a rare kidney disease reducing their capacity to earn money (for example, owing to frequent hospital visit attendances for appointments or dialysis). In addition, children from socioeconomically deprived backgrounds experience poorer health outcomes,^26^^,^^27^ and there is evidence of reduced access to preemptive kidney transplantation in UK pediatric kidney patients from more deprived areas.^28^ These findings therefore highlight that children with rare kidney diseases recruited to RaDaR are a potentially highly vulnerable group; further investigation is needed to determine whether they experience different outcomes.

Limitations of this study include the fact that RaDaR is a UK registry and is representative of the mainly White UK population and may not be generalizable to other ethnicities. Survivor bias may have had an impact on the enrolment of individuals with diagnoses made before RaDaR started recruiting patients with that condition. Some RaDaR diagnoses are poorly captured by European Renal Association-European Dialysis and Transplant Association primary renal diagnosis codes, which limited comparison to UKRR data. Entry of rare disease diagnoses into renal information technology systems is user-dependent and may vary between renal units used for comparisons. Although bias could be introduced owing to the variation in recruitment between centers across the UK, and therefore by variation in their catchment population, we sought to minimize effects of this bias by comparing ethnicity and socioeconomic status of each RDG to the overall RaDaR breakdown. Caution must still be exercised where clinicians with particular interest in a certain RDG recruit more intensively into that one RDG compared to others.

In summary, to our knowledge RaDaR is the largest registry of rare kidney diseases worldwide and provides numerous opportunities to advance understanding of rare kidney diseases, including identification of potential participants in clinical trials.

## Appendix

### List of the National Registry of Rare Kidney Diseases (RaDaR) Consortium

Sharirose Abat,^1^ Shazia Adalat,^2^ Joy Agbonmwandolor,^3^ Zubaidah Ahmad,^4^ Abdulfattah Alejmi,^5^ Rashid Almasarwah,^6^ Nicholas Annear,^1^ Ellie Asgari,^4^ Amanda Ayers,^7^ Jyoti Baharani,^8^ Gowrie Balasubramaniam,^9^ Felix Jo-Bamba Kpodo,^10^ Tarun Bansal,^11^ Alison Barratt,^12^ Jonathan Barratt^79^, Megan Bates,^13^ Natalie Bayne,^14^ Janet Bendle,^15^ Sarah Benyon,^16^ Carsten Bergmann,^17,18^ Sunil Bhandari,^19^ Coralie Bingham,^20^ Preetham Boddana,^21^ Sally Bond,^22^ Fiona Braddon,^23^ Kate Bramham,^23^ Angela Branson,^15^ Stephen Brearey,^24^ Vicky Brocklebank,^25^ Sharanjit Budwal,^26^ Conor Byrne,^27^ Hugh Cairns,^28^ Brian Camilleri^29^, Gary Campbell^30^, Alys Capell^31^, Margaret Carmody,^8^ Marion Carson^32^, Tracy Cathcart,^19^ Christine Catley,^9^ Karine Cesar^33^, Melanie Chan,^6^ Houda Chea,^15^ James Chess^34^, Chee Kay Cheung,^26^ Katy-Jane Chick^35^, Nihil Chitalia^36^, Martin Christian^37^, Tina Chrysochou^38,39^, Katherine Clark^40^, Christopher Clayton^41^, Rhian Clissold,^20^ Helen Cockerill^33^, Joshua Coelho^42^, Elizabeth Colby^43^, Viv Colclough^44^, Eileen Conway^45^, H. Terence Cook^46^, Wendy Cook^47^, Theresa Cooper^48^, Richard J Coward^43^, Sarah Crosbie,^22^ Gabor Cserep^49^, Anjali Date^50^, Katherine Davidson^48^, Amanda Davies^51^, Neeraj Dhaun^52^, Ajay Dhaygude^53^, Lynn Diskin,^12^ Abhijit Dixit^41,54^, Eunice Ann Doctolero^35^, Suzannah Dorey^55^, Lewis Downard,^23^ Mark Drayson^56^, Gavin Dreyer,^27^ Tina Dutt^57^, Kufreabasi Etuk,^28^ Dawn Evans^58^, Jenny Finch^29^, Frances Flinter^59^, James Fotheringham^60^, Lucy Francis^82^, Daniel P. Gale^61^, Hugh Gallagher^62^, David Game,^4^ Eva Lozano Garcia^42^, Madita Gavrila,^22^ Susie Gear^63^, Colin Geddes^64^, Mark Gilchrist^65^, Matt Gittus^66^, Paraskevi Goggolidou^67^, Christopher Goldsmith^57^, Patricia Gooden^68^, Andrea Goodlife,^26^ Priyanka Goodwin^53^, Tassos Grammatikopoulos,^28,69^ Barry Gray^70^, Megan Griffith^46^, Steph Gumus,^9^ Sanjana Gupta^71^, Patrick Hamilton^72^, Lorraine Harper^56^, Tess Harris^73^, Louise Haskell^74^, Samantha Hayward^43^, Shivaram Hegde^75^, Bruce Hendry^76^, Sue Hewins^77^, Nicola Hewitson^78^, Kate Hillman,^15^ Mrityunjay Hiremath^57^, Alexandra Howson^79^, Zay Htet,^28^ Sharon Huish,^16^ Richard Hull,^1^ Alister Humphries^68^, David P. J. Hunt^119^, Karl Hunter^80^, Samantha Hunter,^19^ Marilyn Ijeomah-Orji,^6^ Nick Inston^81^, David Jayne^82^, Gbemisola Jenfa^31^, Alison Jenkins^83^, Sally Johnson^118^, Caroline A Jones^84^, Colin Jones^85^, Amanda Jones,^5^ Rachel Jones^82^, Lavanya Kamesh^81^, Durga Kanigicherla^39^, Fiona Karet Frankl^82^, Mahzuz Karim^86^, Amrit Kaur^87^, David Kavanagh,^25^ Kelly Kearley^88^, Larissa Kerecuk,^14^ Arif Khwaja^70^, Garry King,^23^ Grant King^89^, Ewa Kislowska,^4^ Edyta Klata^29^, Maria Kokocinska,^14^ Mark Lambie^90^, Laura Lawless^41^, Thomas Ledson^80^, Rachel Lennon^91^, Adam P Levine^92^, Ling Wai Maggie Lai,^16^ Graham Lipkin^81^, Graham Lovitt^93^, Paul Lyons^94^, Holly Mabillard^95^, Katherine Mackintosh,^7^ Khalid Mahdi^96^, Eamonn Maher^97^, Kevin J. Marchbank,^25^ Patrick B Mark^64^, Sherry Masoud,^23^ Bridgett Masunda,^9^ Zainab Mavani^31^, Jake Mayfair,^4^ Stephen McAdoo,^6^ Joanna Mckinnell^98^, Nabil Melhem,^2^ Simon Meyrick^51^, Shabbir Moochhala^61^, Putnam Morgan^99^, Ann Morgan^100,101^ , Fawad Muhammad,^5^ Shona Murray^30^, Kristina Novobritskaya,^22^ Albert CM Ong^66,70^, Louise Oni^102^, Kate Osmaston,^23^ Neal Padmanabhan^64^, Sharon Parkes,^14^ Jean Patrick,^7^ James Pattison,^4^ Riny Paul,^1^ Rachel Percival^103^, Stephen J. Perkins^104^, Alexandre Persu^105,106^, William G Petchey^107^, Matthew C. Pickering^46^, Jennifer Pinney^81^, David Pitcher,^23^ Lucy Plumb^43^, Zoe Plummer,^23^ Joyce Popoola,^1^ Frank Post,^28^ Albert Power^83^, Guy Pratt^56^, Charles Pusey^46^, Ria Rabara,^22^ May Rabuya,^4^Tina Raju^42^, Chadd Javier^108^, Ian SD Roberts,^22^ Candice Roufosse^109^, Adam Rumjon,^28^ Alan Salama^61^, Moin Saleem^43^, RN Sandford^97^, Kanwaljit S. Sandu^110^, Nadia Sarween^81^, John A. Sayer^95^, Neil Sebire^111,112^, Haresh Selvaskandan,^26^ Sapna Shah,^28^ Asheesh Sharma^57^, Edward J Sharples,^22^ Neil Sheerin,^25^ Harish Shetty^53^, Rukshana Shroff^112^, Roslyn Simms^70^, Manish Sinha,^2^ Smeeta Sinha^113^, Kerry Smith^29^, Lara Smith,^15^ Shalabh Srivastava^114^, Retha Steenkamp,^23^ Ian Stott^115^, Katerina Stroud^97^, Pauline Swift^42^, Justyna Szklarzewicz,^26^ Fred Tam^46^, Kay Tan^116^, Robert Taylor^117^, Marc Tischkowitz^97^, Kay Thomas,^4^ Yincent Tse^118^, Alison Turnbull^85^, A. Neil Turner^119^, Kay Tyerman^55^, Miranda Usher^120^, Gopalakrishnan Venkat-Raman^121^, Alycon Walker^122^, Stephen B. Walsh^61^, Aoife Waters^123^, Angela Watt^68^, Phil Webster,^6^ Ashutosh Wechalekar^124^, Gavin Iain Welsh^43^, Nicol West^125^, David Wheeler^61^, Kate Wiles,^27^ Lisa Willcocks^107^, Angharad Williams^33^, Emma Williams^29^, Karen Williams,^4^ Deborah H Wilson^126^, Patricia D. Wilson^127^, Paul Winyard,^9^ Edwin Wong,^25^ Katie Wong,^23^ Grahame Wood^58^, Emma Woodward,^15^ Len Woodward^128^, Adrian Woolf^129^, and David Wright^71^

^1^St George’s University Hospitals NHS Foundation Trust, UK; ^2^Evelina London Children's Hospital, UK; ^3^David Evans Medical Research Centre, Nottingham University Hospital NHS Trust, UK; ^4^Guy’s and St Thomas NHS foundation Trust, UK; ^5^Ysbyty Gwynedd, Betsi Cadwaladr University Health Board, UK; ^6^Imperial College Healthcare NHS Trust, UK; ^7^James Paget University Hospital NHS Foundation Trust, UK; ^8^Heart of England NHS Foundation Trust, Birmingham, UK; ^9^Mid and South Essex NHS Foundation Trust, UK; ^10^Royal Berkshire NHS Foundation Trust, UK; ^11^Bradford Teaching Hospitals NHS Foundation Trust, UK; ^12^Royal United Hospital Bath NHS Trust, UK; ^13^Freeman Hospital, Newcastle Upon Tyne, UK; ^14^Birmingham Women's and Children's NHS Foundation Trust, UK; ^15^Manchester University NHS Foundation Trust, UK; ^16^Royal Devon University Healthcare NHS Foundation Trust, UK; ^17^Medizinische Genetik Mainz, Mainz, Germany; ^18^Department of Medicine, Faculty of Medicine, Medical Center-University of Freiburg, Freiburg, Germany; ^19^Hull University Teaching Hospitals NHS Trust, UK; ^20^Exeter Kidney Unit, Royal Devon University Healthcare NHS Foundation Trust, UK; ^21^Gloucestershire Hospitals NHS Foundation Trust, UK; ^22^Oxford University Hospitals NHS Foundation Trust, UK; ^23^UK Kidney Association, UK; ^24^Countess of Chester NHS Foundation Trust, UK; ^25^National Renal Complement Therapeutics Centre, Newcastle upon Tyne Hospitals NHS Foundation Trust, Newcastle upon Tyne, UK; ^26^University Hospitals of Leicester NHS Trust, UK; ^27^Barts Health NHS Trust, London, UK; ^28^King's College Hospital NHS Foundation Trust, UK; ^29^East Suffolk and North Essex NHS Foundation Trust, UK; ^30^Ninewells Hospital and Medical School, Dundee, UK; ^31^North West Anglia NHS Foundation Trust, UK; ^32^Northern Health and Social Care Trust and Northern Ireland Clinical Research Network; ^33^West Suffolk NHS Foundation Trust, UK; ^34^Morriston Hospital, Swansea Bay Health Board, UK; ^35^Lister Hospital, East and North Hertfordshire NHS Trust, UK; ^36^Dartford and Gravesham NHS Trust, UK; ^37^Nottingham Children’s Hospital, UK; ^38^Salford Royal Hospital, Northern Care Alliance NHS Foundation Trust, Salford, UK; ^39^University of Manchester, UK; ^40^King’s College London, UK; ^41^Nottingham University Hospitals NHS trust, UK; ^42^Epsom and St Helier University Hospitals NHS Trust, UK; ^43^University of Bristol Medical School, Bristol, UK; ^44^Royal Stoke University Hospital, UK; ^45^Manchester Royal Infirmary, UK; ^46^Centre for Inflammatory Disease, Imperial College London, UK; ^47^Nephrotic Syndrome Trust' (NeST), UK; ^48^North Cumbria Integrated Care NHS Foundation Trust, UK; ^49^Colchester General Hospital, UK; ^50^Tameside and Glossop Integrated Care NHS Foundation Trust, UK; ^51^Wye Valley NHS Trust, UK; ^52^BHF Centre for Cardiovascular Science, The Queen's Medical Research Institute, University of Edinburgh, UK; ^53^Lancashire Teaching Hospital, UK; ^54^School of Medicine, University of Nottingham, UK; ^55^Leeds Teaching Hospitals NHS Trust, UK; ^56^University of Birmingham, UK; ^57^Liverpool University Hospitals Foundation NHS Trust, UK; ^58^Salford Royal NHS Foundation Trust, UK; ^59^Department of Clinical Genetics, Guy’s and St Thomas’ NHS Foundation Trust, UK; ^60^Centre for Health and Related Research, School of Population Health, University of Sheffield, UK; ^61^University College London Department of Renal Medicine, Royal Free Hospital, UK; ^62^SW Thames Renal Unit, Epsom and St Helier University Hospitals NHS Trust, UK; ^63^Alport UK, UK; ^64^Queen Elizabeth University Hospital, Glasgow, UK; ^65^College of Medicine and Health, University of Exeter, UK; ^66^Divison of Population Health, University of Sheffield, UK; ^67^University of Wolverhampton, UK; ^68^Patient Representative, UK; ^69^Institute of Liver Studies, King’s College London, UK; ^70^Sheffield Kidney Institute, Sheffield Teaching Hospitals NHS Foundation Trust, UK; ^71^Royal Free Hospital, UK; ^72^Manchester Institute of Nephrology and Transplantation, Manchester Royal Infirmary, UK; ^73^PKD Charity, UK; ^74^University Hospital Southampton NHS Foundation Trust, UK; ^75^Children's Kidney centre, University Hospital of Wales, UK; ^76^Travere Therapeutics, UK; ^77^University Hospitals Coventry and Warwickshire NHS Trust, UK; ^78^County Durham & Darlington NHS Foundation Trust, UK; ^79^University of Leicester, UK; ^80^Wirral University Teaching Hospital NHS Foundation Trust, UK; ^81^University Hospitals Birmingham NHS Foundation Trust, UK; ^82^Department of Medicine, University of Cambridge, UK; ^83^North Bristol NHS Trust, UK; ^84^Alder Hey Childrens NHS Foundation Trust, UK; ^85^York & Scarborough Teaching Hospitals NHS Foundation Trust, UK; ^86^Norfolk and Norwich University Hospitals NHS Trust, UK; ^87^Royal Manchester Children’s Hospital, Manchester, UK; ^88^PTEN UK and Ireland Patient Group; ^89^HNF1B Support Group, UK; ^90^School of Medicine, Keele University, UK; ^91^Wellcome Centre for Cell-Matrix Research, University of Manchester, UK; ^92^Research Department of Pathology, University College London, UK; ^93^HLRCC Foundation, UK; ^94^Cambridge Institute of Therapeutic Immunology and Infectious Disease, Cambridge, UK; ^95^Newcastle University, UK; ^96^United Lincolnshire Hospitals NHS Trust, UK; ^97^Department of Medical Genetics, University of Cambridge, UK; ^98^University Hospitals of Derby and Burton NHS Foundation Trust, UK; ^99^Retroperitoneal Fibrosis (RF) Group, UK; ^100^National Institute of Health and Care Research Leeds Biomedical Research Centre, Leeds Teaching Hospitals NHS Trust, UK; ^101^School of Medicine, University of Leeds, UK; ^102^University of Liverpool, UK; ^103^Newcastle Upon Tyne Hospitals NHS Foundation Trust, UK; ^104^Research Department of Structural and Molecular Biology, University College London, UK; ^105^Division of Cardiology, Cliniques Universitaires Saint-Luc, Belgium; ^106^Pole of Cardiovascular Research, Institut de Recherche Expérimentale et Clinique, Université Catholique de Louvain, Brussels, Belgium; ^107^Cambridge University Hospitals NHS Foundation Trust, UK; ^108^East and North Hertfordshire NHS Trust, UK; ^109^Department of Immunology and Inflammation, Faculty of Medicine, Imperial College London, UK; ^110^Shrewsbury and Telford Hospital NHS Trust, UK; ^111^National Institute of Health and Care Research Great Ormond Street Hospital Biomedical Research Centre, UK; ^112^UCL Great Ormond Street Institute of Child Health, UK; ^113^Northern Care Alliance NHS Foundation Trust, UK; ^114^South Tyneside and Sunderland NHS Foundation Trust, UK; ^115^Doncaster and Bassetlaw Teaching Hospitals, UK; ^116^New Cross Hospital, Wolverhampton, UK; ^117^Wellcome Centre for Mitochondrial Research, Translational & Clinical Research Institute, Faculty of Medical Sciences, Newcastle University, UK; ^118^Great North Children's Hospital, Newcastle Upon Tyne, UK; ^119^University of Edinburgh, UK; ^120^Calderdale & Huddersfield Foundation Trust, UK; ^121^Royal Surrey County Hospital, Guildford, UK; ^122^South Tees Hospitals NHS Foundation Trust, UK; ^123^University College Cork, Ireland; ^124^National Amyloidosis Centre, University College London, UK; ^125^Great Western Hospital, Swindon, UK; ^126^North Tees and Hartlepool NHS Foundation Trust, UK; ^127^University College London, UK; ^128^aHUS Alliance, UK; and ^129^School of Biological Sciences, University of Manchester, UK

## Disclosure

KTLS, KH, and JY declare employment with Pfizer Inc who part-funded the analysis. ERM declares support for the current manuscript from VHL UK/Ireland and has received fees for consulting for MSD. SM is chair of OxalEurope. MS declares support for the current manuscript from a Medical Research Council UK Precision Medicine program grant - MR/R013942/1 and has received fees for consulting for Travere Therapeutics. RJC declares support for the current manuscript from Kidney Research UK. JAS declares support for the current manuscript from Kidney Research UK, Northern Counties Kidney Research Fund, and the Medical Research Council UK (payments to institution) and is Academic Vice President of the UK Kidney Association (UKKA). FWKT declares support from the National Institute for Health and Care Research Imperial Biomedical Centre. DN is the UKKA Director of Informatics Research. DPG declares support for the current manuscript from St Peter’s Trust for Kidney Bladder and Prostate Research, Pfizer Inc, Medical Research Council, Kidney Research UK, Kidney Care UK, and Polycystic Kidney Disease Charity (payments to institution), chairs the Rare Diseases Committee of the UKKA and has received fees for consulting and presenting from Novartis, Alexion, Calliditas, Sanofi, Britannia, and Travere. All the other authors declared no competing interests.

## Supplementary Material

Supplementary File (PDF) **Supplementary Methods. Supplementary References. Figure S1.** Comparison of ethnicity in each rare disease group. **Figure S2.** Comparison of ethnicity in each rare disease group to total ethnicity breakdown of RaDaR, stratified by current (A) pediatric and (B) adult patients. **Figure S3.** Comparison of IMD quintile in each rare disease group to total IMD quintile distribution of RaDaR. **Figure S4.** Comparison of IMD quintile in each rare disease group to total IMD quintile distribution of RaDaR, stratified by current (A) pediatric and (B) adult patients. **Figure S5.** Ethnicity of English RaDaR patients with ADPKD compared to English census. **Table S1.** Missing data analysis for individuals missing date of diagnosis. **Table S2.** Missing data analysis for individuals missing ethnicity. **Table S3.** Missing data analysis for individuals missing IMD quintile data. **Table S4.** European Renal Association-European Dialysis and Transplant Association codes and search terms for each rare disease group. **Table S5.** Ethnicity and IMD quintile, ∗ stratified by current age. **Table S6.** Ethnic and IMD quintile distribution of children (aged ≤18 years) in England∗ compared to the children in RaDaR. **Table S7.** Monogenic and nonmonogenic disorders, stratified by ethnicity. **Table S8.** Monogenic and nonmonogenic disorders, stratified by age category∗ and ethnicity. **Table S9.** Monogenic and nonmonogenic disorders, stratified by ethnicity and IMD quintile. **Table S10.** Socioeconomic status (IMD quintile) of RaDaR participants, stratified by mode of inheritance and age category. ∗ **Table S11.** IMD quintile, stratified by current age and ethnicity. **Table S12.** Socioeconomic status (IMD quintile), stratified by monogenic and nonmonogenic disorders and age category. ∗ **Table S13.** Number and percentage of rare disease diagnoses for each disorder within UKRR and RaDaR KRT recipients. **Table S14.** Recruiting centers. **Table S15.** RaDaR eligibility criteria. **STROBE checklist for reporting observational studies.**
