# Supplementary material for: Description and Cross-Sectional Analyses of 25,880 Adults and Children in the UK National Registry of Rare Kidney Diseases Cohort
Source: Kidney Int Rep. 2024 May 9;9(7):2067–83. doi: 10.1016/j.ekir.2024.04.062 (PMC11284373; doi:10.1016/j.ekir.2024.04.062)
Supplement: Supplementary File (PDF) — Supplementary Methods. Supplementary References. Figure S1. Comparison of ethnicity in each rare disease group. Figure S2. Comparison of ethnicity in each rare disease group to total ethnicity breakdown of RaDaR, stratified by current (A) pediatric and (B) adult patients. Figure S3. Comparison of IMD quintile in each rare disease group to total IMD quintile distribution of RaDaR. Figure S4. Comparison of IMD quintile in each rare disease group to total IMD quintile distribution of RaDaR, stratified by current (A) pediatric and (B) adult patients. Figure S5. Ethnicity of English RaDaR patients with ADPKD compared to English census. Table S1. Missing data analysis for individuals missing date of diagnosis. Table S2. Missing data analysis for individuals missing ethnicity. Table S3. Missing data analysis for individuals missing IMD quintile data. Table S4. European Renal Association-European Dialysis and Transplant Association codes and search terms for each rare disease group. Table S5. Ethnicity and IMD quintile, ∗ stratified by current age. Table S6. Ethnic and IMD quintile distribution of children (aged ≤18 years) in England∗ compared to the children in RaDaR. Table S7. Monogenic and nonmonogenic disorders, stratified by ethnicity. Table S8. Monogenic and nonmonogenic disorders, stratified by age category∗ and ethnicity. Table S9. Monogenic and nonmonogenic disorders, stratified by ethnicity and IMD quintile. Table S10. Socioeconomic status (IMD quintile) of RaDaR participants, stratified by mode of inheritance and age category. ∗ Table S11. IMD quintile, stratified by current age and ethnicity. Table S12. Socioeconomic status (IMD quintile), stratified by monogenic and nonmonogenic disorders and age category. ∗ Table S13. Number and percentage of rare disease diagnoses for each disorder within UKRR and RaDaR KRT recipients. Table S14. Recruiting centers. Table S15. RaDaR eligibility criteria. STROBE checklist for reporting observational studies. [file mmc1.pdf]

Description and cross-sectional analyses of 25,880 adults and children in the UK National Registry of Rare Kidney Diseases cohort

Supplementary Material

Table of Contents

Supplementary Methods....................2

Supplementary References....................2

Supplementary Table 1: Missing data analysis for individuals missing date of diagnosis....................3

Supplementary Table 2: Missing data analysis for individuals missing ethnicity....................4

Supplementary Table 3: Missing data analysis for individuals missing IMD quintile data....................4

Supplementary Table 4: ERA-EDTA codes and search terms for each Rare Disease Group....................5

Supplementary Table 5: Ethnicity and IMD Quintile*, stratified by current age....................10

Supplementary Table 6: Ethnic and IMD Quintile distribution of children (aged ≤18 years) in England* compared to the children in RaDaR....................10

Supplementary Table 7: Monogenic and Non monogenic disorders, stratified by ethnicity....................11

Supplementary Table 8: Monogenic and Non monogenic disorders, stratified by age category* and ethnicity....................11

Supplementary Table 9: Monogenic and Non monogenic disorders, stratified by Ethnicity and IMD Quintile....................11

Supplementary Table 10: Socioeconomic status (IMD Quintile) of RaDaR participants, stratified by mode of inheritance and age category*....................12

Supplementary Table 11: IMD Quintile, stratified by current age and Ethnicity....................13

Supplementary Table 12: Socioeconomic status (IMD Quintile), stratified by monogenic and non monogenic disorders and age category*....................13

Supplementary Table 13: Number and percentage of rare disease diagnoses for each disorder within UKRR and RaDaR KRT recipients....................14

Supplementary Figure 1: Comparison of ethnicity in each Rare Disease Group....................15

Supplementary Figure 2: Comparison of ethnicity in each Rare Disease Group to total ethnicity breakdown of RaDaR, stratified by current a) paediatric b) adult patients....................16

Supplementary Figure 3: Comparison of IMD Quintile in each Rare Disease Group to total IMD Quintile distribution of RaDaR....................17

Supplementary Figure 4: Comparison of IMD Quintile in each Rare Disease Group to total IMD Quintile distribution of RaDaR, stratified by current a) paediatric b) adult patients....................18

Supplementary Figure 5: Ethnicity of English RaDaR patients with ADPKD compared to English census....................19

Supplementary Table 14: Recruiting centres....................20

Supplementary Table 15: RaDaR Eligibility Criteria....................21

STROBE checklist for reporting observational studies..**45**

Supplementary Methods

To assess whether there has been ethnic or socioeconomic status (SES) recruitment bias to RaDaR, three methods were used. Firstly, ethnicity and SES of all prevalent RaDaR patients who had reached kidney failure were compared to patients with a rare kidney diagnosis in the UKRR. All UKRR patients from renal units participating in RaDaR, alive after their renal unit began recruiting to RaDaR, and not already recruited to RaDaR were then identified using the RaDaR list of PRD codes. To ensure an accurate comparison, only RDGs where RaDaR diagnoses were well captured by the UKRR, and where there was unlikely to be a difference between the diagnosis given at start of RRT and a patient's RaDaR diagnosis were included. These RDGs were determined by ascertaining 1) RDGs where >60% of recruited patients with ESKD were identified using the overall RaDaR PRD code list in the UKRR dataset 2) RDGs where <30% of patients had a diagnosis date after commencement of RRT.

Secondly, patients recruited to RaDaR from two large UK renal centres were compared with all unrecruited patients with a RaDaR eligible diagnosis at those centres, alive after those centres began participating in RaDaR (personal communication with KW). Eligible patients were identified from renal IT systems using PRD codes or diagnosis keywords (Supplementary Table 4).

Lastly, the age-stratified ethnicity distribution of England according to the 2011 UK census was compared to the ethnicity of prevalent English RaDaR patients with ADPKD. As a hereditary condition driven by recent or de-novo mutations, ADPKD is likely to affect ethnic groups equally[S1,S2]; ethnic distribution is therefore expected to reflect that reported by the UK census. Persistent deviation from the ethnic distribution reported by the census in this RDG might therefore suggest bias in recruitment to RaDaR.

Supplementary References

S1 Freedman BI, Soucie JM, Chapman A, Krisher J, McClellan WM. Racial variation in autosomal dominant polycystic kidney disease. *American Journal of Kidney Diseases* 2000; **35**: 35–9.

S2 McGill RL, Saunders MR, Hayward AL, Chapman AB. Health Disparities in Autosomal Dominant Polycystic Kidney Disease (ADPKD) in the United States. *Clinical Journal of the American Society of Nephrology* 2022; **17**: 976–85.

Supplementary Table 1: Missing data analysis for individuals missing date of diagnosis

	Not missing n	(%)	Missing n	(%)	P-value*
**Cohort**					
ADPKD	5008	(24.0)	2104	(40.9)	<0.0001
ADTKD	155	(0.7)	36	(0.7)	
X-linked AS- Female	241	(1.2)	55	(1.1)	
X-linked AS- Male	306	(1.5)	79	(1.5)	
TBMN	154	(0.7)	8	(0.2)	
ARPKD/NPHP	167	(0.8)	48	(0.9)	
Cystinosis	129	(0.6)	15	(0.3)	
Cystinuria	409	(2.0)	51	(1.0)	
Hyperoxaluria	96	(0.5)	19	(0.4)	
HNF1b Mutations	68	(0.3)	17	(0.3)	
Renal Cancer Inherited	112	(0.5)	1	(0.0)	
Tubulopathies	305	(1.5)	102	(2.0)	
Tuberous Sclerosis Complex	201	(1.0)	41	(0.8)	
aHUS	221	(1.1)	55	(1.1)	
SSNS/MCD	1621	(7.8)	31	(0.6)	
SRNS/FSGS	1370	(6.6)	40	(0.8)	
INS- unspecified	369	(1.8)	486	(9.4)	
IgA nephropathy	3398	(16.3)	398	(7.7)	
Membranous Nephropathy	1670	(8.0)	385	(7.5)	
MGRS	122	(0.6)	22	(0.4)	
MPGN/C3GN	739	(3.5)	193	(3.7)	
Pregnancy	467	(2.2)	215	(4.2)	
Retroperitoneal Fibrosis	87	(0.4)	24	(0.5)	
STEC HUS	151	(0.7)	16	(0.3)	
ANCA associated vasculitis	1920	(9.2)	4	(0.1)	
Anti-GBM disease	116	(0.6)	0	(0.0)	
Other Vasculitides	1252	(6.0)	705	(13.7)	
**Ethnicity**					
White	14311	(68.9)	3977	(77.8)	<0.0001
Mixed	166	(0.8)	32	(0.6)	
Asian	1491	(7.2)	235	(4.6)	
Black	505	(2.4)	113	(2.2)	
Other	218	(1.0)	42	(0.8)	
Refused/not stated	714	(3.4)	255	(5.0)	
Missing	3366	(16.2)	455	(8.9)	
**IMD Quintile (Socioeconomic Status)****					
1- Most deprived	3886	(18.8)	820	(16.2)	<0.0001
2	3959	(19.2)	934	(18.5)	
3	4208	(20.4)	1017	(20.1)	
4	4312	(20.9)	1111	(22.0)	
5- Least Deprived	4289	(20.8)	1170	(23.2)	

*Chi^2 test **Patients without postcode, or from Channel Islands or Isle of Man excluded

Supplementary Table 2: Missing data analysis for individuals missing ethnicity

	Not missing		Missing		P-value*
	**n**	**(%)**	**n**	**(%)**	
**IMD Quintile (Socioeconomic Status)**					
1- Most deprived	4081	(18.6)	625	(18.6)	0.001
2	4102	(18.7)	791	(18.7)	
3	4439	(20.3)	786	(20.3)	
4	4608	(21.0)	815	(21.0)	
5- Least Deprived	4690	(21.4)	769	(21.4)	
**Age category**					
Paediatric	1517	(6.9)	417	(10.9)	<0.0001
Adults	20542	(93.1)	3404	(89.1)	
**Country****					
England	20057	(91.5)	3498	(92.4)	<0.0001
Northern Ireland	103	(0.5)	43	(1.1)	
Scotland	976	(4.5)	131	(3.5)	
Wales	784	(3.6)	114	(3.0)	

Supplementary Table 3: Missing data analysis for individuals missing IMD quintile data

Not missing		Missing	
n	(%)	n	(%)
25870	(99.9)	10	(0.1)

Supplementary Table 4: ERA-EDTA codes and search terms for each Rare Disease Group

Cohort	ERA-EDTA code	ERA-EDTA code description	Keywords
**ADPKD**	2739	Autosomal dominant (AD) polycystic kidney disease type II	Autosomal dominant polycystic kidney disease, ADPKD, polycystic kidney disease
	2725	Autosomal dominant (AD) polycystic kidney disease type I	
	2718	Autosomal dominant (AD) polycystic kidney disease	
**ADTKD**	2827	Uromodulin-associated nephropathy (familial juvenile hyperuricaemic nephropathy)	Hyperuricaemic Nephropathy, Primary Hyperuricaemic nephropathy, Familial Hyperuricaemic nephropathy, Medullary cystic kidney disease, Autosomal Dominant Tubulointerstitial Kidney Disease, ADTKD, Familial juvenile hyperuricaemic nephropathy, Familial gouty nephropathy, Familial urate nephropathy, Familial interstitial nephropathy, Uromodulin associated nephropathy, UMOD, Medullary cystic kidney disease type I, Medullary cystic kidney disease type II
	2804	Medullary cystic kidney disease type I	
	2815	Medullary cystic kidney disease type II	
	1907	Familial interstitial nephropathy - no histology	
	1911	Familial interstitial nephropathy - histologically proven	
**ARPKD/NPHP**	2891	Nephronophthisis - type 6	Nephronophthisis, NPHP, Autosomal Recessive Polycystic Kidney Disease, ARPKD, Caroli Syndrome with kidney malformation, Caroli Syndrome with cyst, Congenital Hepatic Fibrosis, Nephronophthisis type 1, Nephronophthisis type 2, Nephronophthisis type 3, Nephronophthisis type 4, Nephronophthisis type 5, Nephronophthisis type 6, Senior-Loken, senior–løken, Senior-Loken syndrome, senior–løken syndrome, Joubert, Joubert syndrome, Meckel-Gruber, Meckel-Gruber syndrome, Cogan, Cogan syndrome, Sensenbrenner, Sensenbrenner syndrome, ATD, Asphyxiating thoracic dystrophy, Asphyxiating thoracic dystrophy of the newborn, Chondroectodermal dysplasia-like syndrome, Infantile thoracic dystrophy, JATD, Jeune asphyxiating thoracic dystrophy, Jeune's syndrome, Thoracic pelvic phalangeal dystrophy, Alström, Alström–Hallgren, Alström syndrome, Alström–Hallgren syndrome, Boichis, Senior-Boichis, Boichis syndrome, Senior-Boichis syndrome, Arima, Arima syndrome, Mainzer-Saldino, Mainzer-Saldino syndrome, Bardet-Biedl, Bardet-Biedl syndrome, BBS, Chondroectodermal dysplasia, Ellis Van Creveld syndrome, Mesodermic dysplasia, Mesoectodermal dysplasia
	2741	Autosomal recessive (AR) polycystic kidney disease	
	2836	Nephronophthisis	
	2858	Nephronophthisis - type 2 (infantile type)	
	2843	Nephronophthisis - type 1 (juvenile type)	
	2870	Nephronophthisis - type 4 (juvenile type)	
	2862	Nephronophthisis - type 3 (adolescent type)	
	2889	Nephronophthisis - type 5	
**Alport Syndrome**	2756	Alport syndrome - no histology	Alport syndrome, Alport's syndrome, Alport carrier, Alport, Alport's, Thin basement membrane nephropathy, TBMN, Autosomal Alport syndrome, Autosomal Alport's syndrome, X linked Alport syndrome, X linked Alport's syndrome, X-linked Alport syndrome, X-linked Alport's syndrome
	2787	Thin basement membrane disease	
	2760	Alport syndrome - histologically proven	
**Atypical HUS**	2668	Familial haemolytic uraemic syndrome (HUS)	Atypical haemolytic uraemic syndrome, atypical HUS, aHUS, diarrhoea negative HUS, Congenital HUS, Familial HUS, thrombotic microangiopathy, TMA
	2652	Congenital haemolytic uraemic syndrome (HUS)	
	2623	Atypical haemolytic uraemic syndrome (HUS) - diarrhoea negative	
**Cystinosis**	2964	Cystinosis	Cystinosis
**Cystinuria**	2955	Cystinuria	Cystinuria

**Hyperoxaluria**	3207	Primary hyperoxaluria type I	Hyperoxaluria, Primary hyperoxaluria, Oxalosis, Primary Hyperoxaluria Type 1, Primary Hyperoxaluria Type 2, Primary Hyperoxaluria Type 3
	3731	Primary hyperoxaluria type III	
	3211	Primary hyperoxaluria type II	
	3194	Primary hyperoxaluria	
	3194	Primary hyperoxaluria	
**HNF1b mutations**	1656	Glomerulocystic Disease	HNF1b, Hepatocyte nuclear factor-1B, Hepatocyte nuclear factor 1B mutation, Hepatocyte nuclear factor 1B, Renal cysts and diabetes, RCAD, Inherited genetic diabetes type 2, MODY 5
	1639	Multicystic Dysplastic Kidneys	
	3627	Renal Cysts & Diabetes Syndrome	
	3139	Diabetes - Type II MODY - Inherited/Genetic	
**Idiopathic Nephrotic Syndrome**	1100	Minimal change nephropathy - histologically proven	Primary focal segmental glomerulosclerosis, Primary FSGS, Nephrotic syndrome, NS, INS, Congenital nephrotic syndrome, Congenital NS, Steroid resistant nephrotic syndrome, SRNS, Steroid sensitive nephrotic syndrome, SSRS, Nail Patella Syndrome, Nail-patella syndrome, Denys Drash Syndrome, Denys-Drash Syndrome, Minimal change disease, MCD
	1349	Mesangial proliferative glomerulonephritis	
	3604	Nephrotic syndrome of childhood - steroid resistant - no histology	
	1090	Minimal change nephropathy - no histology	
	3604	Nephrotic syndrome of childhood - steroid resistant - no histology	
	1090	Minimal change nephropathy - no histology	
	1280	Familial focal segmental glomerulosclerosis (FSGS) - autosomal recessive - histologically proven	
	3615	Nephrotic syndrome of childhood - no trial of steroids - no histology	
	1074	Denys-Drash syndrome	
	1003	Adult nephrotic syndrome - no histology	
	1100	Minimal change nephropathy - histologically proven	
	1279	Familial focal segmental glomerulosclerosis (FSGS) - autosomal recessive - no histology	
	1090	Minimal change nephropathy - no histology	
	1090	Minimal change nephropathy - no histology	
	1100	Minimal change nephropathy - histologically proven	
	1019	Nephrotic syndrome of childhood - steroid sensitive - no histology	
	1100	Minimal change nephropathy - histologically proven	
	1042	Congenital nephrotic syndrome (CNS) - Finnish type - histologically proven	
	1298	Familial focal segmental glomerulosclerosis (FSGS) - autosomal dominant - no histology	
	1035	Congenital nephrotic syndrome (CNS) - Finnish type - no histology	
	1088	Congenital nephrotic syndrome (CNS) - congenital infection	
	1057	Congenital nephrotic syndrome (CNS) - diffuse mesangial sclerosis	
	1026	Congenital nephrotic syndrome (CNS) - no histology	
	1308	Familial focal segmental glomerulosclerosis (FSGS) - autosomal dominant - histologically proven	

	1026	Congenital nephrotic syndrome (CNS) - no histology	
	1061	Congenital nephrotic syndrome (CNS) - focal segmental glomerulosclerosis (FSGS)	
	3253	Nail-patella syndrome	
	1267	Primary focal segmental glomerulosclerosis (FSGS)	
**IgA Nephropathy**	1128	IgA nephropathy - histologically proven	IgA nephropathy, IgA, IgAN
	1159	IgA nephropathy secondary to liver cirrhosis - no histology	
	1144	Familial IgA nephropathy - histologically proven	
	1137	Familial IgA nephropathy - no histology	
	1163	IgA nephropathy secondary to liver cirrhosis - histologically proven	
**Inherited Renal Cancer Syndromes**	3282	Von Hippel-Lindau disease	Von Hippel Lindau disease, VHL, PTEN hamartoma tumour syndrome, Cowden syndrome, Birt Hogg Dube syndrome, Birt-Hogg-Dube Syndrome, BHD, Hereditary leiomyomatosis and renal cell cancer syndrome, HLRCC, Succinate dehydrogenase-related tumour predisposition syndrome, BAP1-related tumour predisposition syndrome, Hereditary Type 1 papillary renal cell carcinoma syndrome
**MGRS**	2584	Myeloma cast nephropathy - histologically proven	AH amyloidosis, AHL amyloidosis, C3 glomerulonephritis with monoclonal gammopathy, C3G with monoclonal gammopathy, C3GN with monoclonal gammopathy, Crystalglobulinaemia, Crystal-storing histiocytosis, Fibrillary Glomerulonephritis, Fibrillary GN, Immunotactoid Glomerulonephritis with Organised Microtubular Monoclonal Immunoglobulin Deposits, Immunotactoid GN with Organised Microtubular Monoclonal Immunoglobulin Deposits, GOMMID, Intracapillary monoclonal IgM without cryoglobulin, Intraglomerular lymphoma, Intraglomerular leukaemia, Intracapillary lymphoma, Intracapillary leukaemia, Light chain cast nephropathy, Light chain proximal tubulopathy, crystalline light chain proximal tubulopathy, non crystalline light chain proximal tubulopathy, Monoclonal Immunoglobulin Deposition Disease, MIDD, Light Chain Deposition Disease, LCDD, Heavy Chair Deposition Disease, HCDD, Light and Heavy Chain Deposition Disease, LHCDD, Proliferative glomerulonephritis with monoclonal immunoglobulin deposits, PGNMID, Thrombotic Microangiopathy with monoclonal gammopathy, Type 1 cryoglobulinaemic Glomerulonephritis, Type I cryoglobulinaemic GN, Unclassified MGRS, MGRS
	2606	Immunotactoid / fibrillary nephropathy	
	2597	Light chain deposition disease	
	2521	AL amyloid secondary to plasma cell dyscrasia	
**MPGN/C3GN**	1233	Mesangiocapillary glomerulonephritis type 2 (dense deposit disease)	Membranoproliferative glomerulonephritis, MPGN, Mesangiocapillary glomerulonephritis, MCGN, Dense Deposit Disease, DDD, C3 Glomerulonephritis, C3 Glomerulopathy, C3GN, C3G, Membranoproliferative glomerulonephritis Type I, MPGN Type I, MPGN I, Membranoproliferative glomerulonephritis Type II, MPGN Type II, MPGN II
	1222	Mesangiocapillary glomerulonephritis type 1	
	1246	Mesangiocapillary glomerulonephritis type 3	
	1233	Mesangiocapillary glomerulonephritis type 2 (dense deposit disease)	
**Membranous Nephropathy**	1214	Membranous nephropathy - infection associated	Membranous nephropathy, Membranous
	1205	Membranous nephropathy - drug induced	
	1185	Membranous nephropathy - idiopathic	
	1192	Membranous nephropathy - malignancy associated	
**Pregnancy**	NA	NA	Pregnancy
	3689	Retroperitoneal fibrosis secondary to drugs	

**Retroperitoneal Fibrosis**	3670	Retroperitoneal fibrosis secondary to peri-aortitis	Retroperitoneal fibrosis, RPF, IgG4-related Vasculitis, IgG4 related vasculitis, IgG4-related disease, IgG4RD, Periaortitis, Aortitis
	1813	Idiopathic retroperitoneal fibrosis	
**STEC HUS**	2610	Haemolytic uraemic syndrome (HUS) - diarrhoea associated	STEC HUS, STEC-HUS, Shiga toxin associated HUS, Shiga toxin associated Haemolytic Uraemic Syndrome, Shiga toxin veryocytotoxin associated HUS, Shiga toxin veryocytotoxin associated Haemolytic uraemic syndrome
**Tuberous Sclerosis Complex**	3276	Tuberous sclerosis	Tuberous sclerosis, TS, Tuberous Sclerosis Complex, TSC
**Tubulopathy**	3187	Familial hypomagnesaemia	Tubulopathy, Dominant hypophosphatemia with nephrolithiasis or osteoporosis, Drug induced Fanconi syndrome, Drug induced hypomagnesemia, Drug induced Nephrogenic Diabetes Insipidus, EAST syndrome, EAST, Epilepsy Ataxia Sensorineural deafness Tubulopathy syndrome, Epilepsy Ataxia Sensorineural deafness Tubulopathy, Familial Hypomagnesaemia with hypercalciuria and nephrocalcinosis, CLDN16/19, Familial primary hypomagnesemia with hypocalcuria, FXYD2, Familial primary hypomagnesemia with normocalcuria, EGF, Familial renal glucosuria, SLC5A2, Fanconi syndrome, Fanconi Renotubular syndrome 1, FRTS1, Fanconi Renotubular syndrome 2, FRTS2, Fanconi Renotubular syndrome 3, FRTS3, Generalized pseudohypoaldosteronism type 1, Heavy metal induced Fanconi syndrome, Heavy metal Fanconi syndrome, Hereditary renal hypouricemia, Hereditary hypophosphatemic rickets with hypercalciuria, Isolated autosomal dominant hypomagnesemia, Glaudemans type, Glaudemans, Glaudeman's, Liddle syndrome, Liddle, Liddle's, Nephrogenic diabetes insipidus, Nephrogenic DI, Nephrogenic syndrome of inappropriate antidiuresis, Nephrogenic SIADH, Oncogenic osteomalacia, Osteopetrosis with renal tubular acidosis, Osteopetrosis with RTA, Primary hypomagnesemia with secondary hypocalcemia, Pseudohypoaldosteronism type 2A, Pseudohypoaldosteronism type 2B, Pseudohypoaldosteronism type 2C, Pseudohypoaldosteronism type 2D, Pseudohypoaldosteronism type 2E, Renal pseudohypoaldosteronism type 1, Autoimmune distal renal tubular acidosis, Autoimmune distal RTA, Autosomal dominant distal renal tubular acidosis, Autosomal dominant distal RTA, Autosomal recessive distal renal tubular acidosis, Autosomal recessive distal RTA, Autosomal recessive proximal renal tubular acidosis, Autosomal recessive proximal RTA, Bartter, Bartter syndrome, Bartter Syndrome type 1, Bartter Syndrome type 3 Gitelman Syndrome, Bartter Syndrome Type 4, Familial hypocalciuric hypercalcaemia, Familial hypercalciuric hypocalcaemia, Proximal renal tubular acidosis type II, Proximal RTA type II, Distal renal tubular acidosis type I, Distal RTA type I, Dent and Lowe, Dent & Lowe, Dent disease, Dent's disease, Lowe syndrome, Oculocerebrorenal syndrome of Lowe, Oculocerebrorenal syndrome, OCRL
	3187	Familial hypomagnesaemia	
	2901	Primary Fanconi syndrome	
	3156	Pseudohypoaldosteronism type 2 (Gordon syndrome)	
	3156	Pseudohypoaldosteronism type 2 (Gordon syndrome)	
	2901	Primary Fanconi syndrome	
	3085	Bartter syndrome	
	2993	Hypophosphataemic rickets autosomal recessive (AR)	
	2901	Primary Fanconi syndrome	
	3102	Liddle syndrome	
	3044	Nephrogenic diabetes insipidus	
	3156	Pseudohypoaldosteronism type 2 (Gordon syndrome)	
	3037	Distal renal tubular acidosis with sensorineural deafness - gene mutations	
	3141	Pseudohypoaldosteronism type 1	
	2986	Hypophosphataemic rickets X-linked (XL)	
	3085	Bartter syndrome	
	3156	Pseudohypoaldosteronism type 2 (Gordon syndrome)	
	3092	Gitelman syndrome	
	3085	Bartter syndrome	
	3187	Familial hypomagnesaemia	
	3160	Familial hypocalciuric hypercalcaemia	
	2972	Inherited renal glycosuria	
	3173	Familial hypercalciuric hypocalcaemia	
	3028	Distal renal tubular acidosis (RTA) - type I	

	3016	Proximal renal tubular acidosis (RTA) - type II	
	2917	Tubular disorder as part of inherited metabolic diseases	
	3156	Pseudohypoaldosteronism type 2 (Gordon syndrome)	
	2929	Dent disease	
	2938	Lowe syndrome (oculocerebrorenal syndrome)	
**ANCA positive vasculitis**	1383	Systemic vasculitis - ANCA negative - histologically proven	MPO Vasculitis, MPO-Vasculitis, PR3 vasculitis, PR3-vasculitis, Granulomatosis with polyangiitis, Polyangiitis, GPA, Wegener, Wegener's, Eosinophilic granulomatosis with polyangiitis, EGPA, Churg Strauss, ANCA Vasculitis
	1429	Microscopic polyangiitis - histologically proven	
	3852	Systemic vasculitis - ANCA positive - histologically proven	
	1401	Granulomatosis with polyangiitis - no histology	
	1417	Granulomatosis with polyangiitis - histologically proven	
	1396	Systemic vasculitis - ANCA positive - no histology	
	1438	Churg-Strauss syndrome - no histology	
	1440	Churg-Strauss syndrome - histologically proven	
**Anti-GBM disease**	1472	Anti-Glomerular basement membrane (GBM) disease / Goodpasture's syndrome - histologically proven	Anti GBM disease, Anti-GBM disease, Anti GBM, Anti-GBM, Anti Glomerular basement membrane, Anti-Glomerular Basement,
	1464	Anti-Glomerular basement membrane (GBM) disease / Goodpasture's syndrome - no histology	
**Other Vasculitides**	3847	Systemic vasculitis - ANCA negative - no histology	Vasculitis, Small vessel vasculitis, IgA vasculitis, IgAV, Henoch Schonlein purpura, Henoch Schonlein, Henoch Schönlein, HSP, Cryoglobulinaemic vasculitis, Classical Polyarteritis Nodosa, Polyarteritis Nodosa, PAN, Kawasaki disease, Kawasaki, Kawasaki's, Giant cell arteritis, GCA, Takayasu's arteritis, Takayasu, Takayasu's, Behcet's disease, Behcet's, Behcet, Cogan's syndrome, Cogan's, Cogan, Isolated aortitis, Primary cerebral aortitis
	1515	Henoch-Schönlein purpura / nephritis - histologically proven	
	1504	Henoch-Schönlein purpura / nephritis - no histology	
	1455	Polyarteritis nodosa	

Supplementary Table 5: Ethnicity and IMD Quintile*, stratified by current age

Ethnicity	White n	(%)	Mixed n	(%)	Asian n	(%)	Black n	(%)	Other n	(%)	P-value**
Paediatric	1103	(74.7)	49	(3.3)	255	(17.3)	48	(3.3)	21	(1.4)	<0.0001
Adults	17185	(87.6)	149	(0.8)	1471	(7.5)	570	(2.9)	239	(1.2)	

IMD Quintile	1- Most deprived n	(%)	2 n	(%)	3 n	(%)	4 n	(%)	5- Least deprived n	(%)	P-value**
Paediatric	580	(30.3)	318	(16.6)	342	(17.9)	341	(17.8)	334	(17.4)	<0.0001
Adults	4126	(17.3)	4575	(19.2)	4883	(20.5)	5082	(21.4)	5125	(21.5)	

*Complete case analysis **Chi^2 test

Supplementary Table 6: Ethnic and IMD Quintile distribution of children (aged ≤18 years) in England* compared to the children in RaDaR

	Children in the English population		Children in RaDaR population		P-value**
	n	(%)	N	(%)	
**Ethnicity**					
White	9495175	(79)	1008	(73)	<0.0001
Mixed	603396	(5)	48	(4)	
Asian	1155283	(10)	249	(18)	
Black	567083	(5)	47	(3)	
Other	149430	(1)	20	(1)	
Total	11970367	(100)	1372	(100)	
**IMD Quintile**					
1-Most deprived	3151456	(24)	548	(31)	<0.0001
2	2753699	(21)	298	(17)	
3	2514215	(19)	320	(18)	
4	2402419	(18)	318	(18)	
5- Least deprived	2460532	(19)	305	(17)	
Total	13282321	(100)	1,789	(100)	

*Office of National Statistics 2011 census data **Chi2 test

"Asian" comprised the following ONS categories:
"Asian or Asian British - Bangladeshi"
"Asian or Asian British - Indian"
"Asian or Asian British - Pakistani"
"Other Asian Background"
"Chinese"

"Black" comprised the following ONS categories:
"Black African"
"Black Carribean"
"Other Black Background"

"Mixed" comprised the following ONS categories:
"Mixed - White and Asian"
"Mixed - White and Black African"
"Mixed - White and Black Caribbean"
"Other Mixed Background"

"Other" comprised the following ONS category:
Other Ethnic Background

"White" comprised the following ONS categories:
"White - British"
"Other White Background"
"White - Irish"

Supplementary Table 7: Monogenic and Non monogenic disorders, stratified by ethnicity

		Monogenic		Non monogenic		P-value**
		n	(%)	n	(%)	
**All**	Paediatric	575	(29.7)	1360	(70.3)	<0.0001
	Adults	9352	(38.9)	14717	(61.1)	
**White**	Paediatric	311	(28.2)	793	(71.8)	<0.0001
	Adults	6575	(38.1)	10693	(61.9)	
**Mixed**	Paediatric	11	(22.4)	38	(77.6)	0.029
	Adults	59	(39.6)	90	(60.4)	
**Asian**	Paediatric	61	(23.9)	194	(76.1)	0.730
	Adults	339	(22.9)	1139	(77.1)	
**Black**	Paediatric	8	(16.7)	40	(83.3)	0.035
	Adults	179	(31.2)	395	(68.8)	
**Other**	Paediatric	7	(33.3)	14	(66.7)	0.885
	Adults	76	(31.8)	163	(68.2)	

	Monogenic		Non monogenic		P-value
	n	(%)	N	(%)	
White	6886	(90.3)	11486	(84.7)	<0.0001
Mixed	70	(0.9)	128	(0.9)	
Asian	400	(5.2)	1333	(9.8)	
Black	187	(2.5)	435	(3.2)	
Other	83	(1.1)	177	(1.3)	

**Chi^2 test . Excluding patients with ethnicity data missing. Patients with two diagnoses are included for each diagnosis.

		1- Most deprived		2		3		4		5- Least deprived		P-value*
		n	(%)	n	(%)	n	(%)	n	(%)	n	(%)	
**All**	Monogenic	1671	(16.9)	1878	(19.0)	2063	(20.9)	2122	(21.5)	2129	(21.6)	<0.0001
	Not Monogenic	3066	(19.2)	3039	(19.0)	3185	(19.9)	3320	(20.8)	3357	(21.0)	
**White**	Monogenic	1099	(16.0)	1213	(17.7)	1445	(21.1)	1524	(22.3)	1567	(22.9)	0.429
	Not Monogenic	1948	(17.1)	2003	(17.6)	2331	(20.5)	2534	(22.2)	2578	(22.6)	
**Mixed**	Monogenic	14	(20.0)	20	(28.6)	12	(17.1)	12	(17.1)	12	(17.1)	0.718
	Not Monogenic	32	(25.0)	31	(24.2)	24	(18.8)	15	(11.7)	26	(20.3)	
**Asian**	Monogenic	145	(36.5)	83	(20.9)	64	(16.1)	56	(14.1)	49	(12.3)	0.826
	Not Monogenic	451	(33.9)	312	(23.5)	214	(16.1)	185	(13.9)	168	(12.6)	
**Black**	Monogenic	77	(41.4)	48	(25.8)	32	(17.2)	16	(8.6)	13	(7.0)	0.254
	Not Monogenic	158	(36.3)	150	(34.5)	74	(17.0)	32	(7.4)	21	(4.8)	
**Other**	Monogenic	12	(14.5)	25	(30.1)	21	(25.3)	13	(15.7)	12	(14.5)	0.010
	Not Monogenic	57	(32.2)	42	(23.7)	27	(15.3)	17	(9.6)	34	(19.2)	

Supplementary Table 8: Monogenic and Non monogenic disorders, stratified by age category* and ethnicity

*Age on July 25th 2022 **Chi^2 test. Excluding patients with ethnicity data missing. Patients with two diagnoses are included for each diagnosis.

Supplementary Table 9: Monogenic and Non monogenic disorders, stratified by Ethnicity and IMD Quintile

*Chi^2 test. Excluding patients with ethnicity and IMD Quintile data missing. Patients with two diagnoses are included for each diagnosis.

Supplementary Table 10: Socioeconomic status (IMD Quintile) of RaDaR participants, stratified by mode of inheritance and age category*

*age on 25th July 2022 **Chi^2 test

		1- Most deprived		2		3		4		5- Least deprived		P-value**
		**n**	**(%)**	**n**	**(%)**	**n**	**(%)**	**n**	**(%)**	**n**	**(%)**	
**Autosomal dominant**		1263	(16.1)	1485	(18.9)	1643	(20.9)	1721	(21.9)	1747	(22.2)	<0.0001
**Autosomal Recessive/X-linked**		408	(20.4)	393	(19.6)	420	(21.0)	401	(20.0)	382	(19.1)	
**Mostly non monogenic**		3066	(19.2)	3039	(19.0)	3185	(19.9)	3320	(20.8)	3357	(21.0)	
**Autosomal Dominant**	Paediatric	62	(28.2)	38	(17.3)	33	(15.0)	42	(19.1)	45	(20.5)	<0.0001
	Adults	1201	(15.7)	1447	(18.9)	1610	(21.1)	1679	(22.0)	1702	(22.3)	
**Autosomal Recessive/X-linked**	Paediatric	112	(32.0)	59	(16.9)	65	(18.6)	63	(18.0)	51	(14.6)	<0.0001
	Adults	296	(17.9)	334	(20.2)	355	(21.5)	338	(20.4)	331	(20.0)	
**Mostly non monogenic**	Paediatric	406	(30.2)	222	(16.5)	244	(18.1)	236	(17.5)	238	(17.7)	<0.0001
	Adults	2660	(18.2)	2817	(19.3)	2941	(20.1)	3084	(21.1)	3119	(21.3)	

Supplementary Table 11: IMD Quintile, stratified by current age and Ethnicity

		1- Most deprived		2		3		4		5- Least deprived		p-value*
		n	(%)	n	(%)	n	(%)	n	(%)	n	(%)	
White	Paediatric	272	(24.8)	183	(16.7)	207	(18.9)	213	(19.4)	221	(20.2)	p<0.0001
	Adults	2758	(16.2)	3017	(17.7)	3550	(20.8)	3831	(22.5)	3906	(22.9)	
Mixed	Paediatric	10	(33.3)	10	(33.3)	≤6	NR**	≤6	NR	10	(33.3)	p=0.626
	Adults	33	(22.1)	39	(26.2)	30	(20.1)	21	(14.1)	26	(17.4)	
Asian	Paediatric	136	(53.5)	46	(18.1)	26	(10.2)	25	(9.8)	21	(8.3)	p<0.0001
	Adults	456	(31.1)	347	(23.7)	251	(17.1)	216	(14.7)	196	(13.4)	
Black	Paediatric	25	(55.5)	10	(22.2)	10	(22.2)	≤6	NR	≤6	NR	p=0.489
	Adults	208	(36.6)	186	(32.7)	98	(17.2)	45	(7.9)	32	(5.6)	
Other	Paediatric	10	(50.0)	10	(50.0)	≤6	NR	≤6	NR	0	(0.0)	
	Adults	59	(24.7)	59	(24.7)	46	(19.2)	29	(12.1)	46	(19.2)	0.017

*Fishers exact test **NR- Not reported; cells with fewer than 6 patients not reported due to risk of re-identification. Where cells are not reported, corresponding cell values are rounded to nearest 5

Supplementary Table 12: Socioeconomic status (IMD Quintile), stratified by monogenic and non monogenic disorders and age category*

		**1- Most deprived**		**2**		**3**		**4**		**5- Least deprived**		**P-value****
		n	(%)	n	(%)	n	(%)	n	(%)	n	(%)	
**Paediatric**	Monogenic	174	(30.5)	97	(17.0)	98	(17.2)	105	(18.4)	96	(16.8)	0.961
	Non monogenic	406	(30.2)	222	(16.5)	244	(18.1)	236	(17.5)	238	(17.7)	
**Adults**	Monogenic	1497	(16.1)	1781	(19.2)	1965	(21.1)	2017	(21.7)	2033	(21.9)	0.001
	Non monogenic	2660	(18.2)	2817	(19.3)	2941	(20.1)	3084	(21.1)	3119	(21.3)	
**Monogenic**	Paediatric	174	(30.5)	97	(17.0)	98	(17.2)	105	(18.4)	96	(16.8)	<0.0001
	Adults	1497	(16.1)	1781	(19.2)	1965	(21.1)	2017	(21.7)	2033	(21.9)	
**Non monogenic**	Paediatric	406	(30.2)	222	(16.5)	244	(18.1)	236	(17.5)	238	(17.7)	<0.0001
	Adults	2660	(18.2)	2817	(19.3)	2941	(20.1)	3084	(21.1)	3119	(21.3)	

*Age on July 25th 2022 **Chi^2 test. Excluding patients with ethnicity data missing. Patients with two diagnoses are included for each diagnosis.

Supplementary Table 13: Number and percentage of rare disease diagnoses for each disorder within UKRR and RaDaR KRT recipients

**Rare Disease Group**	**RaDaR KRT population**		**UKRR rare disease KRT population**	
	**n**	**(%)**	**n**	**(%)**
ADPKD	2986	(35.1)	6880	(32.4)
ADTKD	88	(1.0)	318	(1.5)
ARPKD/NPHP	99	(1.2)	237	(1.1)
Alport Syndrome	344	(4.0)	716	(3.4)
Cystinosis	87	(1.0)	133	(0.6)
Cystinuria	7	(0.1)	11	(0.1)
Hyperoxaluria	36	(0.4)	76	(0.4)
INS	666	(7.8)	2324	(11)
IgA Nephropathy	2109	(24.8)	5772	(27.2)
MGRS	76	(0.9)	275	(1.3)
MPGN/C3GN	476	(5.6)	825	(3.9)
Membranous Nephropathy	425	(5.0)	929	(4.4)
Renal cancer inherited	≤6	(0.0)	15	(0.1)
Retroperitoneal Fibrosis	15	(0.2)	29	(0.1)
STEC HUS	28	(0.3)	60	(0.3)
Tuberous Sclerosis	21	(0.2)	33	(0.2)
Tubulopathies	28	(0.3)	72	(0.3)
Vasculitis	904	(10.6)	2201	(10.4)
aHUS	122	(1.4)	297	(1.4)
Total Rare Disease KRT Recipients	8517	(100)	21203	(100)

* KRT = Kidney replacement therapy, UKRR = UK Renal Registry, denominator being the total number of KRT recipients with an eligible rare disease diagnosis

Supplementary Figure 1: Comparison of ethnicity in each Rare Disease Group

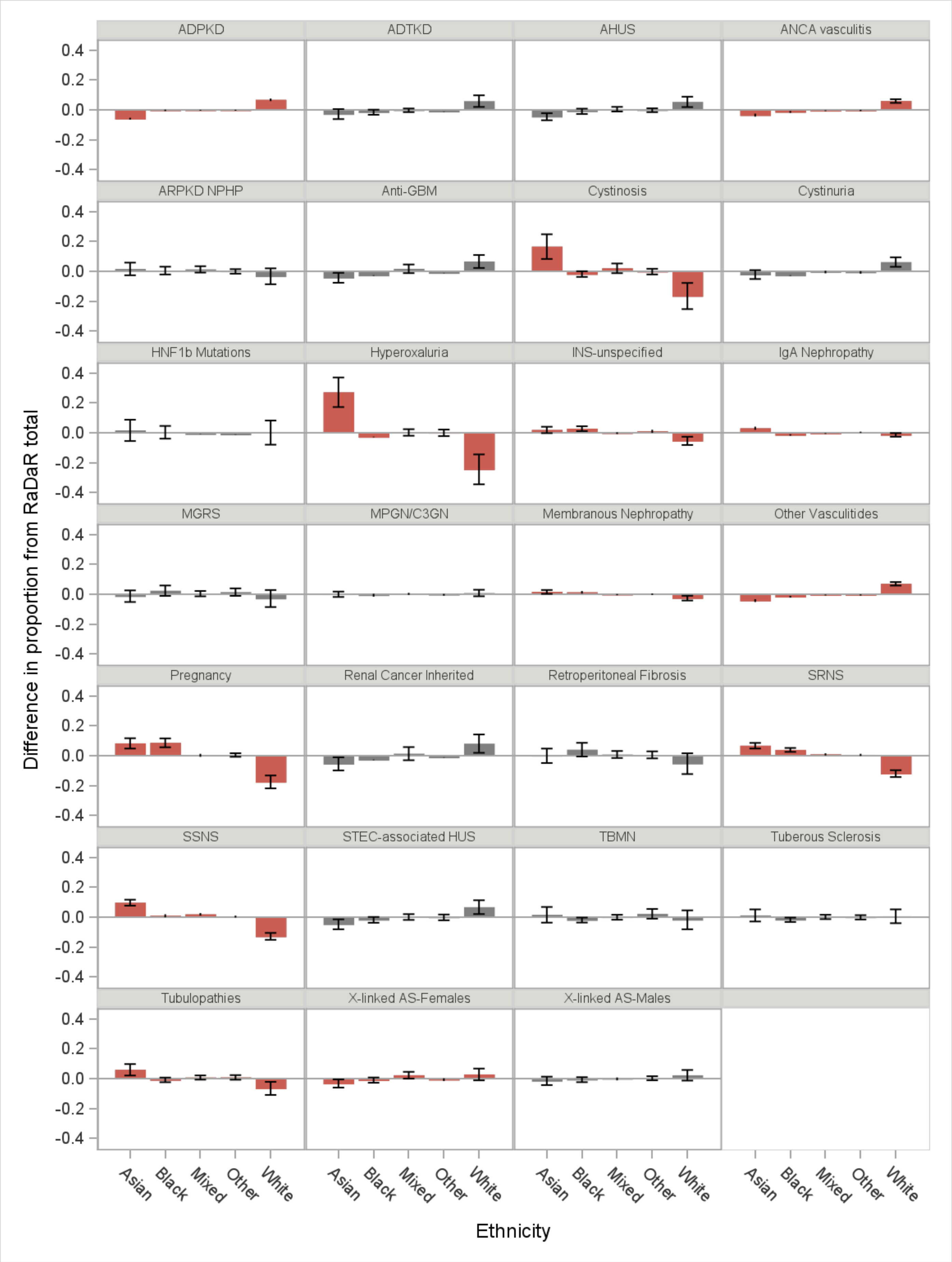

Proportion of patients of each ethnicity for each RDG compared to the IMD Quintile breakdown of RaDaR. RDGs highlighted in red have Chi-square p-value < 0.00185 (5% significance level with Bonferroni correction). Error bars display 95% CI using the two-sample test of proportions (Z-test).

Supplementary Figure 2: Comparison of ethnicity in each Rare Disease Group to total ethnicity breakdown of RaDaR, stratified by current a) paediatric b) adult patients

a)

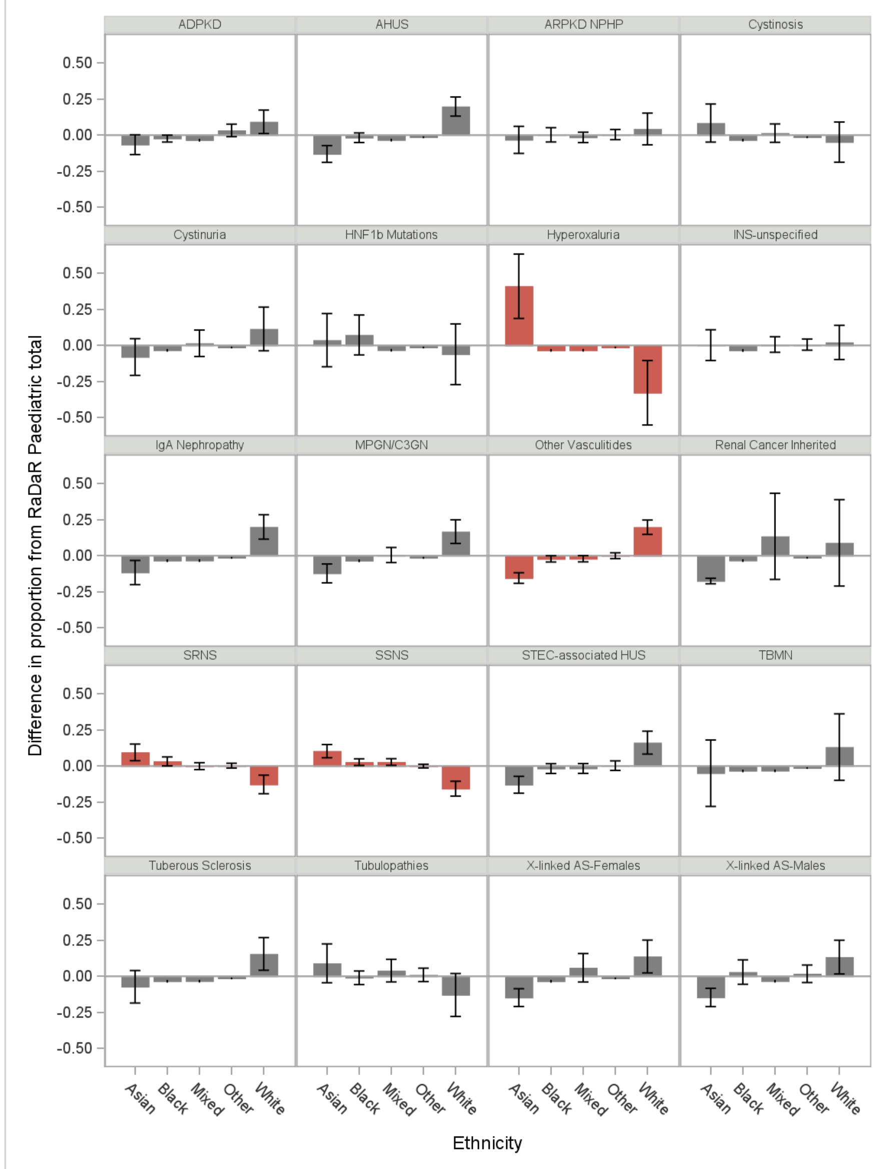

b)

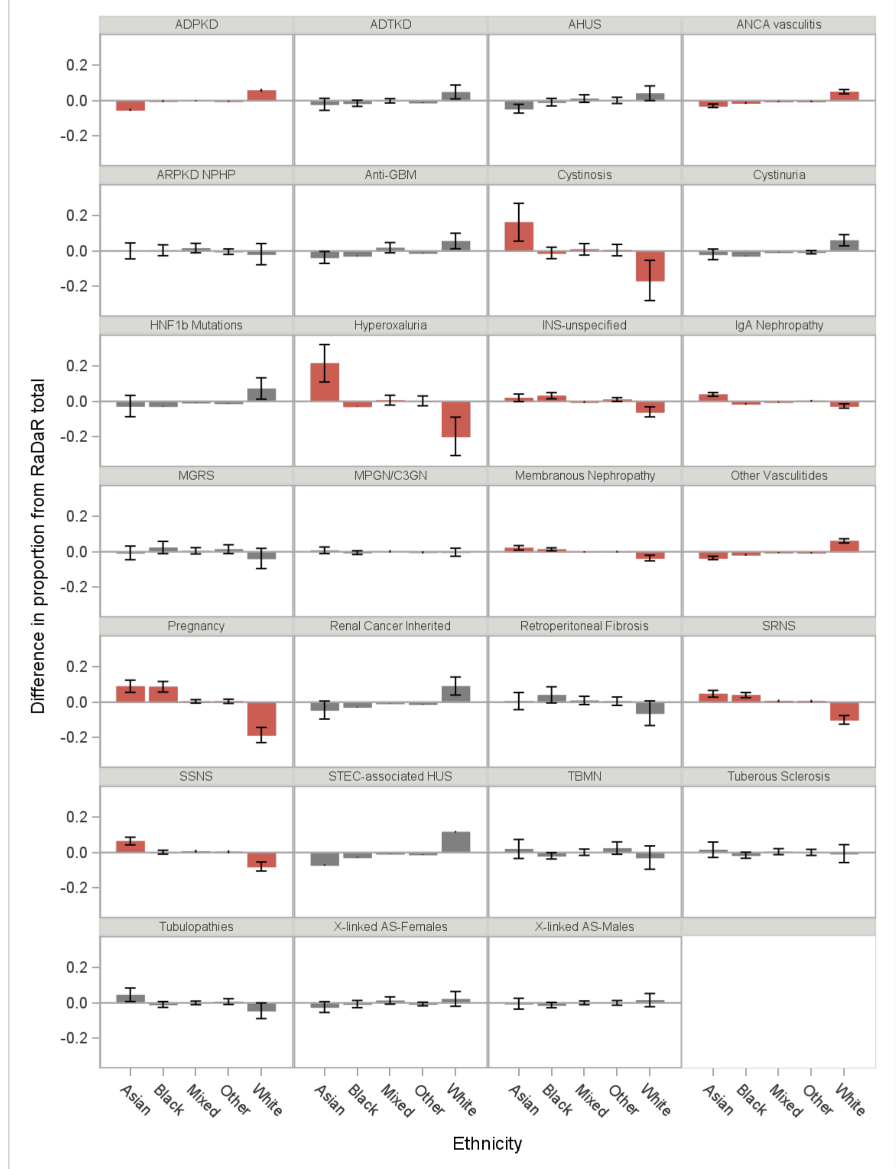

Proportion of patients of each ethnicity for each RDG compared to the IMD Quintile breakdown of RaDaR. RDGs highlighted in red have Chi-square p-value < 0.0025 or <0.00185 for paediatrics and adults respectively (5% significance level with Bonferroni correction). Error bars display 95% CI using the two-sample test of proportions (Z-test). Age group on 25th July 2022. RDGs with fewer than 10 patients excluded.

Supplementary Figure 3: Comparison of IMD Quintile in each Rare Disease Group to total IMD Quintile distribution of RaDaR

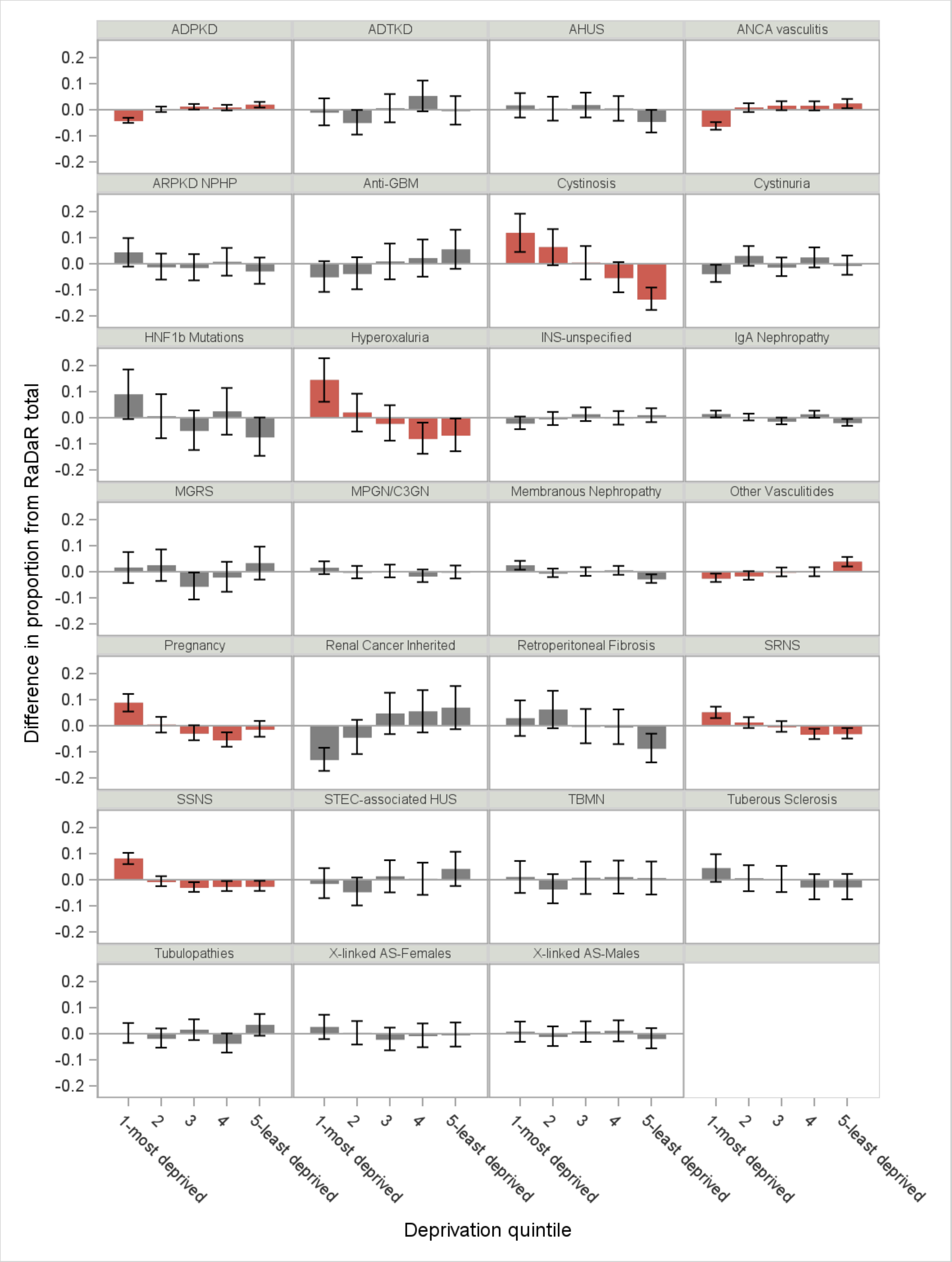

Proportion of patients in each IMD Quintile for each RDG compared to the IMD Quintile breakdown of RaDaR. RDGs highlighted in red have Chi-square p-value < 0.00185 (5% significance level with Bonferroni correction). Error bars display 95% CI using the two-sample test of proportions (Z-test). Age group on 25th July 2022. RDGs with fewer than 10 patients excluded.

Supplementary Figure 4: Comparison of IMD Quintile in each Rare Disease Group to total IMD Quintile distribution of RaDaR, stratified by current a) paediatric b) adult patients

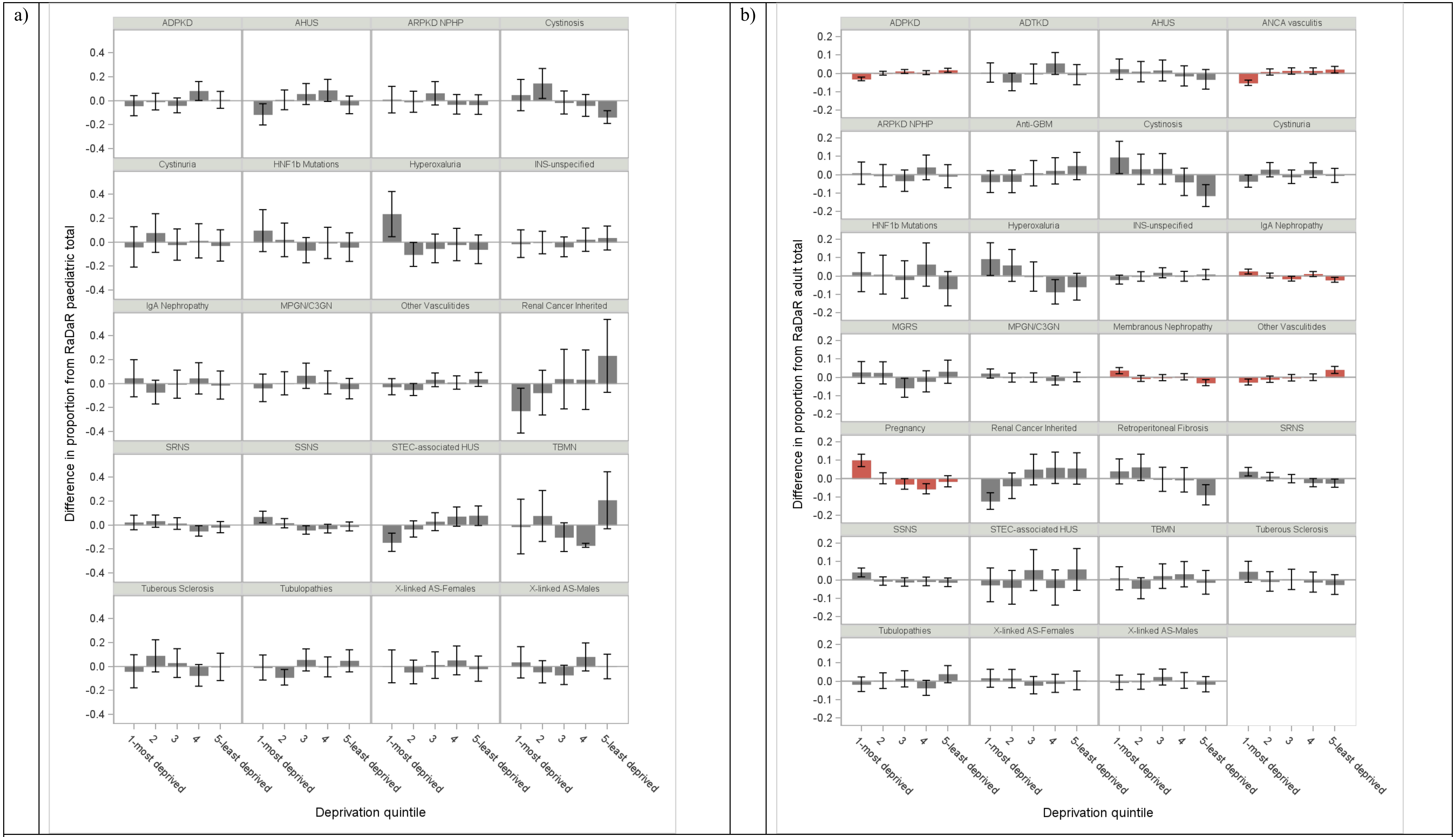

Proportion of patients in each IMD Quintile for each RDG compared to the IMD Quintile breakdown of RaDaR. RDGs highlighted in red have Chi-square p-value < 0.0025 or <0.00185 for paediatrics and adults respectively (5% significance level with Bonferroni correction). Error bars display 95% CI using the two-sample test of proportions (Z-test). Age group on 25th July 2022. RDGs with fewer than 10 patients excluded.

Supplementary Figure 5: Ethnicity of English RaDaR patients with ADPKD compared to English census

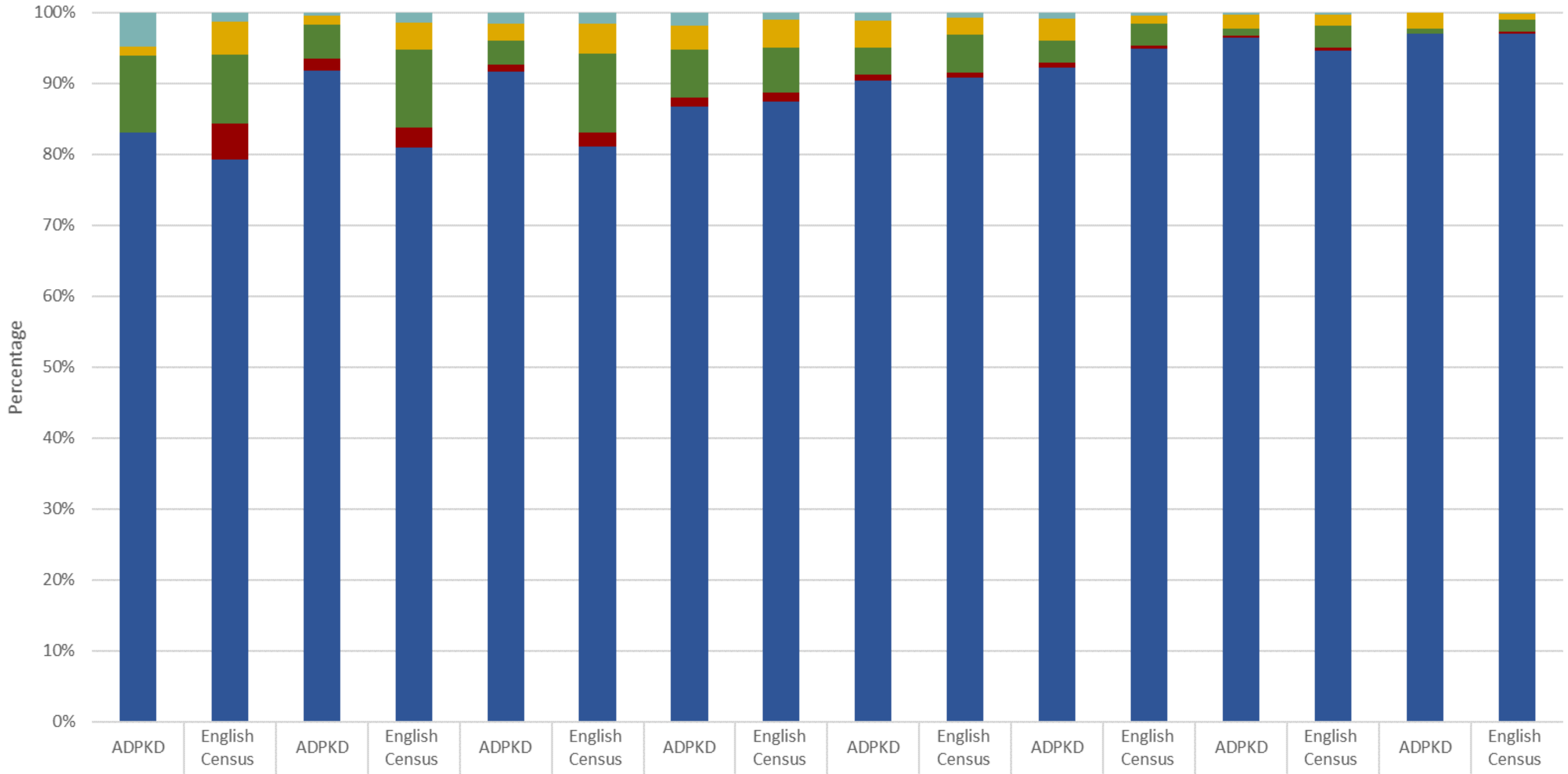

	0 -17 years		18-29 years		30-39 years		40-49 years		50-59 years		60-69 years		70-79 years		>=80 years	
	ADPKD	English Census	ADPKD	English Census	ADPKD	English Census	ADPKD	English Census	ADPKD	English Census	ADPKD	English Census	ADPKD	English Census	ADPKD	English Census
Other	5%	1%	0%	1%	2%	2%	2%	1%	1%	1%	1%	0%	0%	0%	0%	0%
Black	1%	5%	1%	4%	2%	4%	3%	4%	4%	2%	3%	1%	2%	2%	2%	1%
Asian	11%	10%	5%	11%	3%	11%	7%	6%	4%	5%	3%	3%	1%	3%	1%	2%
Mixed	0%	5%	2%	3%	1%	2%	1%	1%	1%	1%	1%	0%	0%	0%	0%	0%
White	83%	79%	92%	81%	92%	81%	87%	87%	90%	91%	92%	95%	97%	95%	97%	97%
P value*	0.006		0.001		<0.0001		0.074		<0.0001		<0.0001		0.003		0.387	

*Fishers exact test

Supplementary Table 14: Recruiting centres

Numbers recruited per centre as of 25/07/2022

Centre	Number	Centre	Number	Centre	Number
Aberdeen	2	Dudley, Russells Hall	18	Newcastle Freeman and Victoria	899
Airedale	22	Dumfries & Galloway	13	Norfolk and Norwich	326
Altnagelvin Hospital, Derry	21	Dundee Ninewells	140	North Tees, Stockton	33
Antrim	29	Edinburgh	127	Nottingham Adults	856
Ashford & St Peters	12	Exeter	577	Nottingham Children's	219
Bangor	14	Glasgow Children's	91	Oxford Churchill	1171
Basildon	217	Glasgow Queen Elizabeth	606	Oxford Women's	85
Bath Royal United	8	Gloucester	257	Peterborough	3
Belfast Children’s	40	Gt Yarmouth - James Paget	21	Plymouth	438
Belfast Ulster	102	Hartlepool	10	Portsmouth	77
Birkenhead Arrowe Park	104	Hereford	100	Preston	539
Birmingham Children’s	585	Huddersfield	106	Reading	127
Birmingham Heartlands	241	Hull	275	Romford Queens Hospital	18
Birmingham Queen Elizabeth	674	Inverness	158	Salford Royal	707
Birmingham Sandwell	4	Ipswich	273	Sheffield Northern General	475
Birmingham Women’s	158	Kilmarnock (Crosshouse)	41	Shrewsbury & Telford	426
Blackburn	19	Kings Lynn	39	Southampton Children’s	177
Bradford (St Luke’s)	224	Leeds Children’s	261	Southend	219
Brighton	298	Leeds St James'	223	St Heliers	847
Bristol Children's	108	Leicester	719	Stevenage (Lister)	876
Bristol Southmead	190	Lincoln County Hospital	13	Stockport	32
Burton-on-Trent	36	Liverpool Aintree	91	Stoke	354
Bury St Edmunds	10	Liverpool Alder Hey	165	Sunderland	270
Cambridge	762	Liverpool Royal	642	Swansea	61
Canterbury	426	London – Barts	626	Swindon Great Western	8
Cardiff	785	London - Evelina	10	Tameside	4
Chelmsford (Broomfield)	202	London – Great Ormond Street	175	Taunton Musgrove Park	6
Chester	5	London – Guys	1336	Torbay	33
Colchester	104	London – Imperial	232	Truro Royal Cornwall	314
Coventry	699	London - Kings	679	Walsall Manor	14
Cumberland Infirmary	126	London - Royal Free	1316	Warrington	5
Daisy Hill, Newry	38	London – St George’s	563	West Cumberland	48
Darlington	88	Macclesfield	11	Wolverhampton	244
Dartford	28	Manchester Adults	1170	Worcestershire Royal	29
Derby	487	Manchester Children's	365	York	521
Doncaster	22	Middlesbrough	388		

RaDaR Inclusion and Exclusion Criteria

Supplementary Table 15: RaDaR Eligibility Criteria

Diagnosis	Cohort	Inclusion Criteria	Exclusion Criteria	Date of Diagnosis
**Adenine Phosphoribosyltransferase Deficiency (APRT-D)**	APRT Deficiency	APRT Deficiency confirmed<br><br>Abolished APRT enzyme activity or confirmed disease-causing mutation	None, if APRT Deficiency not confirmed	Date that clinical diagnosis was first made
**Alport Syndrome and Type IV collagenopathies**	Alport	Alport Syndrome definite or probable<br>Alport carrier definite or probable<br>Female heterozygote for X-linked Alport Syndrome (COL4A5)<br>Heterozygote for autosomal Alport Syndrome (COL4A3, COL4A4)<br>Thin basement membrane nephropathy	None stated	Date that clinical diagnosis was first made
**APOL1 disease, suspected or confirmed**	CKD-Africa Genes	People of African or Afro-Caribbean ancestry with CKD (KDIGO definition), >18 years including: Focal segmental glomerulosclerosis (primary or secondary) on renal biopsy; Non-diabetic and non-immunological kidney disease with no other confirmed cause	None stated	Date that clinical diagnosis was first made
**Autoimmune distal renal tubular acidosis**	Tubulopathy	Autoimmune distal renal tubular acidosis	None stated	Date that clinical diagnosis was first made
**Autosomal dominant distal renal tubular acidosis**	Tubulopathy	Autosomal dominant distal renal tubular acidosis<br>Genetically confirmed heterozygous pathogenic variant in SLC4A1	None stated	Date that clinical diagnosis was first made
**Autosomal recessive distal renal tubular acidosis**	Tubulopathy	Autosomal recessive distal renal tubular acidosis<br><br>Genetically confirmed homozygous pathogenic variant in ATP6V0A4, ATP6V1B1 or FOXI1	None stated	Date that clinical diagnosis was first made

RaDaR Inclusion and Exclusion Criteria

Diagnosis	Cohort	Inclusion Criteria	Exclusion Criteria	Date of Diagnosis
**Autosomal recessive proximal renal tubular acidosis**	Tubulopathy	Autosomal recessive proximal renal tubular acidosis with ocular abnormalities and intellectual disability<br><br>Genetically confirmed homozygous pathogenic variant in SLC4A4	None stated	Date that clinical diagnosis was first made

RaDaR Inclusion and Exclusion Criteria

Diagnosis	Cohort	Inclusion Criteria	Exclusion Criteria	Date of Diagnosis
**Bartter Syndrome types 1 and 2**	Tubulopathy	Bartter Syndrome, infantile onset<br><br>Hypokalaemic alkalosis, infantile onset without hypertension<br><br>Hypokalaemic alkalosis, infantile onset with raised renin	Acidosis<br><br>Persistent Hyperkalaemia	Date that clinical diagnosis was first made
**Bartter Syndrome type 3 Gitelman Syndrome**	Tubulopathy	Bartter Syndrome type 3<br><br>Gitelman Syndrome<br><br>Hypokalaemic alkalosis with hypomagnesaemia<br><br>Hypokalaemic alkalosis with raised renin<br><br>Hypokalaemic alkalosis without hypertension	Acidosis<br><br>Hyperkalaemia	Date that clinical diagnosis was first made
**Bartter Syndrome Type 4**	Tubulopathy	Bartter Syndrome, infantile onset with deafness<br><br>Hypokalaemic alkalosis, infantile onset without hypertension with deafness<br><br>Hypokalaemic alkalosis, infantile onset with raised renin, with deafness	Acidosis<br><br>Persistent Hyperkalaemia	Date that clinical diagnosis was first made
**BK Nephropathy**	BK Nephropathy	Significant BK viraemia, with polymerase chain reaction (PCR) greater than or equal to 10 log 4 copies per ml.<br><br>A confirmatory biopsy is **not** required.	None stated	Date that PCR first equalled or exceeded 10 log 4

RaDaR Inclusion and Exclusion Criteria

Diagnosis	Cohort	Inclusion Criteria	Exclusion Criteria	Date of Diagnosis
**Calciphylaxis**	Calciphylaxis	Any patient with a diagnosis of clinical diagnosis of Calciphylaxis; tissue diagnosis not required	None stated	Date that the diagnosis was made by a nephrologist or dermatologist
**Cystinosis (Nephropathic Cystinosis)**	Cystinosis	Cystinosis	None stated	Date that biochemical testing first showed an elevated level of white blood cell cysteine
**Cystinuria**	Cystinuria	Biochemically proven cystine kidney stone<br><br>Urinary cystine level > 3X reference range of the laboratory it was taken in<br><br>Cystine crystals in the urine (biochemically proven)	Another cause of proximal tubular dysfunction accounting for the raised cystine level e.g. Fanconi's syndrome	Date that any of the inclusion criteria first occurred
**Dent Disease**	Dent & Lowe	Dent Disease	None stated	Date that the clinical label of Dent Disease was first applied
**Dominant hypophosphatemia with nephrolithiasis or osteoporosis**	Tubulopathy	Dominant hypophosphatemia with nephrolithiasis or osteoporosis<br><br>Genetically confirmed heterozygous pathogenic variant in SLC34A1, SLC9A3R1, SLC34A3	None stated	Date that clinical diagnosis was first made
**Drug induced Fanconi syndrome**	Tubulopathy	Drug induced Fanconi syndrome	None stated	Date that clinical diagnosis was first made
**Drug induced hypomagnesemia**	Tubulopathy	Drug induced hypomagnesemia	None stated	Date that clinical diagnosis was first made

RaDaR Inclusion and Exclusion Criteria

Diagnosis	Cohort	Inclusion Criteria	Exclusion Criteria	Date of Diagnosis
**Drug induced Nephrogenic Diabetes Insipidus**	Tubulopathy	Drug induced Nephrogenic Diabetes Insipidus	None stated	Date that clinical diagnosis was first made
**EAST syndrome (Epilepsy, Ataxia, Sensorineural deafness, Tubulopathy)**	Tubulopathy	Gitelman/Bartter-type syndrome in childhood with epilepsy /ataxia	Normal CNS examination	Date that clinical diagnosis was first made
**End stage kidney disease of unknown cause**	CKD-Africa Genes	People of African or Afro-Caribbean ancestry with CKD (KDIGO definition), >18 years	Known cause of kidney disease identified (unless Sickle cell Nephropathy or APOL1 disease)	Date that clinical diagnosis was first made
**Fabry Disease**	Fabry	Confirmed diagnosis of Fabry Disease	None stated	Date that genetic diagnosis was made and/or, for males, the date that low alpha gal levels were first recorded
**Familial Hypomagnesaemia with hypercalciuria and nephrocalcinosis CLDN16/19**	Tubulopathy	Familial Hypomagnesaemia with Hypercalciuria and Nephrocalcinosis<br><br>Genetically confirmed homozygous pathogenic variant in CLDN 16/19	None stated	Date that clinical diagnosis was first made
**Familial primary hypomagnesemia with hypocalcuria FXYD2**	Tubulopathy	Familial primary hypomagnesemia with hypocalciuria<br>Genetically confirmed homozygous pathogenic variant in FXYD2	None stated	Date that clinical diagnosis was first made
**Familial primary hypomagnesemia with normocalcuria EGF**	Tubulopathy	Familial primary hypomagnesemia with normocalcuria<br><br>Genetically confirmed homozygous pathogenic variant in EGF	None stated	Date that clinical diagnosis was first made
**Familial renal glucosuria**	Tubulopathy	Familial renal glucosuria	None stated	Date that clinical diagnosis

Diagnosis	Cohort	Inclusion Criteria	Exclusion Criteria	Date of Diagnosis
**SLC5A2**		Genetically confirmed homozygous pathogenic variant in SLC5A2		was first made

RaDaR Inclusion and Exclusion Criteria

Diagnosis	Cohort	Inclusion Criteria	Exclusion Criteria	Date of Diagnosis
**Fanconi Renotubular syndrome 1 (FRTS1)**	Tubulopathy	Fanconi Renotubular syndrome 1	None stated	Date that clinical diagnosis was first made
**Fanconi Renotubular syndrome 2 (FRTS2)**	Tubulopathy	Fanconi Renotubular syndrome 2<br><br>Genetically confirmed homozygous pathogenic variant in SLC34A1	None stated	Date that clinical diagnosis was first made
**Fanconi Renotubular syndrome 3 (FRTS3)**	Tubulopathy	Fanconi Renotubular syndrome 3<br><br>Genetically confirmed homozygous pathogenic variant in EHHADH	None stated	Date that clinical diagnosis was first made
**Fibromuscular Dysplasia**	Fibromuscular Dysplasia	Diagnosis of FMD established on radiological or histological grounds<br><br>FMD of any arterial bed	None stated	Date that FMD was diagnosed by radiological (or histological) methods
**Generalized pseudohypoaldosteronism type 1**	Tubulopathy	Generalized pseudohypoaldosteronism type 1<br><br>Genetically confirmed homozygous pathogenic variant in SCNN1A/ SCNN1B/SCNN1G	None stated	Date that clinical diagnosis was first made

RaDaR Inclusion and Exclusion Criteria

Diagnosis	Cohort	Inclusion Criteria	Exclusion Criteria	Date of Diagnosis
**Haemolytic Uraemic Syndrome - Atypical**	aHUS	Diarrhoea-negative HUS, includes congenital and familial HUS<br><br>Renal biopsy showing a TMA and/or the triad of microangiopathic haemolytic anaemia, thrombocytopenia, renal failure.	Shiga toxin associated HUS<br><br>Secondary causes:<br>• Drugs<br>• Infection (HIV, pneumonia, streptococcus)<br>• Transplantation (bone marrow, liver, lung, cardiac but not de-novo renal)<br>• Cobalamin deficiency<br>• SLE<br>• APL Ab syndrome<br>• Scleroderma<br>• ADAMTS13 antibodies or deficiency	Date of first presentation

RaDaR Inclusion and Exclusion Criteria

Diagnosis	Cohort	Inclusion Criteria	Exclusion Criteria	Date of Diagnosis
**Haemolytic Uraemic Syndrome-Shiga toxin (Verocytotoxin)-associated**	STEC-HUS	Acute kidney injury (AKI) with elevated creatinine for age and/or oligoanuria (urine output <0.5ml/kg/hr over 24hr period) with either:<br>• Microangiopathic haemolytic anaemia (MAHA) - defined as Hgb < 10mg/dl with fragmented RBCs<br>or<br>• Thrombocytopaenia - defined as platelet count less than 130, 000 x 10 9/l<br>and<br>• Occurring with Shiga-toxin producing E Coli (STEC) infection defined as:<br>• Positive STEC culture<br>• Positive PCR for Stx gene directly from a faecal specimen<br>• Positive antibodies to the lipopolysaccharide<br>• antigen of E. coli serogroups O157, O26, O103, O111 and O145	Septicaemia<br>Malignant hypertension<br>Primary vascular disease<br>Familial HUS not being part of the same	Date on which the STEC-HUS was suspected.
**Heavy metal induced Fanconi syndrome**	Tubulopathy	Heavy metal induced Fanconi syndrome	None stated	Date that clinical diagnosis was first made

RaDaR Inclusion and Exclusion Criteria

Diagnosis	Cohort	Inclusion Criteria	Exclusion Criteria	Date of Diagnosis
**Hepatocyte Nuclear Factor-1B mutation**	HNF1b	Hepatocyte nuclear factor-1B mutation<br><br>Renal cysts and diabetes (RCAD)<br><br>Inherited genetic diabetes type 2 (MODY 5).	None stated	Date of genetic diagnosis
**Hereditary renal hypouricemia**	Tubulopathy	Hereditary renal hypouricemia<br><br>Genetically confirmed homozygous pathogenic variant in SLC22A12, SLC2A9	None stated	Date of genetic diagnosis
**Hereditary hypophosphatemic rickets with hypercalciuria**	Tubulopathy	Hereditary hypophosphatemic rickets with hypercalciuria<br><br>Genetically confirmed homozygous pathogenic variant in SLC34A3	None stated	Date of genetic diagnosis
**Hyperoxaluria (Primary hyperoxaluria, Oxalosis)**	Hyperoxaluria	Primary Hyperoxaluria Type1<br><br>Primary Hyperoxaluria Type 2<br><br>Primary Hyperoxaluria Type 3<br><br>Primary Hyperoxaluria awaiting genetic confirmation (Urine oxalate excretion ≥ 0.8 mmol/1.73 m^2/24 hrs)<br><br>Primary Hyperoxaluria Unclassified<br><br>Primary Hyperoxaluria Unclassified but with systemic oxalate deposition	Secondary hyperoxaluria associated with gastrointestinal disease<br><br>Renal failure without systemic oxalate deposits	Date that definitive diagnosis by genetic confirmation with gene mutation was first made.<br><br>If in doubt use the earliest date that PH was suspected or the date when treatment was first introduced

RaDaR Inclusion and Exclusion Criteria

Diagnosis	Cohort	Inclusion Criteria	Exclusion Criteria	Date of Diagnosis
**Hypertensive kidney disease**	CKD-Africa Genes	People of African or Afro-Caribbean ancestry with CKD (KDIGO definition), >18 years	Known cause of Kidney disease	Date that clinical diagnosis was first made
**Hyperuricaemic Nephropathy (Primary/Familial Hyperuricaemic nephropathy)**<br><br>**Medullary cystic kidney disease**	ADTKD	Autosomal Dominant Tubulointerstitial Kidney Disease (ADTKD; previously known as FUAN)<br><br>Familial juvenile hyperuricaemic nephropathy<br><br>Familial gouty nephropathy<br><br>Familial urate nephropathy<br><br>Familial interstitial nephropathy<br><br>Uromodulin-associated nephropathy<br><br>Medullary cystic kidney disease (type I or II)	None stated	Date that genetic confirmation was received
**IgA Nephropathy**	IgA Nephropathy	Biopsy proven IgA Nephropathy plus proteinuria >0.5g/ day or eGFR<60ml/min	All forms of secondary IgA nephropathy, including Henoch Schonlein purpura	Date of renal biopsy
**Isolated autosomal dominant hypomagnesemia, Glaudemans type**	Tubulopathy	Isolated autosomal dominant hypomagnesemia<br><br>Genetically confirmed homozygous pathogenic variant in KCNA1	None stated	Date that clinical diagnosis was first made

RaDaR Inclusion and Exclusion Criteria

Diagnosis	Cohort	Inclusion Criteria	Exclusion Criteria	Date of Diagnosis
**Liddle syndrome**	Tubulopathy	Liddle syndrome<br><br>Hypertension with hypokalaemia, suppressed aldosterone<br><br>Hypertension with suppressed aldosterone<br><br>Autosomal dominant hypertension, suppressed aldosterone	Hyperaldosteronism	Date that clinical diagnosis was first made
**Lowe Syndrome**	Dent & Lowe	Lowe Syndrome	None Stated	Date that the clinical label of Lowe Syndrome was first applied

RaDaR Inclusion and Exclusion Criteria

Diagnosis	Cohort	Inclusion Criteria	Exclusion Criteria	Date of Diagnosis
**Membranoproliferative glomerulonephritis**<br><br>**Mesangiocapillary glomerulonephritis**<br><br>**Dense Deposit Disease**<br><br>**C3 Glomerulonephritis**<br><br>**C3 Glomerulopathy**	MPGN	Child or adult with histological finding of:<br><br>MPGN Type I<br><br>Dense Deposit Disease (morphological pattern may or may not be MPGN)<br><br>Other pattern of MPGN<br><br>C3 Glomerulonephritis (Characterised by C3 deposits in the absence of immunoglobulin with electron dense deposits (morphological pattern may or may not be MPGN)<br><br>Unclassified GN with capillary wall immune deposits	MPGN known to be secondary to:<br><br>Chronic bacterial infection<br><br>Hepatitis B or C infection<br><br>Malignancy<br><br>Systemic lupus erythematosus (by ACR criteria)	Date of biopsy
**Membranous Nephropathy**	Membranous Nephropathy	Membranous nephropathy confirmed by kidney histology	Lupus nephritis	Date of biopsy
**Mitochondrial Renal Disease**	Mitochondrial	Mitochondrial Disease **or** Mitochondrial Cytopathy	None Stated	Date that clinical diagnosis was first made

Diagnosis	Cohort	Inclusion Criteria	Exclusion Criteria	Date of Diagnosis
**Monoclonal Gammopathy of Renal Significance**	MGRS	Renal biopsy proven confirmation of:<br><br>• AH amyloidosis*<br>• AHL amyloidosis*<br>• AL amyloidosis*<br>• C3 glomerulonephritis with monoclonal gammopathy<br>• Crystalglobulinaemia<br>• Crystal-storing histiocytosis<br>• Fibrillary Glomerulonephritis<br>• Immunotactoid/Glomerulonephritis with Organised Microtubular Monoclonal Immunoglobulin Deposits (GOMMID)<br>• Intracapillary monoclonal IgM without cryoglobulin<br>• Intraglomerular/capillary lymphoma/leukaemia<br>• Light chain cast nephropathy<br>• Light chain proximal tubulopathy, crystalline<br>• Light chain proximal tubulopathy, non crystalline<br>• Monoclonal Immunoglobulin Deposition Disease (MIDD; includes Light Chain Deposition Disease - LCDD; Heavy Chair Deposition Disease - HCDD; and Light and Heavy Chain Deposition Disease - LHCDD)<br>• Proliferative glomerulonephritis with monoclonal immunoglobulin deposits – PGNMID<br>• Thrombotic Microangiopathy with monoclonal gammopathy<br>• Type 1 cryoglobulinaemic Glomerulonephritis<br>• Unclassified MGRS<br><br>*Patients with systemic amyloidosis may have a renal biopsy confirming AL amyloidosis or a biopsy of other tissue with confirmation of renal involvement by the UK National Amyloidosis Centre.	None Stated	Date of biopsy

RaDaR Inclusion and Exclusion Criteria

Diagnosis	Cohort	Inclusion Criteria	Exclusion Criteria	Date of Diagnosis
**Nephrogenic diabetes insipidus**	Tubulopathy	Nephrogenic diabetes insipidus<br><br>Genetically confirmed homozygous pathogenic variant in AVPR2, AQP2	None stated	Date that clinical diagnosis was first made
**Nephrogenic syndrome of inappropriate antidiuresis**	Tubulopathy	Nephrogenic syndrome of inappropriate antidiuresis<br><br>Genetically confirmed homozygous pathogenic variant in AVPR2	None stated	Date that clinical diagnosis was first made
**Nephronophthisis**	ARPKD/NPHP	Histological or radiological features of Nephronophthisis<br><br>Genetic diagnosis of Nephronophthisis or Nephronophthisis-related ciliopathy	None stated	Date that histological /radiological or genetic diagnosis was first made

RaDaR Inclusion and Exclusion Criteria

Diagnosis	Cohort	Inclusion Criteria	Exclusion Criteria	Date of Diagnosis
**Nephrotic Syndrome - Steroid Sensitive or Steroid Resistant**<br><br>**(Congenital nephrotic syndrome, nephrotic syndr ome with focal segmental glomerulosclerosis)**	INS	Children and adults with idiopathic Nephrotic Syndrome (nephrotic range proteinuria and hypoalbuminaemia)<br><br>Congenital NS (presumed Steroid Resistance)<br><br>Childhood or adult onset with primary Steroid Resistance<br><br>Childhood or adult onset with late onset Steroid Resistance<br><br>Steroid Sensitive Nephrotic Syndrome (full or partial remission in response to steroids)<br><br>As part of a syndrome e.g. Nail Patella Syndrome and Denys-Drash Syndrome<br><br>Those with a biopsy diagnosis of FSGS or minimal change disease can be included if they fall in the above categories but biopsy is not a prerequisite for inclusion	Secondary causes of Nephrotic Syndrome<br><br>• Primary diagnosis of Glomerulonephritis (IgA Nephropathy, Membranoproliferative Glomerulonephritis, Membranous Nephropathy)<br>• Vasculitis<br>• Systemic Lupus Erythematosus<br>• Diabetes<br>• Obesity<br>• Hypertension	Date of presentation to secondary or tertiary centre
**Oncogenic osteomalacia**	Tubulopathy	Oncogenic osteomalacia	None stated	Date that clinical diagnosis was first made
**Osteopetrosis with renal tubular acidosis**	Tubulopathy	Osteopetrosis with renal tubular acidosis<br><br>Genetically confirmed homozygous pathogenic variant in CA2	None stated	Date that clinical diagnosis was first made

Diagnosis	Cohort	Inclusion Criteria	Exclusion Criteria	Date of Diagnosis
**Polycystic Kidney Disease - Autosomal Dominant**	ADPKD	Clinical features of Autosomal Dominant Polycystic Kidney Disease meeting current image based diagnostic criteria<br><br>Clinical features compatible with ADPKD in the absence of a family history<br><br>Pathogenic or likely pathogenic PKD1 or PKD2 mutation with or without clinical features	Autosomal dominant polycystic liver disease with no evidence of renal cysts	Date that the clinical diagnosis was first made.<br><br>This may be reported by the clinician as the date of the diagnostic scan or by the patient if scans were performed at another centre
**Polycystic Kidney Disease - Autosomal Recessive**	ARPKD	Autosomal Recessive Polycystic Kidney Disease<br><br>Congenital Hepatic Fibrosis<br><br>Caroli Syndrome with kidney malformation or cyst	None stated	Date that clinical diagnosis was first made.

Diagnosis	Cohort	Inclusion Criteria	Exclusion Criteria	Date of Diagnosis
**Pregnancy and Chronic Kidney Disease**	Pregnancy	Pregnancy in all women known to have CKD 1-5 prior to pregnancy or those with a serum creatinine >85umol/l on two occasions during pregnancy<br><br>Pregnancy in all women with renal transplants regardless of function<br><br>Pregnancy in all women with previous or current lupus nephritis regardless of function	None stated	Date of last menstrual period
**Primary hypomagnesemia with secondary hypocalcemia**	Tubulopathy	Primary hypomagnesemia with secondary hypocalcemia<br><br>Genetically confirmed homozygous pathogenic variant in TRPM6	None stated	Date that clinical diagnosis was first made
**Pseudohypoaldosteronism type 2A**	Tubulopathy	Pseudohypoaldosteronism type 2A	None stated	Date that clinical diagnosis was first made
**Pseudohypoaldosteronism type 2B**	Tubulopathy	Pseudohypoaldosteronism type 2B<br><br>Genetically confirmed homozygous pathogenic variant in WNK1	None stated	Date that clinical diagnosis was first made
**Pseudohypoaldosteronism type 2C**	Tubulopathy	Pseudohypoaldosteronism type 2C<br><br>Genetically confirmed homozygous pathogenic variant in WNK4	None stated	Date that clinical diagnosis was first made
**Pseudohypoaldosteronism type 2D**	Tubulopathy	Pseudohypoaldosteronism type 2D<br><br>Genetically confirmed homozygous pathogenic variant in KLHL3	None stated	Date that clinical diagnosis was first made

Diagnosis	Cohort	Inclusion Criteria	Exclusion Criteria	Date of Diagnosis
**Pseudohypoaldosteronism type 2E**	Tubulopathy	Pseudohypoaldosteronism type 2E<br><br>Genetically confirmed homozygous pathogenic variant in CUL3	None stated	Date that clinical diagnosis was first made
**Pure Red Cell Aplasia**	PRCA	Treatment with any injectable form of erythropoiesis stimulating agent for at least four weeks.<br><br>Haemoglobin <70 g/l without transfusion or transfusion dependence.<br><br>Normal leucocyte and platelet count<br><br>Reticulocyte count < 20.000 / mm3<br><br>Bone marrow aspirate showing well preserved myeloid and megakaryocyte development, and <5% erythroblasts.<br><br>Presence of anti-erythropoietin antibodies.	Pre-established PRCA due to myeloproliferative disorder	Date of positive antibody test
**Renal pseudohypoaldosteronism type 1**	Tubulopathy	Renal pseudohypoaldosteronism type 1<br><br>Genetically confirmed homozygous pathogenic variant in NR3C2		Date that clinical diagnosis was first made

Diagnosis	Cohort	Inclusion Criteria	Exclusion Criteria	Date of Diagnosis
**Retroperitoneal Fibrosis**	Retroperitoneal Fibrosis	Any radiologically confirmed retroperitoneal fibrosis (RPF), presumed to be 'idiopathic' or associated with primary conditions including (but not exclusively):<br>• Aortitis<br>• Periaortitis<br>• IgG4-related Vasculitis<br>• Perivascular fibrosis<br>• Atherosclerotic or aneurysmal disease<br><br>**Note:** There is no specific ICD code for retroperitoneal fibrosis although the diagnosis term links to two ICD codes:<br>• ICD10:N13.5 - Crossing vessel and stricture of ureter without hydronephrosis<br>• ICD-9-CM 593.4 - Other ureteric obstruction	Neoplastic disease within retroperitoneal fibrosis mass defined histologically	Date of diagnostic imaging study report
**Sickle Cell Nephropathy**	**CKD Africa Genes**	People of African or Afro-Caribbean ancestry with CKD (KDIGO definition), >18 years<br><br>Known Sickle Cell disease with reduced kidney function, and/or blood or protein in urine with no other cause for kidney disease identified	None stated	Date that clinical diagnosis was first made

**Tuberous Sclerosis**	**Tuberous Sclerosis**	Clinical or molecular diagnosis of Tuberous Sclerosis Complex (TSC)<br><br>Multiple renal angiomyolipomas<br><br>Multiple renal angiomyolipomas (> 3) +/- pulmonary lymphangioleiomyomatosis (LAM) without other signs of TSC	None stated	Date that clinical diagnosis was first made

**Vasculitis (Primary systemic Vasculitis)**	**Vasculitis**	**Small vessel Vasculitis (ANCA associated)**<br><br>Microscopic polyangiitis (including renal limited Vasculitis)<br><br>Granulomatosis with polyangiitis (Wegener)<br><br>Eosinophilic granulomatosis with polyangiitis (Churg Strauss)<br><br>ANCA Vasculitis unclassified<br><br>**Small vessel Vasculitis (Immune complex)**<br><br>anti-GBM disease<br><br>Cryoglobulinemic Vasculitis<br><br>IgA Vasculitis (Henoch-Schönlein)<br><br>**Medium vessel Vasculitis**<br><br>Classical PAN<br><br>Kawasaki disease<br><br>**Large vessel Vasculitis**<br><br>Giant cell arteritis<br><br>Takayasu's arteritis	None stated	Date of biopsy.<br><br>In the absence of a biopsy, the date of a positive antibody test should be used

**Vasculitis (Primary systemic Vasculitis)**	Vasculitis	**Variable vessel Vasculitis**<br><br>Behçet's disease<br><br>Cogan's syndrome<br><br>**Single organ Vasculitis**<br><br>Isolated aortitis<br><br>Primary cerebral angiitis	None stated	Date of biopsy.<br><br>In the absence of a biopsy, the date of a positive antibody test should be used

**Inherited Renal Cancer Syndrome**	Renal Cancer Inherited	1. A molecular or clinical diagnosis according to standard criteria of any of the following conditions:<br><br>Von Hippel Lindau disease (VHL) OMIM 193300<br><br>PTEN hamartoma tumour syndrome (Cowden syndrome) OMIM 158350<br><br>Birt Hogg Dube syndrome (BHD) OMIM 135150<br><br>Hereditary leiomyomatosis and renal cell cancer syndrome(HLRCC) OMIM 150800<br><br>Succinate dehydrogenase-related tumour predisposition syndrome<br><br>BAP1-related tumour predisposition syndrome OMIM 614327<br><br>Hereditary Type 1 papillary renal cell carcinoma syndrome (MET oncogene) OMIM 605074<br><br>2. Two or more cases in first degree relatives of any type of renal cancer without an established molecular or clinical diagnosis<br>**3.** Bilateral, multiple primary renal cancers of any histopathological type with or with a family history	None stated	Date of molecular or clinical diagnosis according to standard criteria

STROBE checklist for reporting observational studies

	Item No	Recommendation
**Title and abstract**	1	**(*a*) Indicate the study's design with a commonly used term in the title or the abstract**<br><br>Page 1: "Cross-sectional analysis of [the UK RaDaR] Cohort"
		**(*b*) Provide in the abstract an informative and balanced summary of what was done and what was found**<br><br>Page 2: "RaDaR has automatic linkage with the UK Renal Registry (UKRR, with which all UK patients receiving Kidney Replacement Therapy (KRT) are registered). To assess for recruitment bias to RaDaR, ethnicity and socioeconomic status of 1) prevalent RaDaR patients receiving KRT were compared with patients with eligible rare disease diagnoses receiving KRT in the UKRR 2) patients recruited to RaDaR and all eligible unrecruited patients at two renal centres were compared 3) the age-stratified ethnicity distribution of RaDaR patients with Autosomal Dominant Polycystic Kidney Disease (ADPKD) was compared to the English Census."<br><br>And "We found evidence of some disparities in ethnicity and social deprivation in recruitment to RaDaR, however these were not consistent across all comparisons. Predominant rare kidney diseases in adults were ADPKD (29.2%), Vasculitis (15.8%) and IgA nephropathy (15.7%), compared to Idiopathic nephrotic syndrome (43.6%), Vasculitis (10.8%) and Alport Syndrome (5.9%) in children. Compared with either adults recruited to RaDaR or the English population, children recruited to RaDaR were more likely to be of Asian ethnicity and live in more socially deprived areas."
**Introduction**		
Background/rationale	2	**Explain the scientific background and rationale for the investigation being reported**<br><br>Page 2: ", there is a lack of high-quality published data on how [rare kidney diseases] present, and in which patient groups. Patients often face delays in diagnosis and lack of reliable information on their condition once diagnosed. The UK National Registry of Rare Kidney Diseases (RaDaR) was formed in 2010 to address this knowledge gap. It collects long-term data for UK patients with rare kidney conditions, and is the largest rare kidney disease registry in the world."
Objectives	3	**State specific objectives, including any prespecified hypotheses**<br><br>Page 2: "we present information about 25,880 adults and children recruited to RaDaR, including ethnicity, socioeconomic status, and kidney function, and investigate whether there is any bias in recruitment to RaDaR. To our knowledge, this is the largest epidemiological description of rare kidney diseases worldwide"
**Methods**		
Study design	4	**Present key elements of study design early in the paper**<br><br>Page 1: "cross-sectional cohort study"
Setting	5	**Describe the setting, locations, and relevant dates, including periods of recruitment, exposure, follow-up, and data collection**<br><br>Page 3: "RaDaR [was] set-up in 2010 by the UK Kidney Association (UKKA) with funding from the Medical Research Council, Kidney Care UK and Kidney Research

		UK, was designed to address these challenges by collecting longitudinal data for UK adults and children with rare kidney diseases. Uniquely embedded with the publicly funded National Health Service (NHS) to which all UK residents have free access, RaDaR is hosted by the UK Renal Registry (UKRR) and has UK-wide ethical approval as a research registry, enabling automated collection of retrospective and prospective data for patients across multiple regions."
Participants	6	**(*a*) *Cohort study*—Give the eligibility criteria, and the sources and methods of selection of participants. Describe methods of follow-up**<br><br>Page 4: "Eligibility criteria are available online at https://ukkidney.org/sites/renal.org/files/radar/Inclusion-Exclusion_april_2021_v22.pdf and as a Supplementary Appendix."<br><br>Page 6: "RaDaR recruits patients from 108 NHS sites (96 adult and 12 paediatric) across England (n=91), Scotland (n=9), Wales (n=3) and Northern Ireland (n=5)."<br><br>Page 4: "Data for RDGs with >85 patients recruited are presented"<br><br>Page 4: "Routine laboratory data are extracted via automated feed either directly from renal unit IT systems or via the UK Renal Data Collaboration."
Variables	7	**Clearly define all outcomes, exposures, predictors, potential confounders, and effect modifiers. Give diagnostic criteria, if applicable**<br><br>N/A
Data sources/ measurement	8*	**For each variable of interest, give sources of data and details of methods of assessment (measurement). Describe comparability of assessment methods if there is more than one group**<br><br>Page 6: "Laboratory results were uploaded from participants' clinical records; frequency of tests varied between participants, diseases and centres.<br><br>Page 6: "Sex is reported according to UKRDC record."
Bias	9	**Describe any efforts to address potential sources of bias**<br><br>Page 2: "To assess for recruitment bias to RaDaR, ethnicity and socioeconomic status of 1) prevalent RaDaR patients receiving KRT were compared with patients with eligible rare disease diagnoses receiving KRT in the UKRR 2) patients recruited to RaDaR and all eligible unrecruited patients at two renal centres were compared 3) the age-stratified ethnicity distribution of RaDaR patients with Autosomal Dominant Polycystic Kidney Disease (ADPKD) was compared to the English Census."
Study size	10	**Explain how the study size was arrived at**<br><br>Page 4: "Data for 20 RDGs with >85 patients are presented"
Quantitative variables	11	**Explain how quantitative variables were handled in the analyses. If applicable, describe which groupings were chosen and why**<br><br>Page 5: "[data were] presented as frequencies (%) for categorical data and medians (IQR) for continuous data."

Statistical methods	12	**(*a*) Describe all statistical methods, including those used to control for confounding**<br>Page 5: See section "Statistical Analyses"
		**(*b*) Describe any methods used to examine subgroups and interactions**<br>Page 5: "Chi-square or Fishers exact tests were used to compare categorical variables"
		**(*c*) Explain how missing data were addressed**<br>Supplementary Tables 1-3
		**(*d*) *Cohort study*—If applicable, explain how loss to follow-up was addressed**<br>n/a<br>~~*Case-control study*—If applicable, explain how matching of cases and controls was addressed~~<br>~~*Cross-sectional study*—If applicable, describe analytical methods taking account of sampling strategy~~
		**(*e*) Describe any sensitivity analyses**<br>N/A

Continued on next page

**Results**		
Participants	13*	**(a) Report numbers of individuals at each stage of study—eg numbers potentially eligible, examined for eligibility, confirmed eligible, included in the study, completing follow-up, and analysed**<br><br>Page 6: "Clinical characteristics of 25,880 prevalent patients in RaDaR on 25th July 2022 are displayed"
		**(b) Give reasons for non-participation at each stage** n/a – informed consent from all participants
		**(c) Consider use of a flow diagram**<br><br>Figure 1
Descriptive data	14*	**(a) Give characteristics of study participants (eg demographic, clinical, social) and information on exposures and potential confounders**<br><br>Page 6: See Results section
		**(b) Indicate number of participants with missing data for each variable of interest**<br><br>Supplementary Tables 1-3
		**(c) *Cohort study*—Summarise follow-up time (eg, average and total amount)**<br><br>N/A
Outcome data	15*	~~*Cohort study*—Report numbers of outcome events or summary measures over time~~
		~~*Case-control study*—Report numbers in each exposure category, or summary measures of exposure~~
		Cross-sectional study—Report numbers of outcome events or summary measures<br><br>Page 6: See Results section
Main results	16	**(*a*) Give unadjusted estimates and, if applicable, confounder-adjusted estimates and their precision (eg, 95% confidence interval). Make clear which confounders were adjusted for and why they were included**<br><br>N/A
		(*b*) **Report category boundaries when continuous variables were categorized**<br><br>See supplementary Figure 5
		**(*c*) If relevant, consider translating estimates of relative risk into absolute risk for a meaningful time period** n/a
Other analyses	17	**Report other analyses done—eg analyses of subgroups and interactions, and sensitivity analyses**<br><br>All analyses performed are reported

**Discussion**		
Key results	18	**Summarise key results with reference to study objectives**<br><br>Page 8: "We have presented cross-sectional analyses for 1,934 (7%) paediatric and 23,946 (93%) adult patients with rare kidney diseases enrolled into RaDaR."

Limitations	19	**Discuss limitations of the study, taking into account sources of potential bias or imprecision. Discuss both direction and magnitude of any potential bias**<br><br>Page 10: "Limitations of this study include the fact that RaDaR is a UK registry and is representative of the mainly White UK population and may not be generalisable to other ethnicities. Survivor bias may have had an impact on the enrolment of individuals with diagnoses made before RaDaR started recruiting patients with that condition. Some RaDaR diagnoses are poorly captured by ERA-EDTA PRD codes, which limited comparison to UKRR data. Entry of rare disease diagnoses into renal IT systems is user dependent and may vary between renal units used for comparisons. Although bias could be introduced owing to the variation in recruitment between centres across the UK, and therefore by variation in their catchment population, we sought to minimise effects of this bias by comparing ethnicity and SES of each RDG to the overall RaDaR breakdown. Caution must still be exercised where clinicians with particular interest in a certain RDG recruit more intensively into that one RDG compared to others."
Interpretation	20	**Give a cautious overall interpretation of results considering objectives, limitations, multiplicity of analyses, results from similar studies, and other relevant evidence**<br><br>Page 10: "In summary, to our knowledge RaDaR is the largest registry of rare kidney diseases worldwide and provides numerous opportunities to advance understanding of rare kidney diseases, including identification of potential participants in clinical trials."
Generalisability	21	**Discuss the generalisability (external validity) of the study results**<br><br>Page 10: Limitations (as above)
**Other information**		
Funding	22	**Give the source of funding and the role of the funders for the present study and, if applicable, for the original study on which the present article is based**<br><br>Page 11: See Disclosures Statement

*Give information separately for cases and controls in case-control studies and, if applicable, for exposed and unexposed groups in cohort and cross-sectional studies.

Note: An Explanation and Elaboration article discusses each checklist item and gives methodological background and published examples of transparent reporting. The STROBE checklist is best used in conjunction with this article (freely available on the Web sites of PLoS Medicine at http://www.plosmedicine.org/, Annals of Internal Medicine at http://www.annals.org/, and Epidemiology at http://www.epidem.com/). Information on the STROBE Initiative is available at www.strobe-statement.org.
